# Association of sugar consumption with risk of depression and anxiety: a systematic review and meta-analysis

**DOI:** 10.3389/fnut.2024.1472612

**Published:** 2024-10-16

**Authors:** JiaHui Xiong, Lu Wang, HongLu Huang, San Xiong, ShiPeng Zhang, QinWei Fu, Rui Tang, QinXiu Zhang

**Affiliations:** ^1^Clinical Medical College, Chengdu University of Traditional Chinese Medicine, Chengdu, China; ^2^Department of Rheumatology and Clinical Immunology, Peking Union Medical College Hospital, Chinese Academy of Medical Sciences, Peking Union Medical College, Beijing, China; ^3^Department of Psychiatry and Psychotherapy, TUM School of Medicine and Health, Klinikum rechts der Isar, Technical University of Munich, Munich, Germany; ^4^Hospital of Chengdu University of Traditional Chinese Medicine, Chengdu, China

**Keywords:** sugar consumption, diet, depression, anxiety, meta-analysis

## Abstract

**Background:**

Sugar consumption has increased dramatically around the world, and at the same time, the prevalence of mental illnesses such as depression and anxiety continues to increase. While previous research has explored the impact of various dietary factors on mental health, the specific impact of dietary sugar consumption on the risk of depression and anxiety disorders remains elusive. This study aimed to comprehensively assess this relationship through a systematic review and meta-analysis.

**Methods:**

PubMed, Embase, Cochrane Library, Web of Science, China National Knowledge Network (CNKI), and WangFang were systematically searched for studies of the association between total dietary sugar intake and risk of depression and/or anxiety. The articles that meet the criteria are screened and included in the systematic review, and the data are extracted after assessing their quality. Stata 18.0 software was used for the meta-analysis.

**Results:**

Forty studies with 1,212,107 participants were included in the analysis. Results showed that sugar intake increased the risk of depression by 21% (OR = 1.21, 95% CI: 1.14, 1.27), while the overall association between sugar intake and anxiety risk was not statistically significant (OR = 1.11, 95% CI: 0.93, 1.28). Despite high heterogeneity (*I*^2^ = 99.7%), the results were statistically significant (*p* < 0.000). Subgroup analyses showed that the association between sugar consumption and depression risk remains consistent across different study designs (cross-sectional, cohort, and case–control studies) and different sample sizes (<5,000, 5,000–10,000, >10,000). Women have a higher risk of depression than men (OR = 1.19, 95% CI: 1.04, 1.35). Among the different exposure measures, the Food Frequency Questionnaire (FFQ) showed the most significant effect (OR = 1.32, 95% CI: 1.08, 1.67, *I*^2^ = 99.7%, *p* < 0.000). The measuring tool of subgroup analysis showed that there was a significant correlation between sugar intake and risk of depression, PHQ-9 (OR = 1.29, 95% CI: 1.17, 1.42, I^2^ = 86.5%, *p* < 0.000), and CES-D (OR = 1.28, 95% CI: 1.14, 1.44, *I*^2^ = 71.3%, *p* < 0.000). High-quality cross-sectional and cohort studies showed a significant association between sugar intake and depression risk, with most results being robust. While the overall analysis of sugar intake and anxiety risk was not significant, some subgroups approached significance, particularly in studies with a sample size of <5,000 (OR = 1.14, 95% CI: 0.89, 1.46) and studies using the FFQ questionnaire (OR = 1.31, 95% CI: 0.90, 1.89).

**Conclusion:**

Total dietary sugar consumption was significantly associated with increased risk of depression in the general population, whereas the association with risk of anxiety was not significant. Further high-quality studies are needed to verify these associations and ensure their reliability. This study highlights the impact of dietary sugar intake on mental health, identifies potentially high-risk groups through subgroup analysis, and provides new insights into the prevention of depression and anxiety.

**Systematic review registration:**

CRD42024540548.

## 1 Introduction

In recent years, with the improvement of living standards and the development of the food industry, high-sugar diets have become increasingly prevalent worldwide. Significant increases in the intake of high-sugar foods such as sweetened beverages, desserts, and processed foods have contributed not only to metabolic health issues such as obesity, diabetes, and cardiovascular disease but also to mental health problems. Interestingly, individuals often seek pleasure and solace in sweets (1, 2), and many studies have shown that people with high-sugar diets have a high prevalence of mental disorders such as depression (3). Globally, more than 970 million people suffer from mental disorders, with depression and anxiety being the most common (4). The World Health Organization (WHO) reports that about 5% of adults worldwide suffer from depression, and 4% of the population experiences anxiety (5, 6). In 2020, the lifetime prevalence of depression and anxiety disorders was 18.4 and 34%, respectively, in the United States (7, 8). Depression and anxiety have become major contributors to the burden of mental health-related illnesses. They cause significant suffering and distress to individuals, increase the burden on medical resources, and adversely affect economic and social security and development.

Studies have shown that mental disorders are caused by complex interactions, including social and psychological factors, and that poor lifestyle choices can increase the risk of developing these conditions (9, 10). The effects of sugar intake on mental disorders have become a matter of particular concern. Excessive sugar intake has been shown to harm human health (11), but the potential benefits or harms within the range of daily intake are poorly understood. Sugar intake appears to affect mood swings by affecting blood sugar or causing an inflammatory response in the nervous system. The evidence from studies and reviews that have been conducted is limited and contradictory. A cross-sectional study of adults found a positive correlation between dietary sugar intake and depression, with every 100 g of dietary sugar intake per day increasing the incidence of depression by 28% (12). Although these mechanisms suggest a possible association between a high-sugar diet and depression and anxiety, several studies have yielded contrary results. For instance, a 10-year follow-up study involving 15,546 participants found no significant association between the consumption of sugar-sweetened beverages and depressive symptoms (13). In one systematic review, a daily intake of ≥5 g of fructooligosaccharides and galacto-oligosaccharides improved anxiety and depression in participants (14). Therefore, this study aims to investigate the association between total sugar intake and depression and anxiety disorders and to provide more definitive and consistent conclusions based on the available evidence. Therefore, the aim of this study was to investigate the association between total sugar intake and depression and anxiety and to synthesize the existing evidence to provide more clear and consistent conclusions. This systematic review and meta-analysis aimed to systematically assess the association between total sugar intake and the risk of depression and anxiety by examining the following questions:

- What is the effect of sugar intake/higher intake versus no/lower intake on depression in the general population?
- What are the effects of sugar intake and no intake on depression according to study type, sex, sample size, exposure instrument, measuring instrument, study quality, and region?
- What are the effects of sugar intake/higher intake versus no/lower intake on anxiety in the general population?
- What are the effects of sugar intake and no intake on anxiety according to study type, sex, sample size, exposure instrument, measuring instrument, study quality, and region?

## 2 Methods

### 2.1 Search strategy

The authors, JX and HH, conducted an electronic database search in PubMed, Embase, Web of Science, Cochrane Library, CNKI, and WangFang up to May 2024. After removing duplicates, articles were screened based on their titles and abstracts. Another author, LW, further screened the search results by title and abstract using broad inclusion criteria to temporarily retain all potentially relevant documents. JX and HH then independently conducted a full-text review to determine eligibility, resolving discrepancies through discussion or third-party arbitration. Concurrently, JX and HH manually reviewed the reference lists of included articles for potential missing documents. In particular, references for systematic reviews and meta-analyses, and search strategies for electronic databases, are provided in Supplementary Table S1.

### 2.2 Inclusion and exclusion criteria

According to the recommendation (15), the PECO(S) framework (16) was used to determine the review questions: Inclusion Criteria: (1) P-Population: The general population, including individuals who are obese or overweight, as well as patients diagnosed with depression and anxiety disorders. There are no restrictions on age, gender, or ethnicity, but the characteristics of the population must be clearly reported. (2) E-Exposure: Exposure includes sugars of any origin and form, including natural sugars and added sugars, such as desserts, sugar-sweetened beverages (soft drinks, fruit juices), and sugary foods (sweets, pastries). Whether it is a single sugar or a combination of multiple sugars, the source of the sugar must be clear, and the study must provide detailed information about sugar intake (e.g., intake, frequency, etc.). (3) C-Compare: A control group relevant to the purpose of the study, which can effectively assess the impact of the intervention. (4) O-Outcome measures: Depression and/or anxiety measured at the end of the study period, based on standardized professional questionnaires or measuring tools (such as PHQ-9, CES-D, HADS, GAD-7, etc.). (5) S-Study Design Protocol: Observational studies, including cohort, case–control, and cross-sectional studies. Published peer-reviewed research. The odds ratio (OR), relative risk (RR), prevalence ratio (PR), or hazard ratio (HR) with their 95% confidence intervals must be directly provided in the article, or the data provided must be sufficient to calculate the OR indirectly. (6) Literature language: Only English and Chinese literature are included.

Exclusion criteria: (1) Studies using non-sugar sweeteners (NSS) were excluded. (2) Individuals with diseases other than depression and anxiety disorders (such as cardiovascular diseases and diabetes) were excluded. (3) Studies involving pregnant and lactating women were excluded. (4) Studies involving subjects known to affect appetite or emotional state (such as those taking antidepressants or anxiolytics) were excluded. (5) Unpublished studies, reviews, conference abstracts, and case reports were excluded.

### 2.3 Study selection and characteristics of included studies

**Figure 1:**
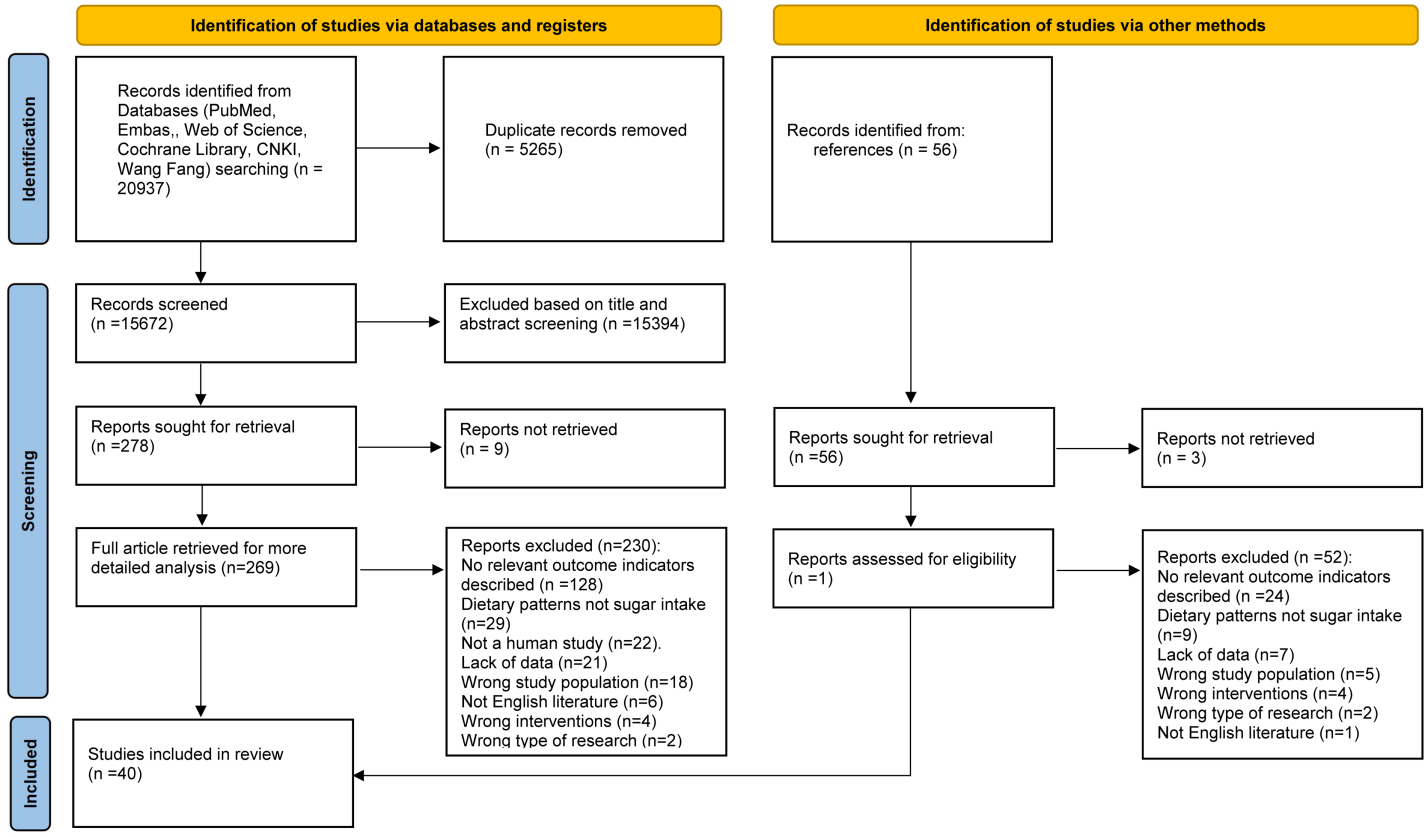
Flow chart for meta-analysis.

An electronic database search identified and screened 20,937 publications. After removing 5,265 duplicate studies, 15,394 studies were excluded because they were not relevant to the topic. A total of 278 articles proceeded to the full-text qualification review stage. Of these, 9 articles were excluded due to the absence of full text, and another 230 were excluded for not meeting the inclusion criteria (Supplementary Table 2). The final 39 studies were eligible for inclusion, and one additional study was added based on reference tracking, resulting in a total of 40 studies: 33 cross-sectional studies (9, 17–48), 6 cohort studies (49–54), and 1 case–control study (55) (Figure 1). The key characteristics of all included studies are detailed in Supplementary Table 3.

### 2.4 Data extraction

Two authors (JX and SX) independently extracted data from each of the included studies and cross-checked the extracted study characteristics. Any discrepancies related to data extraction were resolved by re-examining the full study text or through discussion. We contacted the original authors to obtain relevant information not reported in the original manuscript. The extracted details included the following: the name of the first author; study site; the origin and number of participants; mean or median age, education level, and BMI of participants; duration of follow-up; details of sugar intake, such as amount, frequency, and type (e.g., soft drinks, desserts); outcome assessment on depression and anxiety, including measuring tools (e.g., PHQ-9, CES-D) and effect sizes (e.g., OR, RR) with their 95% confidence intervals (CIs) for all categories of sugar intake in each study, as well as covariates for adjustment. Data extraction followed the methods recommended by the Cochrane Reviewer’s Handbook (56).

### 2.5 Review of literature quality/risk of bias

Two review authors (LW and JX) independently assessed the risk of bias for each study. Any disagreements were resolved through discussion or by involving a third author (HW). For non-randomized controlled trials, the quality of the included studies was independently assessed using the Newcastle-Ottawa Quality Assessment Scale (NOS) (57). For cross-sectional studies, the quality of the included studies was independently assessed using the AHRQ (Agency for Healthcare Research and Quality) tool. The scores for each included study are presented in Supplementary Table 4. When data from five or more studies were available, funnel plots were planned to assess the likelihood of publication bias.

### 2.6 Statistical analysis

Meta-analyses were performed by pooling the calculated effect sizes [odds ratios (OR) and risk ratios] and their 95% confidence intervals (CIs). We used the *I*^2^ statistic to assess heterogeneity between the included studies (58). When heterogeneity was low (*I*^2^ < 50%), we applied a fixed-effects model. When heterogeneity was high (*I*^2^ ≥ 50%), we employed a random-effects model. The robustness of our results was tested through sensitivity analyses by excluding each study individually and recalculating the composite effect size to determine if there was a significant change. Results were considered robust if there was no significant change after the exclusion of any study (59). Subgroup analyses were performed to explore sources of heterogeneity (58), such as gender, region, study type, sample size, exposure, and outcome variable measurement methods, among other factors (60). In the forest plot, results from analyses using a random-effects model were reported as our primary effect estimate. All statistical analyses were conducted using STATA software (Version 18, StataCorp LP, College Station, Texas, United States). During the translation process of this article, the ChatGPT model developed by OpenAI (based on the GPT-4 architecture, version: GPT-4, released in 2023) was used. This model utilized natural language processing techniques to generate and optimize the translated content. The generated text was manually reviewed to ensure it meets the writing requirements of this article (Supplementary Figure 1).

### 2.7 Analysis of sensitivity and publication bias

We performed sensitivity analyses using a random-effects model and conducted meta-analyses where data were eligible. To explore publication bias, we employed Egger’s test and Begg’s test. Funnel plots were generated only for meta-analyses involving more than five studies, considering the clinical heterogeneity and statistical significance of the included studies (17) (Supplementary Figures 2,3).

## 3 Results

### 3.1 Sugar intake and risk of depression

**Figure 2:**
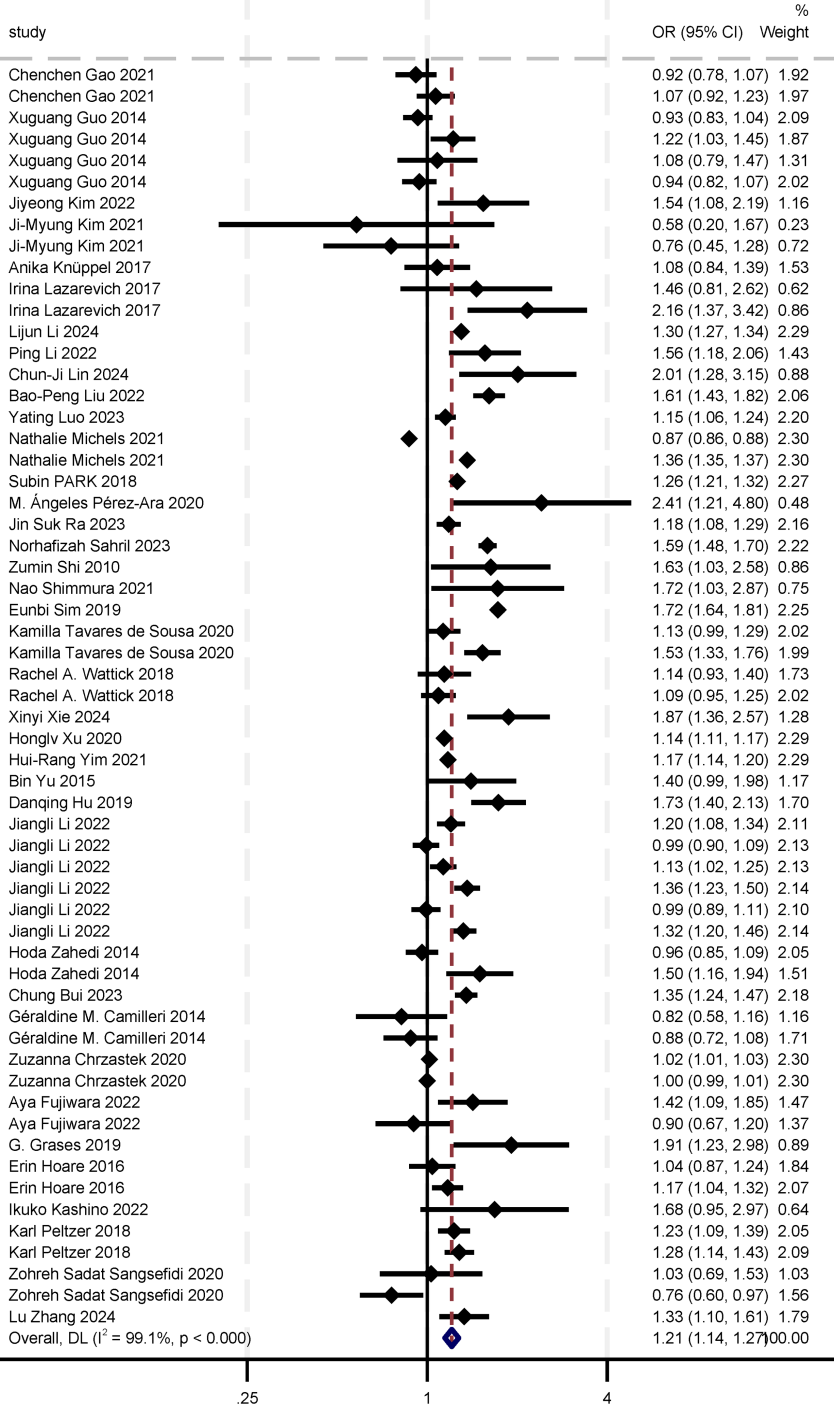
Results of overall and subgroup meta-analysis of sugar intake and depression.

In the meta-analysis of the association between sugar intake and depression, we included 38 studies: 32 cross-sectional studies, five cohort studies, and one case–control study. The eligible studies comprised 1,157,851 participants, with NOS or AHRQ scores ranging from 6 to 10 for individual studies (Supplementary Table S3). Summary analyses comparing the highest and lowest categories of sugar intake demonstrated a significant increase in the risk of depression by 21% [OR 1.21 (95% CI: 1.14, 1.27)] (Table 1; Figure 2). Sensitivity analyses confirmed the robustness of these results, with significant results persisting even after individual studies were removed and effect estimates re-evaluated. Considering the high heterogeneity of the analysis (*I*^2^ = 99.1%), we re-analyzed the effect estimation by stratifying according to study design, gender, sample, exposure measures, outcome assessment, study quality, and region. This approach aimed to explore specific characteristics and mechanisms of the association between sugar intake and depression risk, thereby enhancing the comprehensiveness, reliability, and explanatory power of the study results and providing a deeper and more nuanced understanding of the relationship between sugar intake and depression risk (Table 1).

**Table 1:** Results of association between sugar intake and risk of depression.

Analysis type	Subgroup	Number of studies	Pooled OR	*I*^2^ (%)	*p*-value	Number of patients	Egger’s test	Begg’s test	Sensitivity analyses (robust or not)
Meta-analysis results	Overall	38	1.21 (1.14, 1.27)	99.1	<0.000	11,578,51	0.313	0.278	Yes
Subgroup analysis by study designing	Cohort	5	1.14 (1.00, 1.29)	70.8	<0.000	268,511	0.036	0.175	Yes
	Cross-sectional	32	1.21 (1.14, 1.28)	99.3	<0.000	889,208	0.34	0.093	Yes
	Case–control	1	1.91 (1.23–2.99)	NA	NA	132	NA	NA	NA
Subgroup analysis by gender	Male	11	1.08 (0.97, 1.20)	98.8	<0.000	44,788	0.468	0.244	Yes
	Female	11	1.19 (1.04, 1.35)	99.7	<0.000	38,708	0.806	0.246	Yes
Subgroup analysis by sample size	<5,000	16	1.14 (1.01, 1.33)	99.6	<0.000	33,789	0.935	0.118	No
	5,000–10,000	4	1.12 (1.01, 1.25)	81.7	<0.000	33,825	0.585	0.371	Yes
	>10,000	18	1.25 (1.18, 1.32)	94.2	<0.000	10,902,37	0.557	0.981	Yes
Subgroup analysis by exposure measures	FFQ	9	1.32 (1.08, 1.67)	99.7	<0.000	76,669	0.866	0.583	Yes
	24-h dietary recall	6	1.03 (1.00, 1.07)	86.3	< 0.000	88,585	0.344	0.754	No
	Self-made questionnaire	24	1.20 (1.14, 1.26)	92	<0.000	99,473	0.896	0.942	Yes
Subgroup analysis by outcome assessment	PHQ-9	11	1.29 (1.17, 1.42)	86.5	<0.000	312,623	0.83	0.827	No
	CES	10	1.28 (1.14, 1.44)	71.3	<0.000	103,782	0.748	0.584	Yes
	Others (CDI, DASS, BDI, SDS, etc.)	17	1.15 (1.08, 1.24)	99.5	<0.000	741,446	0.689	0.563	Yes
Subgroup analysis by quality of study	Cross-sectional								
	6	1	1.173 (1.144, 1.202)	NA	NA	187,622	NA	NA	NA
	7	15	1.20 (1.14, 1.27)	97.4	<0.000	317,322	0.005	0.031	Yes
	8	10	1.27 (1.09, 1.49)	99.7	<0.000	321,983	0.924	0.661	Yes
	9	5	1.24 (1.01 1.53)	79.8	0.004	32,041	0.086	0.133	Yes
	10	1	0.86 (0.72, 1.03)	0.00	0.736	30,240	NA	NA	NA
	Cohort								
	7	4	1.09 (0.97, 1.22)	64.2	0.007	266,951	0.117	0.386	Yes
	8	1	2.01 (1.28, 3.15)	NA	NA	1,560	NA	NA	NA
	Case–control								
	8	1	1.91 (1.23–2.99)	NA	NA	132	NA	NA	NA
Subgroup analysis by region	Asia	22	1.22 (1.16, 1.29)	92.3	<0.000	664,641	0.861	0.988	Yes
	European	6	1.08 (0.93, 1.24)	99.9	<0.000	46,708	0.864	0.175	Yes
	North America	7	1.27 (1.10, 1.47)	86.4	<0.000	394,342	0.201	0.193	Yes
	Australia	2	1.16 (0.99, 1.35)	43.4	0.171	5,375	NA	NA	NA
	South America	1	1.31 (0.98, 1.77)	89.5	0.02	46,785	NA	NA	NA

NA, not available (because the number of studies is not enough to calculate).

#### 3.1.1 Subgroup analysis by study designing

Further subgroup analyses were categorized by study design. The five cohort studies showed the overall OR = 1.14 (95% CI: 1.00, 1.29), with *I*^2^ = 70.8% (*p* < 0.000), and included 268,511 participants. Thirty-two cross-sectional studies showed the overall OR = 1.21 (95% CI: 1.14, 1.28), with *I*^2^ = 99.3% (*p* < 0.000), and involved 889,208 participants. The case–control analysis comprised a single study, with the OR = 1.91 (95% CI: 1.23, 2.99) and a patient count of 132. Sensitivity analyses demonstrated robust results across all subgroups (Table 1).

#### 3.1.2 Subgroup analysis by gender

Analyses by gender group found that sugar intake increased the risk of depression in women by 19% (OR = 1.19, 95% CI: 1.04, 1.35). However, the effect on depression in men was not statistically significant (OR = 1.08, 95% CI: 0.97, 1.20; Table 1).

#### 3.1.3 Subgroup analysis by sample size

In a subgroup analysis by sample size, the subgroup with a sample of <5,000, based on 16 studies, had OR = 1.14 (95% CI: 1.01, 1.33) for the association between sugar intake and the risk of depression, with *I*^2^ = 99.6% and *p* < 0.000. However, this result was not considered robust in sensitivity analysis. For the subgroup with a sample of 5,000–10,000, the analysis of four studies revealed the overall OR of 1.12 (95% CI: 1.01, 1.25), with *I*^2^ = 81.7% and *p* < 0.000; this result was considered robust in sensitivity analysis. For the subgroup with a sample > 10,000, the analysis of 18 studies showed the OR = 1.25 (95% CI: 1.18, 1.32), with *I*^2^ = 94.2% and *p* < 0.000; these results were considered robust in sensitivity analysis. These findings further support a significant association between sugar intake and depression risk and suggest that this association is consistent across different samples, particularly in large population-based studies, where the association is notably more significant and robust (Table 1).

#### 3.1.4 Subgroup analysis by exposure measures

In subgroup analyses classified by exposure measurement tools, nine studies that utilized the Food Frequency Questionnaire (FFQ) showed the OR = 1.32 (95% CI: 1.08, 1.67) for the association between sugar intake and the risk of depression, with *I*^2^ = 99.7% and *p* < 0.000; these results were considered robust in sensitivity analyses. In six studies employing a 24-h dietary recall method, the overall OR for sugar intake and depression risk was 1.03 (95% CI: 1.00, 1.07), with *I*^2^ = 86.3% and *p* < 0.000, but these results were not considered robust in sensitivity analyses. Twenty-four studies using self-made questionnaires demonstrated the overall OR = 1.20 (95% CI: 1.14, 1.26) for sugar intake and depression risk, with *I*^2^ = 92% and *p* < 0.000; these results were considered robust in sensitivity analyses. These findings indicate significant differences in the association between sugar intake and depression risk depending on the exposure measurement tool used, particularly in studies using FFQ and self-made questionnaires. This further supports the significant association between sugar intake and depression risk and underscores the importance of considering the impact of the measurement tool on study outcomes (Table 1).

#### 3.1.5 Subgroup analysis by outcome assessment

In subgroup analyses by measuring questionnaires, 11 studies utilizing the PHQ-9 for measurement showed the OR = 1.29 (95% CI: 1.17, 1.42) for the association between sugar intake and the risk of depression, with *I*^2^ = 86.5% and *p* < 0.000; however, these results were not deemed robust in sensitivity analyses. The 10 studies employing the CES-D demonstrated OR = 1.28 (95% CI: 1.14, 1.44), with I^2^ = 71.3% and *p* < 0.000; these results were considered robust in sensitivity analyses. Seventeen studies utilizing other measuring tools (e.g., CDI, DASS, BDI, SDS) showed the OR = 1.15 (95% CI: 1.08, 1.24), with *I*^2^ = 99.5% and *p* < 0.000; these results were considered robust in sensitivity analyses. These findings suggest significant differences in the association between sugar intake and depression risk across different measuring questionnaires, particularly in studies employing the CES-D and other measuring questionnaires (Table 1).

#### 3.1.6 Subgroup analysis by quality of study

In cross-sectional studies, one study with a quality score of 6 showed the OR = 1.173 (95% CI: 1.144, 1.202) for the association between sugar intake and the risk of depression, including 187,622 participants. Of the 15 studies with a quality score of 7, the overall OR was 1.20 (95% CI: 1.14, 1.27), with *I*^2^ = 97.4% and *p* < 0.000; sensitivity analyses indicated robust results, with Egger’s test (*p* = 0.005) and Begg’s test (*p* = 0.031) suggesting no significant publication bias. Among the 10 studies with a quality score of 8, the overall OR was 1.27 (95% CI: 1.09, 1.49), with *I*^2^ = 99.7% and *p* < 0.000; sensitivity analyses were robust, although Egger’s test (*p* = 0.924) and Begg’s test (*p* = 0.661) indicated potential publication bias. In the five studies with a quality score of 9, the overall OR was 1.24 (95% CI: 1.01, 1.53), with *I*^2^ = 79.8% and *p* = 0.004; sensitivity analyses were robust, with Egger’s test (*p* = 0.086) and Begg’s test (*p* = 0.133) indicating acceptable publication bias. A single study with a quality score of 10 showed a composite OR of 0.86 (95% CI: 0.72, 1.03), with *I*^2^ = 0.00% and a *p* = 0.736. In cohort studies, four studies with a quality score of 7 showed the overall OR = 1.09 (95% CI: 0.97, 1.22) for sugar intake and the risk of depression, with *I*^2^ = 64.2% and *p* = 0.007; sensitivity analyses were robust, with Egger’s test (*p* = 0.117) and Begg’s test (*p* = 0.386) suggesting some publication bias. A single study with a quality score of 8 showed the OR = 2.01 (95% CI: 1.28, 3.15). In the case–control study, one study with a quality score of 8 showed the OR = 1.91 (95% CI: 1.23, 2.99) for sugar intake and the risk of depression. Overall, cross-sectional and cohort studies with high-quality scores demonstrated a significant association between sugar intake and the risk of depression, with results being robust in most cases (Table 1).

#### 3.1.7 Subgroup analysis by region

In a subgroup analysis by region, 22 studies conducted in Asia reported the OR = 1.22 (95% CI: 1.16, 1.29) for the association between sugar intake and anxiety risk. The heterogeneity (*I*^2^) was 92.3%, with *p* < 0.000, and the studies included 664,641 participants. The *p*-values of Egger’s test and Begg’s test were 0.861 and 0.988. Six studies conducted in Europe showed the OR = 1.08 (95% CI: 0.93, 1.24) for sugar intake and anxiety, with *I*^2^ = 99.9%, p < 0.000, including 46,708 patients, The *p*-values of Egger’s test and Begg’s test were 0.864 and 0.175. Seven studies conducted in North America showed that the OR = 1.27 (95% CI: 1.10, 1.47) for the association between sugar intake and anxiety risk, with *I*^2^ = 86.4%, p < 0.000, including 394,342 patients. Egger’s test and Begg’s test were 0.201 and 0.193, respectively. Results show that the above three groups found no significant publication bias, and sensitivity analysis results have shown the steady. No significant publication bias was found in the results of the above three groups, and sensitivity analysis showed robust results. Two Australian studies showed the OR = 1.16 (95% CI: 0.99, 1.35), with *I*^2^ = 43.4%, *p* = 0.171 for sugar intake and anxiety risk in 5,375 patients. One study in South America showed the OR = 1.31 (95% CI: 0.98, 1.77), with *I*^2^ = 89.5%, *p* = 0.02 for sugar intake and anxiety risk in 46,785 patients. Analysis of publication bias and sensitivity could not be performed due to the limited number of studies included in the Australian and South American regional groups (Table 1).

### 3.2 Sugar intake and risk of anxiety

**Figure 3:**
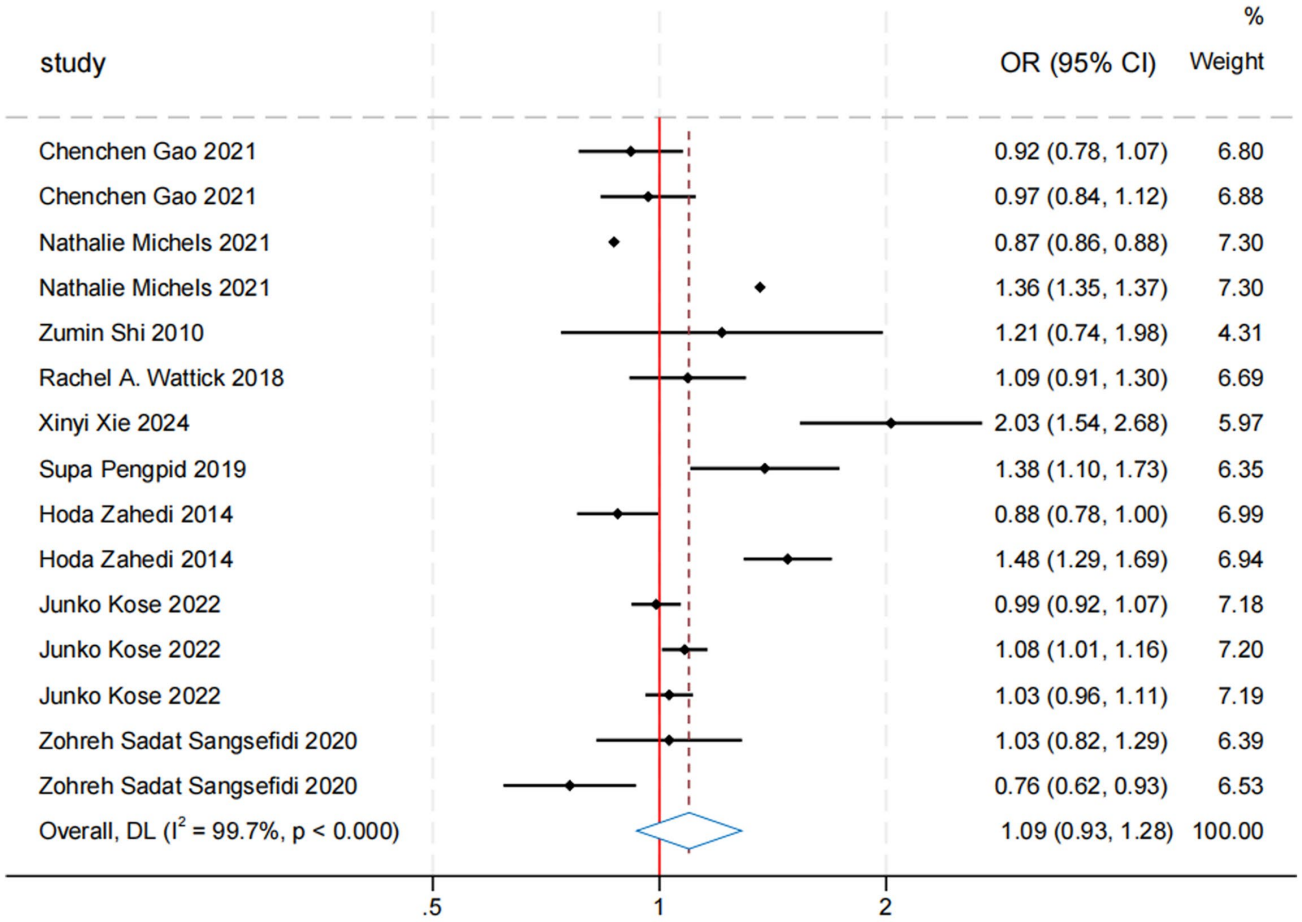
Results of overall and subgroup meta-analysis of sugar intake and anxiety.

In the meta-analysis of sugar intake and anxiety risk, nine studies covering 88,046 participants were included. The overall odds ratio (OR) for the association between sugar intake and anxiety risk was 1.11 (95% CI: 0.93, 1.28; Table 2; Figure 3). Although the results were statistically significant, the high heterogeneity (*I*^2^ = 99.7%, *p* < 0.000) indicates variability among the included studies. The Egger’s test and Begg’s test yielded *p*-values of 0.531 and 0.138, respectively, suggesting no significant publication bias. Sensitivity analyses further confirmed the robustness of the findings. While a composite OR > 1 suggests that sugar intake may be associated with an increased risk of anxiety, the 95% confidence interval encompassing 1 means that the association could be due to random error. Thus, current evidence is insufficient to definitively conclude that sugar intake significantly increases anxiety risk. Further high-quality studies are needed to validate this association and investigate potential mechanisms and moderating factors. Additionally, we re-analyzed the effect estimation by stratifying the study design, gender, sample, exposure measures, measuring methods, study quality, and region (Table 2).

**Table 2:** Results of association between sugar intake and risk of anxiety.

Analysis type	Subgroup	Number of studies	Pooled OR	*I*^2^ (%)	*p*-value	Number of patients	Egger’s test	Begg’s test	Sensitivity analyses **(**robust or not)
Meta-analysis results	Overall	9	1.11 (0.93, 1.28)	99.7	<0.000	88,046	0.531	0.138	Yes
Subgroup analysis by study design	Cohort	1	1.03 (0.98, 1.09)	27.9	0.25	15,602	NA	NA	NA
	Cross-sectional	8	1.11 (0.92–1.34)	99.7	<0.000	72,444	0.651	0.304	Yes
Subgroup analysis by sex	Male	2	0.96 (0.77, 1.19)	83.6	0.013	2,878	NA	NA	NA
	Female	1	1.36 (1.34–1.36)	NR	NR	3,097	NA	NA	NA
Subgroup analysis by sample size	<5,000	5	1.14 (0.89, 1.46)	99.9	<0.000	12,820	0.726	0.548	Yes
	5,000–10,000	1	0.88 (0.65, 1.19)	74.5	0.048	9,965	NA	NA	NA
	>10,000	3	1.10 (0.98, 1.24)	87.9	<0.000	65,261	NA	NA	NA
Subgroup analysis by exposure measures	FFQ	2	1.31 (0.90, 1.89)	100	<0.000	6,436	NA	NA	NA
	24-h dietary recall	1	1.03 (0.98, 1.09)	27.9	0.25	15,602	NA	NA	NA
	DSQ	1	1.09 (0.91–1.30)	NR	NR	626	NA	NA	NA
	Others	5	1.04 (0.88, 1.24)	86.5	<0.000	65,382	0.886	0.536	Yes
Subgroup analysis by outcome assessment	GAD-7, SCL-90, STAI, DASS21	5	1.06 (0.87, 1.28)	99.8	<0.000	33,020	0.542	0.592	Yes
	NR	4	1.18 (0.93, 1.51)	88.2	<0.000	55,026	0.716	0.806	Yes
Subgroup analysis by quality of study	Cross-sectional								
	6	1	1.38 (1.09–1.72)	NR	NR	36,173	NA	NA	NA
	7	3	1.12 (0.83, 1.52)	90.4	<0.000	28,192	NA	NA	NA
	8	2	1.09 (0.76, 1.56)	100	<0.000	5,795	NA	NA	NA
	9	2	1.19 (0.81, 1.74)	92.2	<0.000	2,766	NA	NA	NA
	Cohort								
	8	1	1.03 (0.98, 1.09)	27.9	0.25	15,602	NA	NA	NA
Subgroup analysis by region	Asia	3	1.23 (0.91, 1.67)	90.1	<0.000	38,939	NA	NA	NA
	European	2	1.05 (0.81, 1.83)	99.9	<0.000	20,289	NA	NA	NA
	Australia	1	1.21 (0.74–1.98)	NR	NR	4,741	NA	NA	NA
	America	1	1.09 (0.93–1.28)	NR	NR	626	NA	NA	NA

NA: not available (because the number of studies is not enough to calculate).

#### 3.2.1 Subgroup analysis by study designing

Classified by study design, one cohort study showed an odds ratio (OR) of 1.03 (95% CI: 0.98, 1.09) for the association between sugar intake and anxiety risk, with *I*^2^ = 27.9%, *p* = 0.25, and involving 15,602 patients. The cross-sectional studies, comprising eight studies, had the OR = 1.11 (95% CI: 0.92, 1.34) for sugar intake and anxiety risk, with *I*^2^ = 99.7%, *p* < 0.000, and involving 72,444 patients. The results of Egger’s test (*p* = 0.651) and Begg’s test (*p* = 0.304) indicated no significant publication bias. Sensitivity analyses confirmed the robustness of the results (Table 2).

#### 3.2.2 Subgroup analysis by gender

In a subgroup analysis by gender, two studies were included in the male group, showing OR = 0.96 (95% CI: 0.77, 1.19) for the association between sugar intake and anxiety risk, with *I*^2^ = 83.6%, *p* = 0.013, and involving 2,878 patients. Only one study in the female group showed the OR = 1.36 (95% CI: 1.34, 1.36) for the association between sugar intake and anxiety risk, with 3,097 patients included. Due to the limited number of studies in this subgroup, analysis of heterogeneity and publication bias could not be performed (Table 2).

#### 3.2.3 Subgroup analysis by sample size

In subgroup analyses by sample, five studies with populations of <5,000 showed the OR = 1.14 (95% CI: 0.89, 1.46) for the association between sugar intake and anxiety risk, with *I*^2^ = 99.9%, *p* < 0.000, and including 12,820 patients. The results of Egger’s test and Begg’s test were 0.726 and 0.548, respectively, indicating that no significant publication bias was found. Sensitivity analysis showed robust results. For the 5,000–10,000 population group, which included one study, the OR for sugar intake and anxiety risk was 0.88 (95% CI: 0.65, 1.19), with *I*^2^ = 74.5%, *p* = 0.048, and 9,965 patients. Due to the single study, analysis of publication bias and sensitivity could not be performed. In the population group >10,000, three studies showed the OR = 1.10 (95% CI: 0.98, 1.24) for sugar intake and anxiety risk, with the *I*^2^ = 87.9%, *p* < 0.000, and 65,261 patients (Table 2).

#### 3.2.4 Subgroup analysis by exposure measures

In subgroup analyses categorized by exposure measurement tools, two studies using the Food Frequency Questionnaire (FFQ) showed the OR = 1.31 (95% CI: 0.90, 1.89) for the association between sugar intake and anxiety risk, with *I*^2^ = 100%, *p* < 0.000, and including 6,436 patients. As there were only two studies, analysis of publication bias and sensitivity could not be performed. In one study using a 24-h dietary review, the OR for sugar intake and anxiety risk was 1.03 (95% CI: 0.98, 1.09), with *I*^2^ = 27.9%, *p* = 0.25, and 15,602 patients. As there was only one study, analysis of publication bias and sensitivity could not be performed. One study using the Dietary Questionnaire (DSQ) showed OR = 1.09 (95% CI: 0.91, 1.30) for sugar intake and anxiety risk. Due to insufficient data, heterogeneity, publication bias, and sensitivity analyses could not be calculated, with 626 patients included. Five studies employing other exposure measurement tools showed OR = 1.04 (95% CI: 0.88, 1.24) for sugar intake and anxiety risk, with *I*^2^ 86.5%, *p* < 0.000, and 65,382 patients included. No significant publication bias was found, and sensitivity analyses showed robust results (Table 2).

#### 3.2.5 Subgroup analysis by outcome assessment

In subgroup analyses by measuring questionnaires, five studies using measuring tools such as GAD-7, SCL-90, STAI, and DASS21 showed the OR = 1.06 (95% CI: 0.87, 1.28) for the association between sugar intake and anxiety risk, with *I*^2^ = 99.8%, *p* < 0.000, and including 33,020 patients. The results of Egger’s test and Begg’s test were 0.542 and 0.592, respectively, indicating that no significant publication bias was identified. Sensitivity analysis showed robust results. In four studies that did not report specific measuring questionnaires (NRs), the OR for sugar intake and anxiety risk was 1.18 (95% CI: 0.93, 1.51), with *I*^2^ = 88.2%, *p* < 0.000, and 55,026 patients included. No significant publication bias was found, and sensitivity analysis showed robust results (Table 2).

#### 3.2.6 Subgroup analysis by quality of study

In subgroup analyses based on study quality, cross-sectional studies were categorized according to AHRQ. One study with the AHRQ score of 6 reported a combined OR of 1.38 (95% CI: 1.09, 1.72) for sugar intake and anxiety risk. Heterogeneity details were not specified, and the study included 36,173 participants. Three studies with the AHRQ score of 7 exhibited a combined OR of 1.12 (95% CI: 0.83, 1.52) for the association between sugar intake and anxiety risk. The heterogeneity (*I*^2^) was 90.4%, and the studies included 28,192 participants, with *p* < 0.000. Two studies with AHRQ score of 8 reported the OR = 1.09 (95% CI: 0.76, 1.56) for sugar intake and anxiety risk. The heterogeneity (*I*^2^) was 100%, with *p* < 0.000, and the studies included 5,795 participants. Two studies with AHRQ score of 9 reported the OR = 1.19 (95% CI: 0.81, 1.74) for sugar intake and anxiety risk. The heterogeneity (*I*^2^) was 92.2% (*I*^2^), with *p* < 0.000, and the studies included 2,766 participants. Among the cohort studies, one with a NOS score of 8 reported an OR = 1.03 (95% CI: 0.98, 1.09) for the association between sugar intake and anxiety risk. The heterogeneity (*I*^2^) was 27.9%, with *p* = 0.25, and the study included 15,602 participants (Table 2).

#### 3.2.7 Subgroup analysis by region

In a subgroup analysis by region, three studies conducted in Asia reported the OR = 1.23 (95% CI: 0.91, 1.67) for the association between sugar intake and anxiety risk. The heterogeneity (*I*^2^) was 90.1%, with *p* < 0.000, and the studies included 38,939 participants. Due to the limited number of studies within each regional group, analysis of publication bias and sensitivity could not be performed. Two studies conducted in Europe reported the OR = 1.05 (95% CI: 0.81, 1.83) for the association between sugar intake and anxiety risk. The heterogeneity (*I*^2^) was 99.9%, with *p* < 0.000, and the studies included 20,289 participants. One study conducted in Australia reported the OR = 1.21 (95% CI: 0.74, 1.98) for the association between sugar intake and anxiety risk. Specific heterogeneity was not reported, and the study included 4,741 participants. One study conducted in the Americas reported the OR = 1.09 (95% CI: 0.93, 1.28) for the association between sugar intake and anxiety risk. Specific heterogeneity was not reported, and the study included 626 participants (Table 2).

Overall, a significant association between sugar intake and depression risk was observed in the overall analysis, in groups with sample size exceeding 10,000, in studies utilizing Food Frequency Questionnaires (FFQs) and the self-made questionnaires, as well as in studies employing PHQ-9, CES-D, and other measuring questionnaires. These findings indicate that sugar intake may significantly increase the risk of depression. In meta-analyses examining the association between sugar intake and anxiety risk, while the overall results did not reach statistical significance, some subgroups approached significance, particularly studies with smaller samples and those employing FFQs. Nevertheless, additional high-quality studies are required to further validate this association and enhance the reliability of the findings related to anxiety risk. An overall review of our analyses indicated that while significant associations were observed between sugar intake and depression risk, the high level of heterogeneity suggests a need for additional high-quality studies to validate these associations and investigate the underlying mechanisms. Furthermore, sensitivity analyses and the assessment of publication bias suggest that most of the results are robust.

## 4 Discussion

This study systematically assessed the association between sugar intake and the risk of depression and anxiety through a comprehensive systematic review and meta-analysis. The analysis revealed a significant increase in the risk of depression associated with sugar intake, while the effect on anxiety risk was not statistically significant. The study evaluated this association across various study designs (e.g., cross-sectional, cohort, case–control) and explored the moderating effects of different population characteristics (e.g., gender, age, region) to identify potentially susceptible groups. Additionally, it assessed the impact of different methods of measuring exposure (e.g., Food Frequency Questionnaires, 24-h dietary recalls, and self-reported questionnaires) on the study results to ensure data accuracy and consistency. The study also identified and interpreted heterogeneity and publication bias to ensure the robustness and reliability of the findings. Multiple subgroup analyses (e.g., study design, gender, sample, exposure measures, outcome assessment, study quality, region) were conducted to further investigate the association between total sugar intake and depression and anxiety disorders, providing detailed and accurate evidence.

The significant association between sugar intake and the risk of depression may be attributed to several mechanisms. Firstly, diets high in sugar induce sharp fluctuations in blood sugar levels, leading to mood swings and anxiety. Rapid fluctuations in blood sugar activate the stress response and increase cortisol secretion, affecting mood stability (61). Additionally, sugar impairs the function of the hypothalamic–pituitary–adrenal (HPA) axis, a crucial component of the neuroendocrine system. This impairment can lead to stress and metabolic disorders, such as obesity and diabetes, contributing to oxidative stress and inflammation (62). Prolonged sugar intake may also trigger systemic and neurological inflammatory responses (63). Elevated inflammatory factors, such as TNF-*α* and IL-6, are strongly associated with the development of depression. Chronic inflammation can disrupt neuroplasticity in the brain, potentially leading to depressive symptoms (64). Moreover, the brain–gut axis theory suggests that a high-sugar diet alters the composition of the gut microbiota, reducing beneficial bacteria and increasing harmful bacteria. Studies on the gut–brain axis have shown that gut microbiota dysbiosis can influence brain function and mood regulation through the release of inflammatory factors and metabolites (65). Additionally, sugar intake may lower levels of brain-derived neurotrophic factor (BDNF), essential for neuroplasticity and emotional regulation. A decrease in BDNF levels is associated with the development of depression (66). Disruptions in the dopamine system are also considered underlying mechanisms of depression and anxiety (67). Long-term high-sugar intake may affect the brain’s dopamine system, altering reward mechanisms and pleasurable sensations.

The association between sugar intake and anxiety was not statistically significant, which may be due to a variety of reasons. From the perspective of the mechanism of sugar on anxiety, on the one hand, sugar intake may participate in the neuroinflammatory process in brain regions by changing the levels of inflammatory factors such as IL-6, TNF-*α*, leptin, and iNO, leading to excessive production of nitric oxide. This induces anxiety-like behaviors (68, 69). On the other hand, high-sugar intake promotes dopamine release, which enhances pleasure and comfort (1). From the perspective of the type of included studies, most of the included studies were cross-sectional studies, and cross-sectional studies were not as rigorous as cohort studies or randomized clinical trials in study design, which may also lead to inaccurate results. In the future, more well-designed studies are needed to further explore the association between sugar intake and anxiety.

The high heterogeneity observed in this study indicates considerable variability among the included studies. Subgroup analyses revealed that variations in study design, gender, sample characteristics, exposure measurement tools, and outcome assessment significantly influenced the results.

When using different exposure measurement tools in the analysis of sugar intake and depression, we found high heterogeneity (*I*^2^ = 99.7%) among the nine studies using the FFQ, possibly due to unclear definitions of snacks in 24-h dietary records and frequent underreporting of certain food groups (e.g., sauces and condiments). Some studies also record regional foods (such as Korean sweet rice punches), so future studies should use more objective dietary recording methods to fully capture actual intake.

Different measuring tools were used in the studies when assessing depressive and anxiety symptoms, which has the potential to lead to inconsistent conclusions. The BDI is suitable for adults at different stages of depression, while the SDS is more suitable for adolescents over 12 years old. The PHQ-9 scales focus on assessing the severity of depressive conditions, whereas the CES-D scales focus on assessing the frequency of current depressive symptoms. The GAD-7 scale is suitable for the screening of anxiety in the general population and the assessment of treatment results, while the SCL-90 scale is suitable for the self-detection of patients, but not suitable for the screening of healthy people. Therefore, the use of different measuring tools in the assessment of symptoms is likely to lead to a large heterogeneity in the results. It is hoped that this interference can be eliminated by unifying measurement standards in the future.

In the subgroup analysis of literature quality evaluation, we used the quality assessment of Newcastle-Ottawa Scale (NOS) and AHRQ tool. Although high-quality literature can provide more accurate results, literature with medium quality (NOS score of 6–7) accounted for the majority of the included literature. These moderate-quality studies may have led to bias by not identifying the expected percentage of patients with incomplete data or follow-up outcomes or by not explaining the reasons for excluding patients from the analyses. For these studies, we used a sensitivity analysis to assess its effect.

Sensitivity and funnel plot analyses demonstrated that most results were robust. Although some publication bias was detected, additional high-quality longitudinal studies are necessary to further validate this association and explore causality. Future research should focus on: (1) Designing longitudinal studies with extended follow-up periods and genome-wide association studies to elucidate the causal relationship between sugar intake and the risk of depression and anxiety; (2) Comparing the relationship between sugar intake and depression and anxiety in different populations can help to provide ideas for disease prevention in specific populations (such as people with diabetes). (3) Comparative studies evaluating the effects of various dietary patterns (e.g., Eastern Mediterranean diet, vegetarian diet) on mental health, to further elucidate the protective effects of a healthy diet. (4) Investigations into the impact of cultural and regional differences on the relationship between sugar intake and mental health, including cross-cultural and cross-regional comparative studies. (5) In addition, some modifiable lifestyle behaviors, such as exercise and sleep, may also have a significant impact on depression and anxiety. Studies have shown that increased physical activity can alleviate sugar-induced depressive symptoms by reducing inflammation and improving sleep quality (70). Given the complex influence of these factors on mental health, the findings of this study may be limited by the lack of consideration of these lifestyle variables. Therefore, future studies should further explore the interplay between exercise, sleep, and sugar-related depressive symptoms.

## 5 Conclusion

Amid ongoing research into the relationship between sugar intake and mental health risks, this systematic review and meta-analysis identified a significant association between sugar intake and the risk of depression, while the impact on anxiety risk remains inconclusive and warrants further investigation. These findings highlight the need to improve dietary habits. We hope that our research will provide new insights and strategies for the prevention and management of depression and anxiety disorders, thereby advancing public health. Our study aims to raise public awareness about the potential risks of high-sugar diets and to promote the adoption of healthier eating habits, which could offer substantial health benefits for both individuals and society. Despite the heterogeneity and potential biases present in the current studies, the overall results suggest a significant effect of sugar intake on depression risk. Future research should focus on high-quality studies to further explore this association and its underlying mechanisms.

## Data Availability

The original contributions presented in the study are included in the article/Supplementary material, further inquiries can be directed to the corresponding author/s.
