# Supplementary material for: Association of sugar consumption with risk of depression and anxiety: a systematic review and meta-analysis
Source: Front Nutr. 2024 Oct 16;11:1472612. doi: 10.3389/fnut.2024.1472612 (PMC11522855; doi:10.3389/fnut.2024.1472612)
Supplement: Supplementary file 1 [file Image_1.pdf]

Supplementary Material

1 Forest plot of the relationship between sugar intake and depression

1.1 subgroup analysis

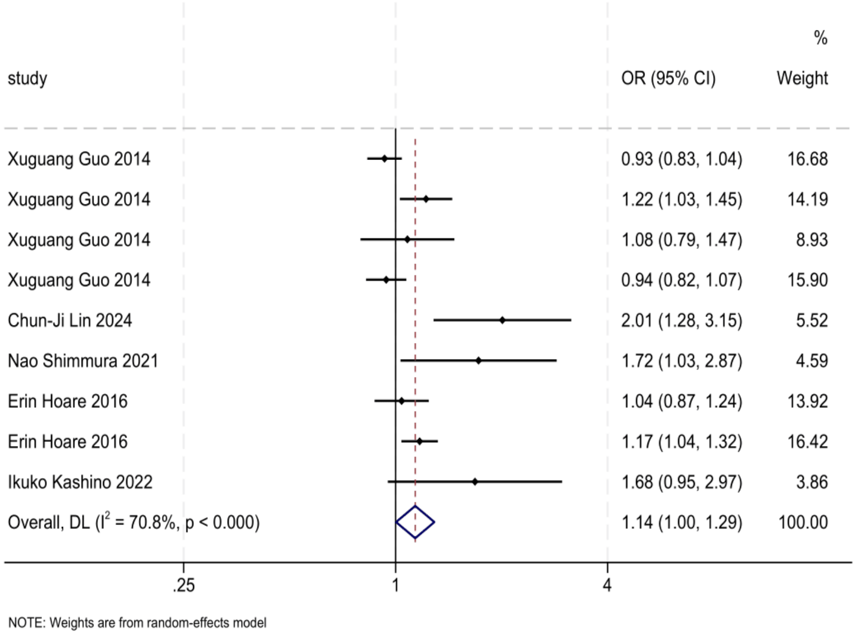

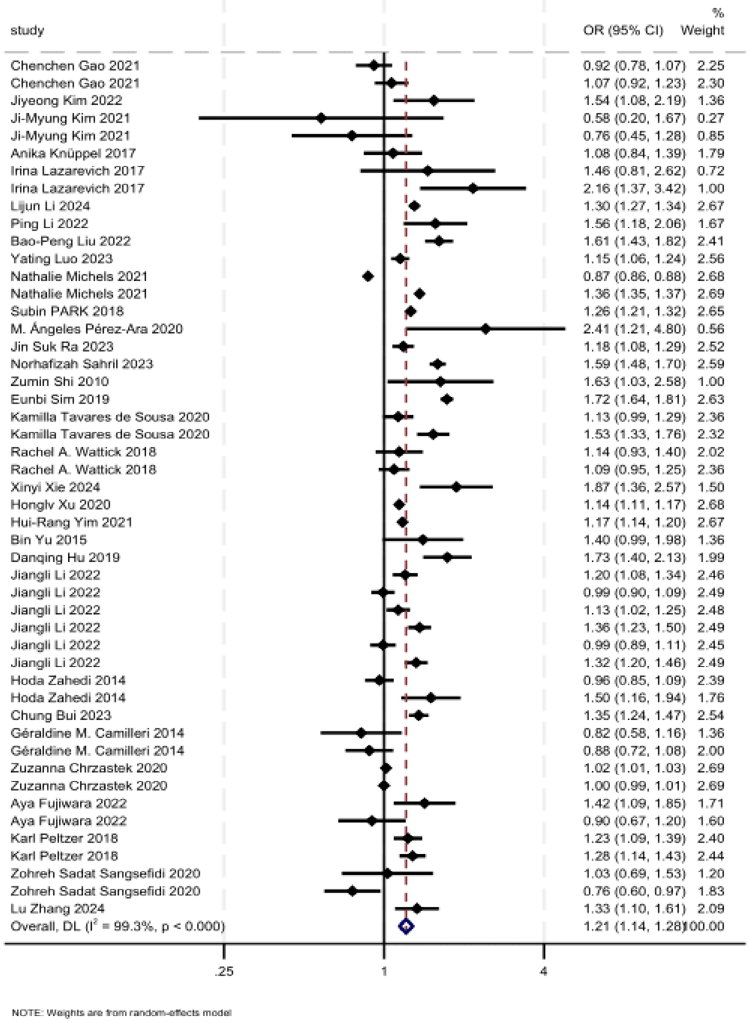

Supplementary Figure 1.1.1 Forest plot of (A) Study Designing (cohort); (B) Study Designing (cross-sectional).

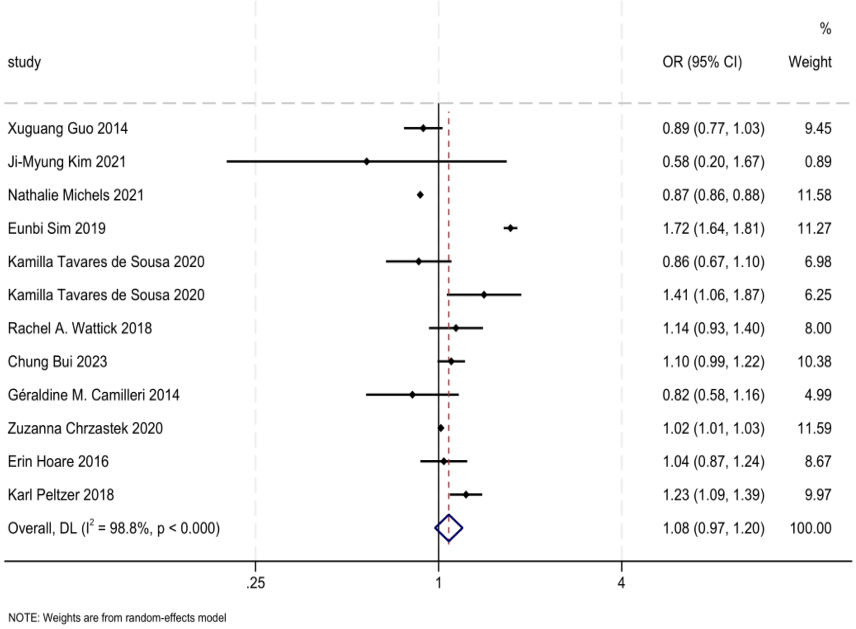

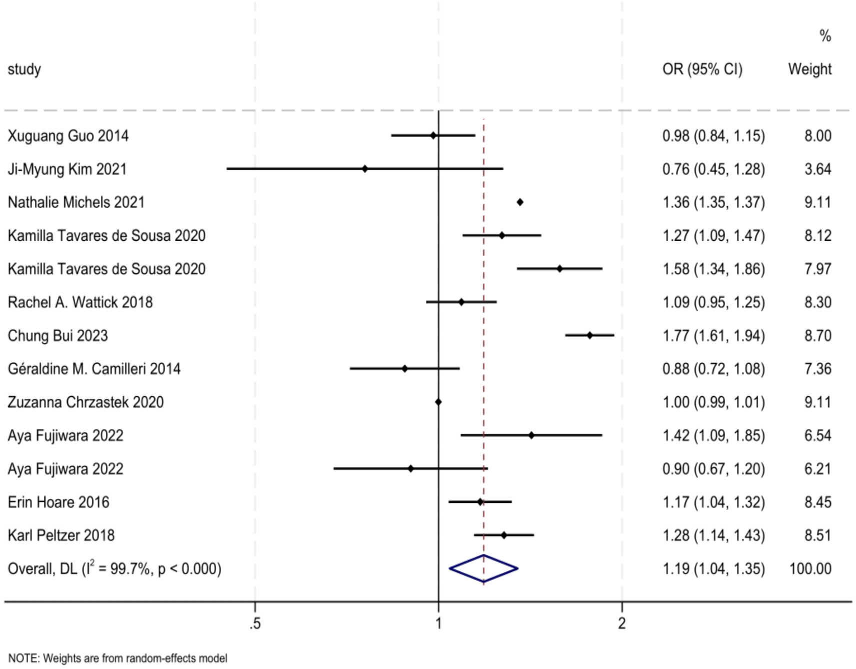

Supplementary Figure 2.1.2 Forest plot of (A) Sex (male); (B) Sex (female).

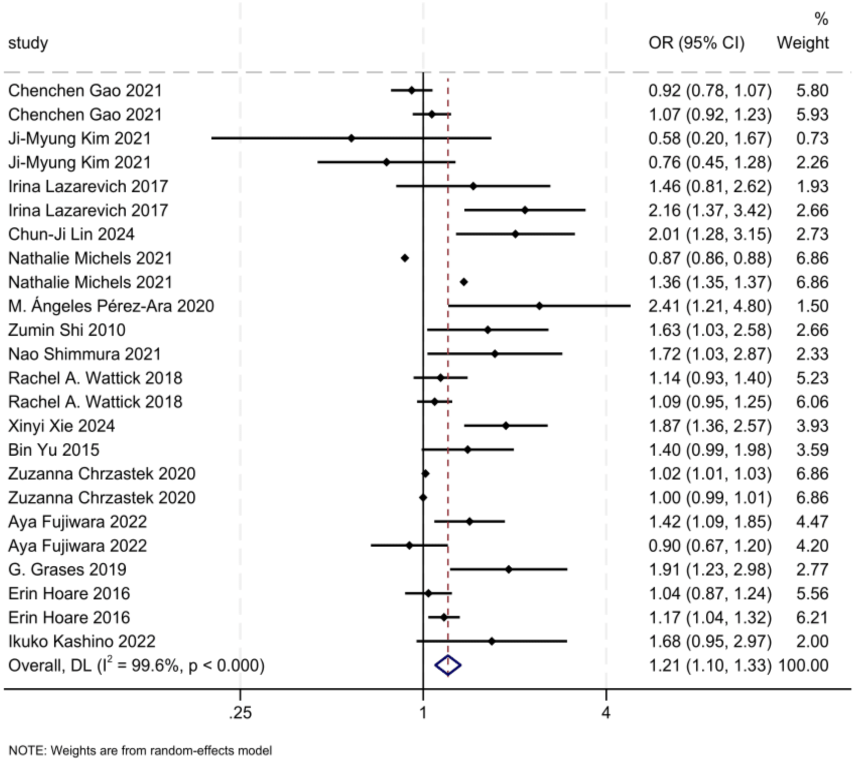

study
OR (95% CI)
%
Weight
Chenchen Gao 2021 0.92 (0.78, 1.07) 5.80
Chenchen Gao 2021 1.07 (0.92, 1.23) 5.93
Ji-Myung Kim 2021 0.58 (0.20, 1.67) 0.73
Ji-Myung Kim 2021 0.76 (0.45, 1.28) 2.26
Irina Lazarevich 2017 1.46 (0.81, 2.62) 1.93
Irina Lazarevich 2017 2.16 (1.37, 3.42) 2.66
Chun-Ji Lin 2024 2.01 (1.28, 3.15) 2.73
Nathalie Michels 2021 0.87 (0.86, 0.88) 6.86
Nathalie Michels 2021 1.36 (1.35, 1.37) 6.86
M. Ángeles Pérez-Ara 2020 2.41 (1.21, 4.80) 1.50
Zumin Shi 2010 1.63 (1.03, 2.58) 2.66
Nao Shimmura 2021 1.72 (1.03, 2.87) 2.33
Rachel A. Wattick 2018 1.14 (0.93, 1.40) 5.23
Rachel A. Wattick 2018 1.09 (0.95, 1.25) 6.06
Xinyi Xie 2024 1.87 (1.36, 2.57) 3.93
Bin Yu 2015 1.40 (0.99, 1.98) 3.59
Zuzanna Chrzastek 2020 1.02 (1.01, 1.03) 6.86
Zuzanna Chrzastek 2020 1.00 (0.99, 1.01) 6.86
Aya Fujiwara 2022 1.42 (1.09, 1.85) 4.47
Aya Fujiwara 2022 0.90 (0.67, 1.20) 4.20
G. Grases 2019 1.91 (1.23, 2.98) 2.77
Erin Hoare 2016 1.04 (0.87, 1.24) 5.56
Erin Hoare 2016 1.17 (1.04, 1.32) 6.21
Ikuko Kashino 2022 1.68 (0.95, 2.97) 2.00
Overall, DL (I2 = 99.6%, p < 0.000) 1.21 (1.10, 1.33) 100.00
.25
1
4
NOTE: Weights are from random-effects model

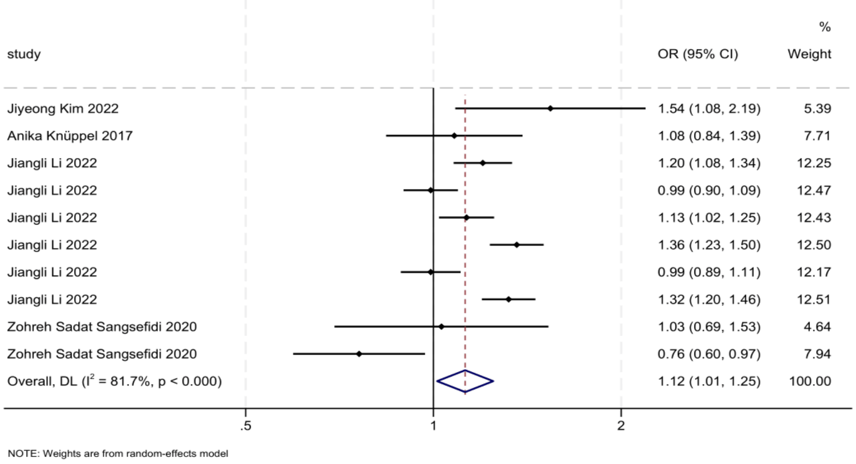

study
OR (95% CI)
%
Weight
Jiyeong Kim 2022 1.54 (1.08, 2.19) 5.39
Anika Knüppel 2017 1.08 (0.84, 1.39) 7.71
Jiangli Li 2022 1.20 (1.08, 1.34) 12.25
Jiangli Li 2022 0.99 (0.90, 1.09) 12.47
Jiangli Li 2022 1.13 (1.02, 1.25) 12.43
Jiangli Li 2022 1.36 (1.23, 1.50) 12.50
Jiangli Li 2022 0.99 (0.89, 1.11) 12.17
Jiangli Li 2022 1.32 (1.20, 1.46) 12.51
Zohreh Sadat Sangsefidi 2020 1.03 (0.69, 1.53) 4.64
Zohreh Sadat Sangsefidi 2020 0.76 (0.60, 0.97) 7.94
Overall, DL (I2 = 81.7%, p < 0.000) 1.12 (1.01, 1.25) 100.00
.5
1
2
NOTE: Weights are from random-effects model

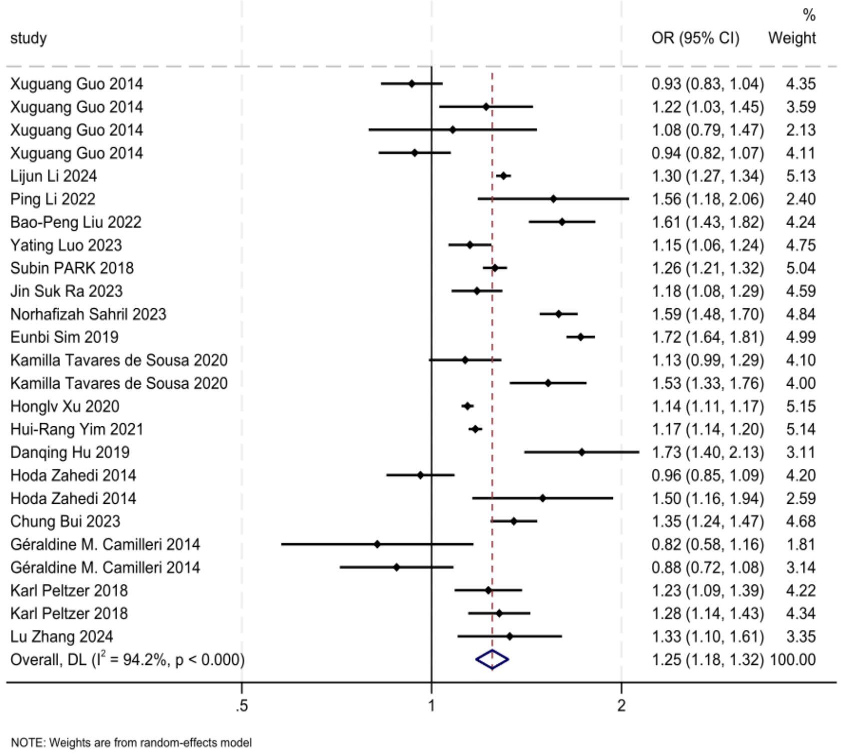

Supplementary Figure 3.1.3 Forest plot of (A) Sample Size (＜5000); (B) Sample Size (5000-10000); (C) Sample Size (＞10000).

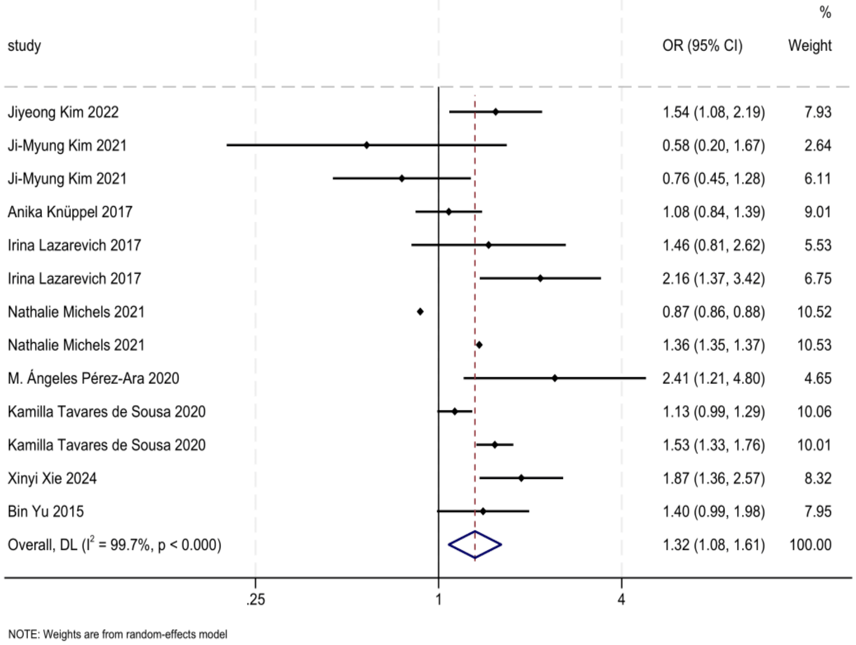

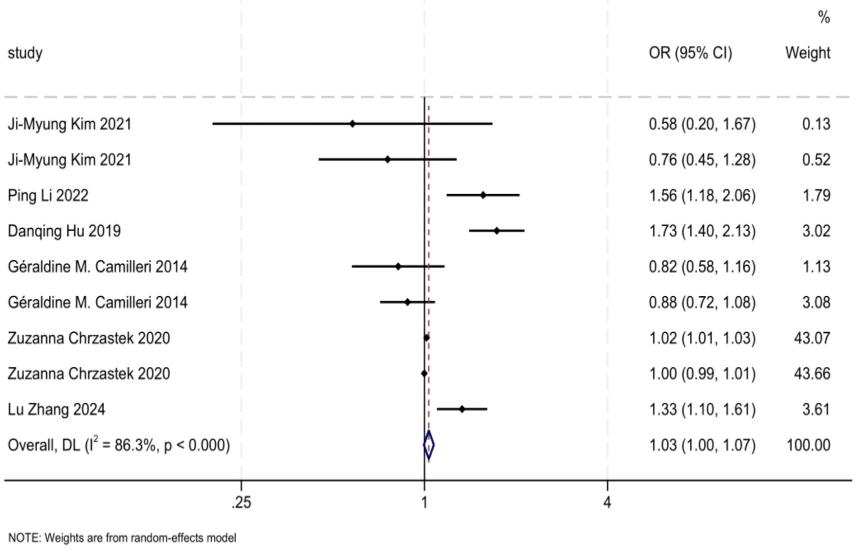

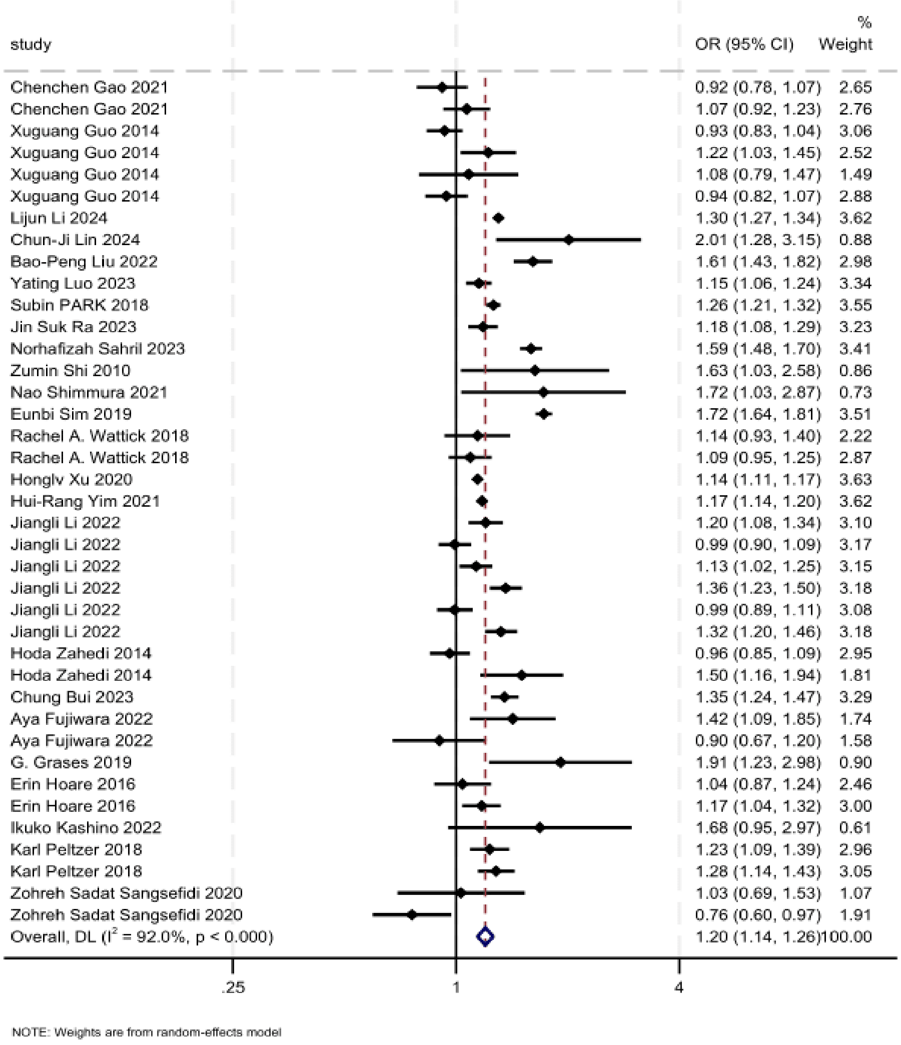

Supplementary Figure 4.1.4 Forest plot of (A) Exposure Measures (FFQ); (B) Exposure Measures

(24-h dietary recall); (C) Exposure Measures (self-made questionnaire).

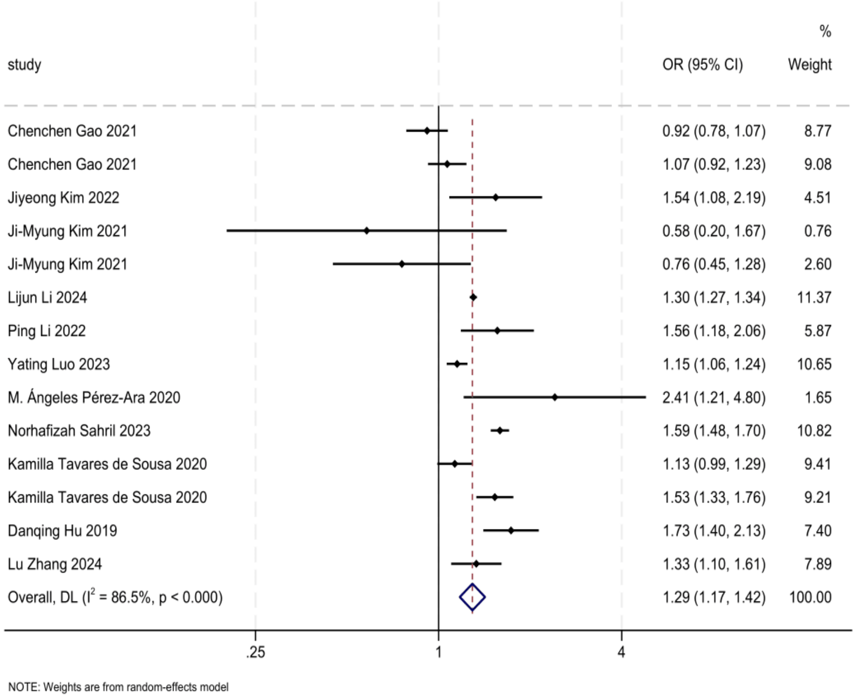

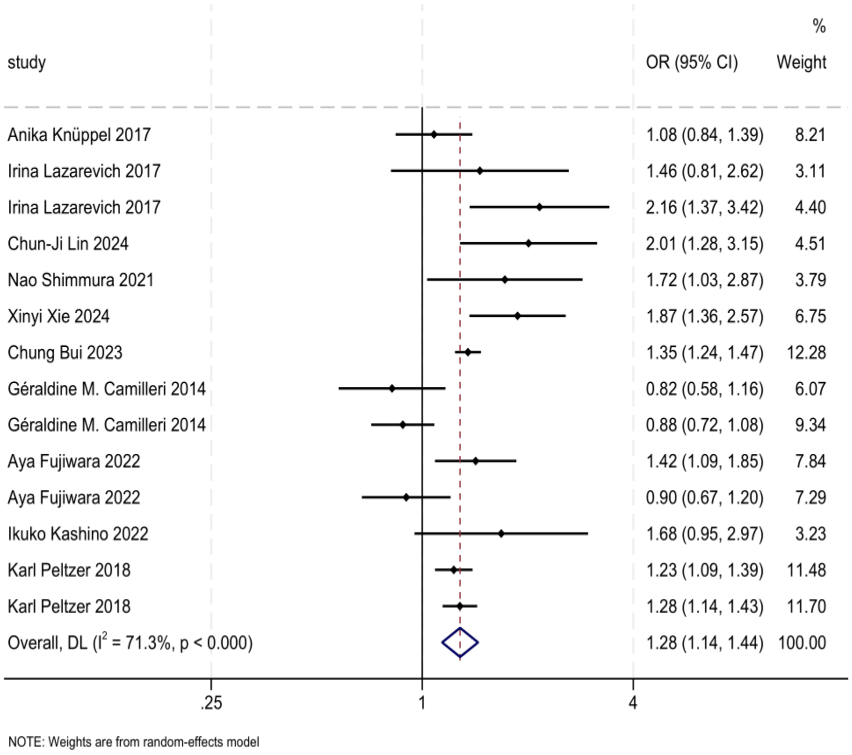

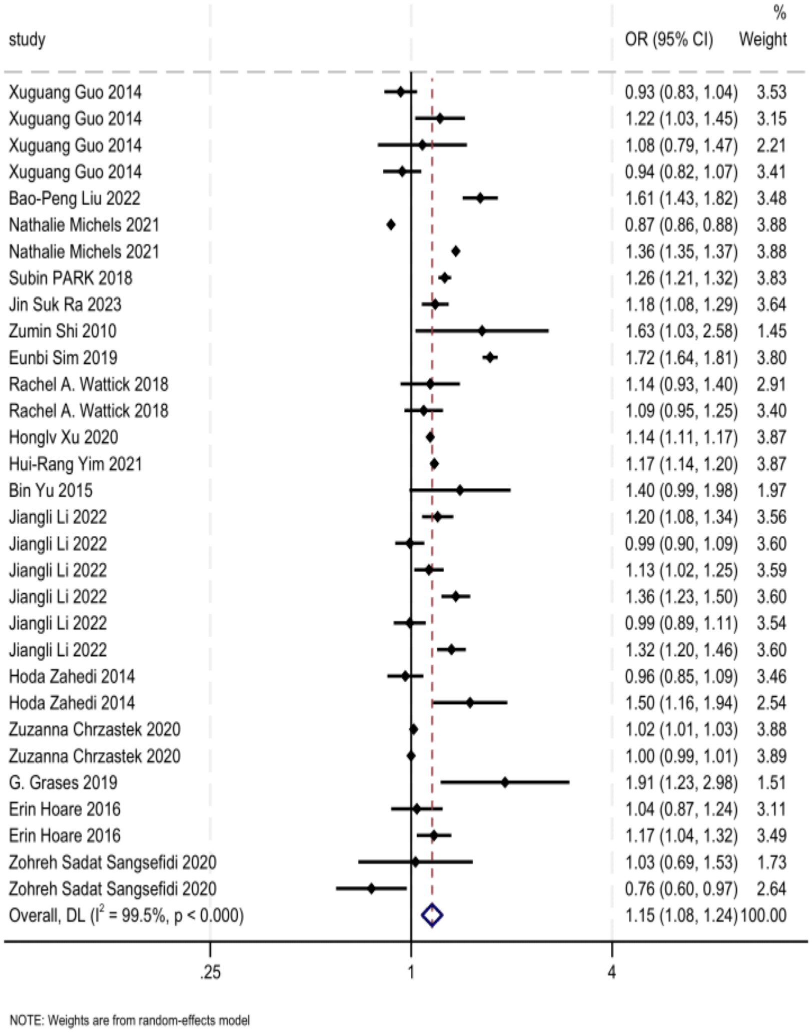

Supplementary Figure 5.1.5 Forest plot of (A) Outcome Assessment (PHQ-9); (B) Outcome Assessment (CES); (C) Outcome Assessment (others).

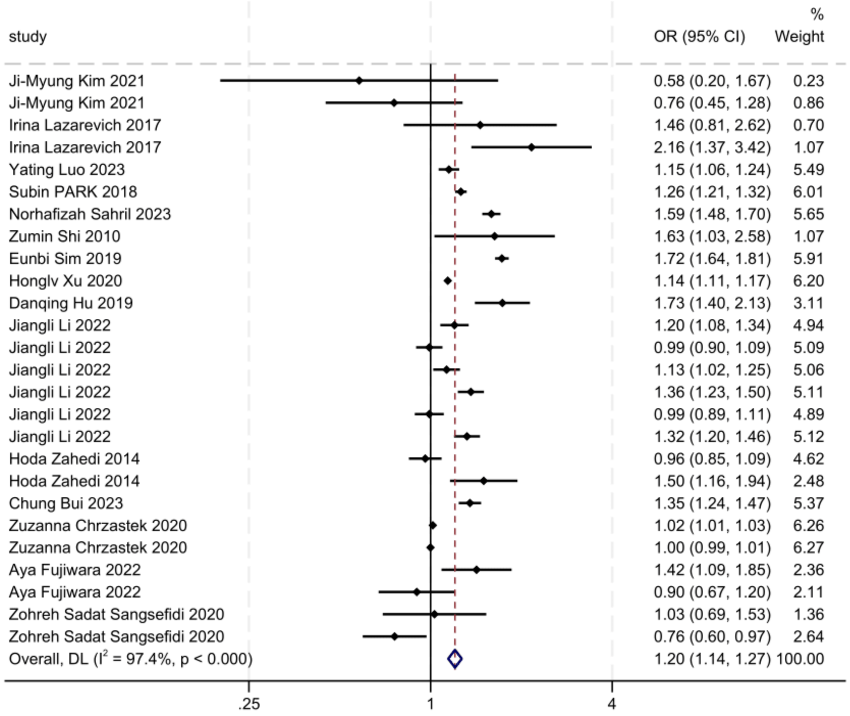

NOTE: Weights are from random-effects model

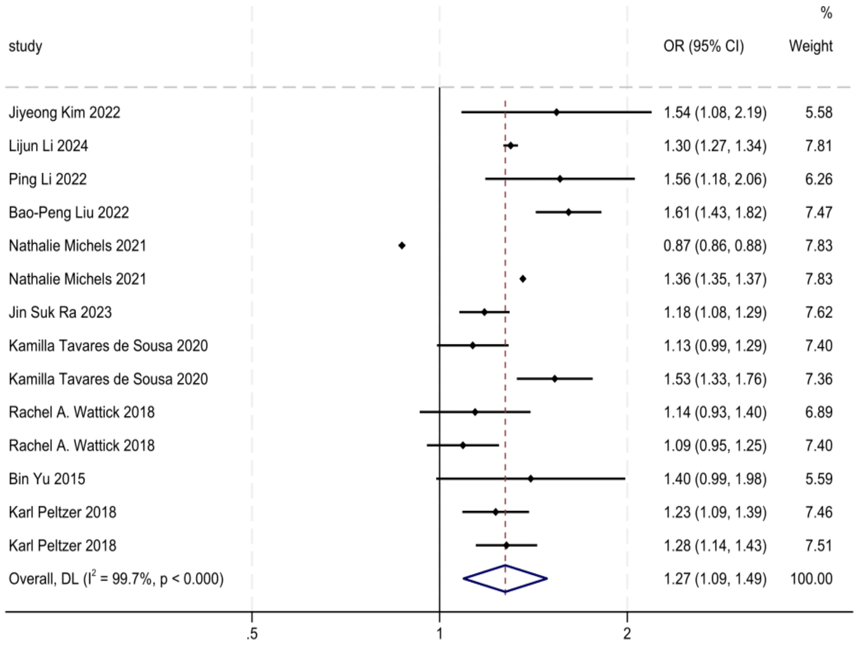

NOTE: Weights are from random-effects model

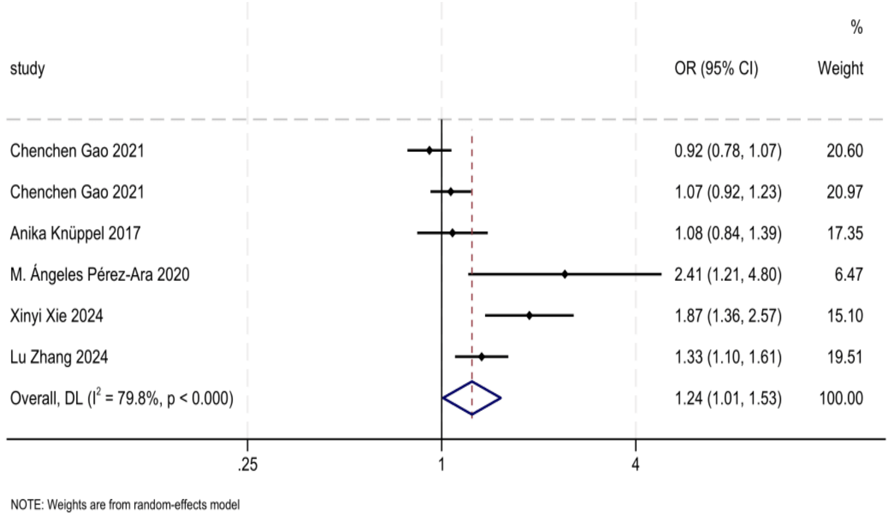

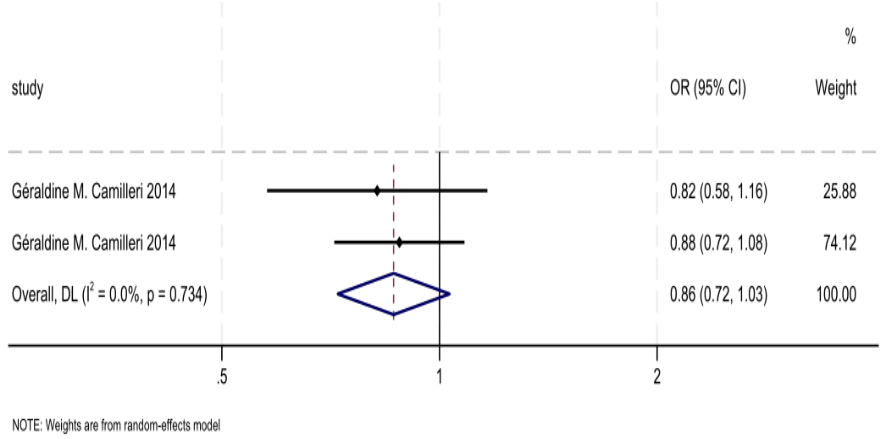

Supplementary Figure 6.1.6 Forest plot of (A) Quality Scores of Cross-Sectional Study (7); (B) Quality Scores of Cross-Sectional Study (8); (C) Quality Scores of Cross-Sectional Study (9); (D) Quality Scores of Cross-Sectional Study (10).

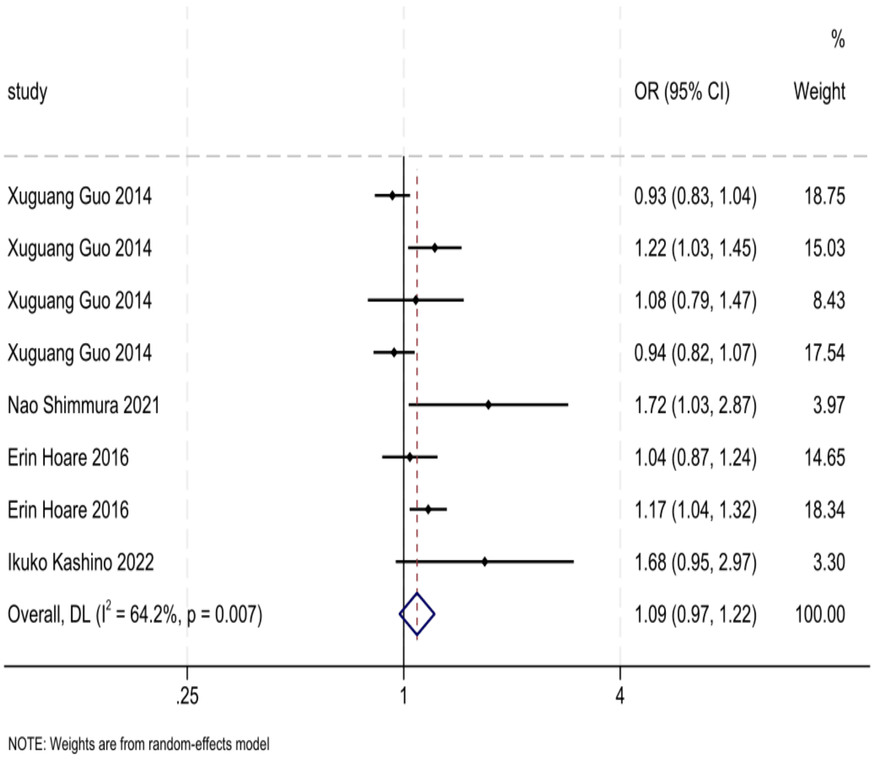

Supplementary Figure 7.1.7 Forest plot of (A) Quality Scores of Cohort Study (7).

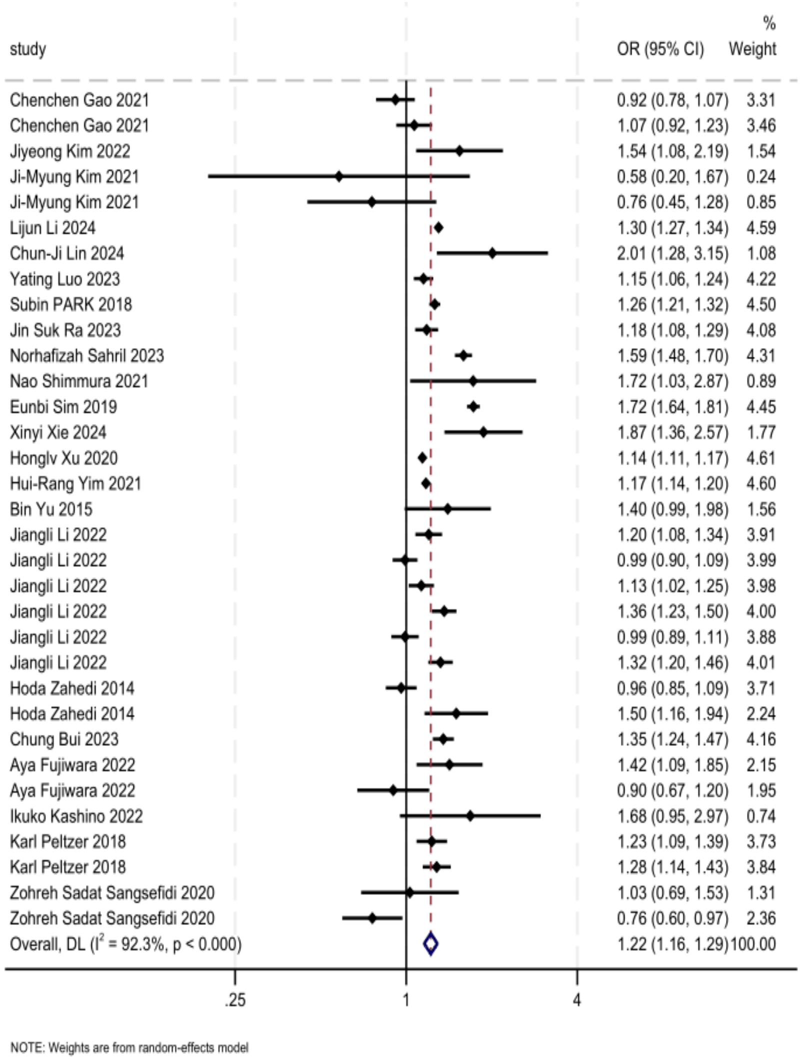
study
OR (95% CI)
% Weight
Chenchen Gao 2021 0.92 (0.78, 1.07) 3.31
Chenchen Gao 2021 1.07 (0.92, 1.23) 3.46
Jiyeong Kim 2022 1.54 (1.08, 2.19) 1.54
Ji-Myung Kim 2021 0.58 (0.20, 1.67) 0.24
Ji-Myung Kim 2021 0.76 (0.45, 1.28) 0.85
Lijun Li 2024 1.30 (1.27, 1.34) 4.59
Chun-Ji Lin 2024 2.01 (1.28, 3.15) 1.08
Yating Luo 2023 1.15 (1.06, 1.24) 4.22
Subin PARK 2018 1.26 (1.21, 1.32) 4.50
Jin Suk Ra 2023 1.18 (1.08, 1.29) 4.08
Norhafizah Sahril 2023 1.59 (1.48, 1.70) 4.31
Nao Shimmura 2021 1.72 (1.03, 2.87) 0.89
Eunbi Sim 2019 1.72 (1.64, 1.81) 4.45
Xinyi Xie 2024 1.87 (1.36, 2.57) 1.77
Honglv Xu 2020 1.14 (1.11, 1.17) 4.61
Hui-Rang Yim 2021 1.17 (1.14, 1.20) 4.60
Bin Yu 2015 1.40 (0.99, 1.98) 1.56
Jiangli Li 2022 1.20 (1.08, 1.34) 3.91
Jiangli Li 2022 0.99 (0.90, 1.09) 3.99
Jiangli Li 2022 1.13 (1.02, 1.25) 3.98
Jiangli Li 2022 1.36 (1.23, 1.50) 4.00
Jiangli Li 2022 0.99 (0.89, 1.11) 3.88
Jiangli Li 2022 1.32 (1.20, 1.46) 4.01
Hoda Zahedi 2014 0.96 (0.85, 1.09) 3.71
Hoda Zahedi 2014 1.50 (1.16, 1.94) 2.24
Chung Bui 2023 1.35 (1.24, 1.47) 4.16
Aya Fujiwara 2022 1.42 (1.09, 1.85) 2.15
Aya Fujiwara 2022 0.90 (0.67, 1.20) 1.95
Ikuko Kashino 2022 1.68 (0.95, 2.97) 0.74
Karl Peltzer 2018 1.23 (1.09, 1.39) 3.73
Karl Peltzer 2018 1.28 (1.14, 1.43) 3.84
Zohreh Sadat Sangsefidi 2020 1.03 (0.69, 1.53) 1.31
Zohreh Sadat Sangsefidi 2020 0.76 (0.60, 0.97) 2.36
Overall, DL (I^2 = 92.3%, p < 0.000) 1.22 (1.16, 1.29) 100.00
.25
1
4
NOTE: Weights are from random-effects model

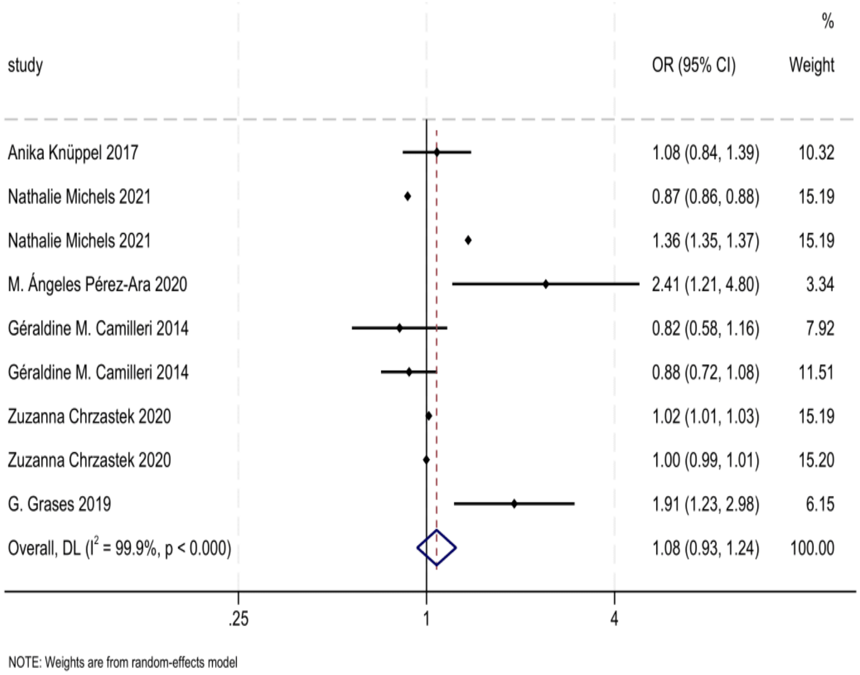
%
study
OR (95% CI)
Weight
Anika Knüppel 2017
1.08 (0.84, 1.39)
10.32
Nathalie Michels 2021
0.87 (0.86, 0.88)
15.19
Nathalie Michels 2021
1.36 (1.35, 1.37)
15.19
M. Ángeles Pérez-Ara 2020
2.41 (1.21, 4.80)
3.34
Géraldine M. Camilleri 2014
0.82 (0.58, 1.16)
7.92
Géraldine M. Camilleri 2014
0.88 (0.72, 1.08)
11.51
Zuzanna Chrzastek 2020
1.02 (1.01, 1.03)
15.19
Zuzanna Chrzastek 2020
1.00 (0.99, 1.01)
15.20
G. Grases 2019
1.91 (1.23, 2.98)
6.15
Overall, DL (I² = 99.9%, p < 0.000)
1.08 (0.93, 1.24)
100.00
.25
1
4
NOTE: Weights are from random-effects model

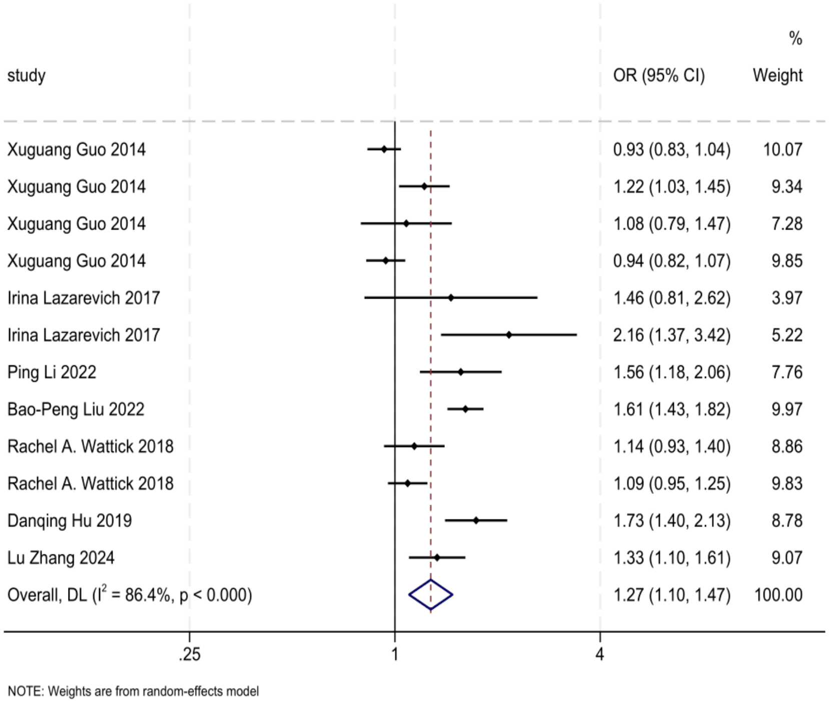
%
study
OR (95% CI)
Weight
Xuguang Guo 2014
0.93 (0.83, 1.04)
10.07
Xuguang Guo 2014
1.22 (1.03, 1.45)
9.34
Xuguang Guo 2014
1.08 (0.79, 1.47)
7.28
Xuguang Guo 2014
0.94 (0.82, 1.07)
9.85
Irina Lazarevich 2017
1.46 (0.81, 2.62)
3.97
Irina Lazarevich 2017
2.16 (1.37, 3.42)
5.22
Ping Li 2022
1.56 (1.18, 2.06)
7.76
Bao-Peng Liu 2022
1.61 (1.43, 1.82)
9.97
Rachel A. Wattick 2018
1.14 (0.93, 1.40)
8.86
Rachel A. Wattick 2018
1.09 (0.95, 1.25)
9.83
Danqing Hu 2019
1.73 (1.40, 2.13)
8.78
Lu Zhang 2024
1.33 (1.10, 1.61)
9.07
Overall, DL (I² = 86.4%, p < 0.000)
1.27 (1.10, 1.47)
100.00
.25
1
4
NOTE: Weights are from random-effects model

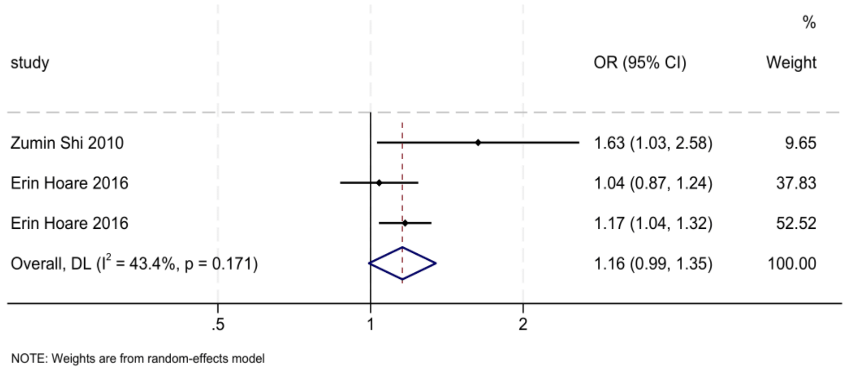

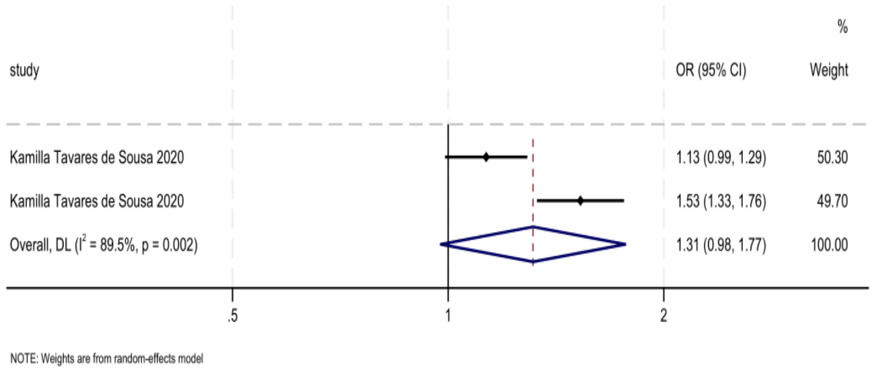

Supplementary Figure 8.1.8 Forest plot of (A) Region (Asia); (B) Region (European); (C) Region (North America); (D) Region (Australia); (E) Region (South America).

2 Forest plot of the relationship between sugar intake and anxiety

2.1 subgroup analysis

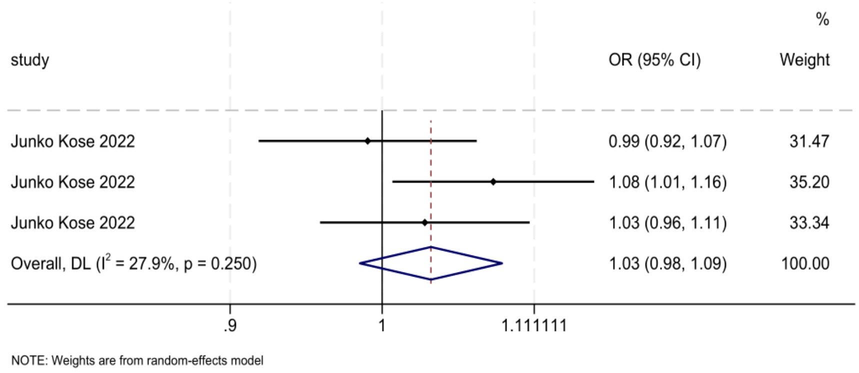

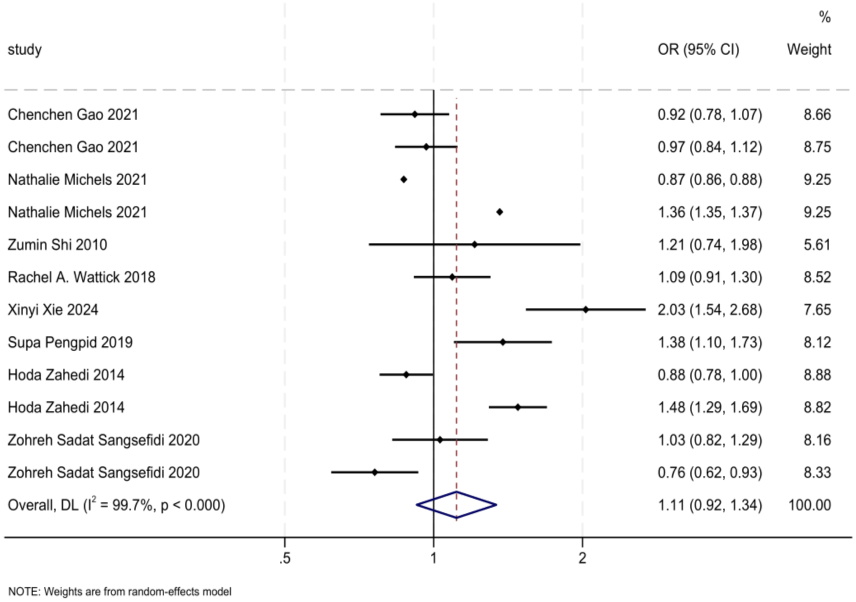

Supplementary Figure 2.1.1 Forest plot of (A) Study Designing (cohort); (B) Study Designing (cross-sectional).

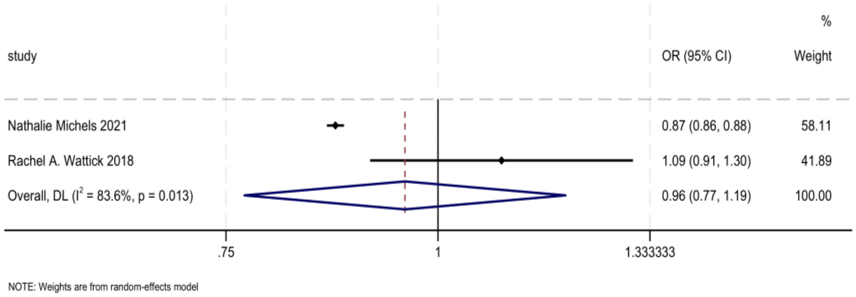

Supplementary Figure 2.1.2 Forest plot of (A) Sex (male).

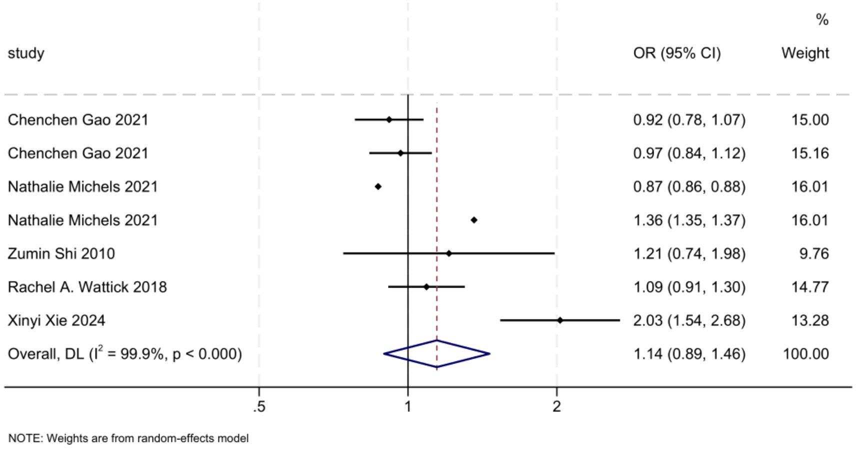

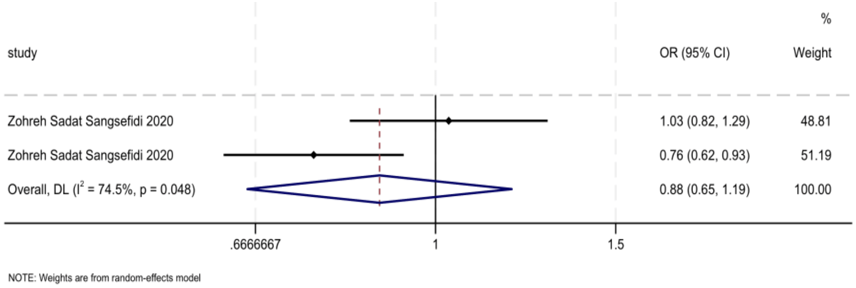

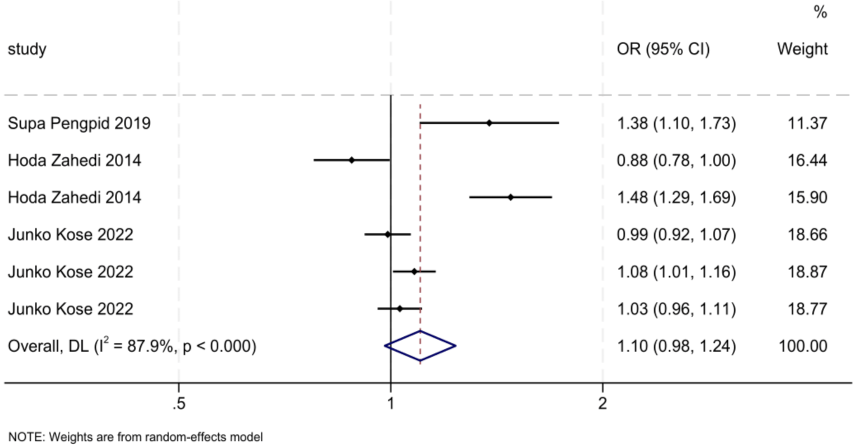

Supplementary Figure 2.1.3 Forest plot of (A) Sample Size (＜5000); (B) Sample Size (5000-10000); (C) Sample Size (＞10000).

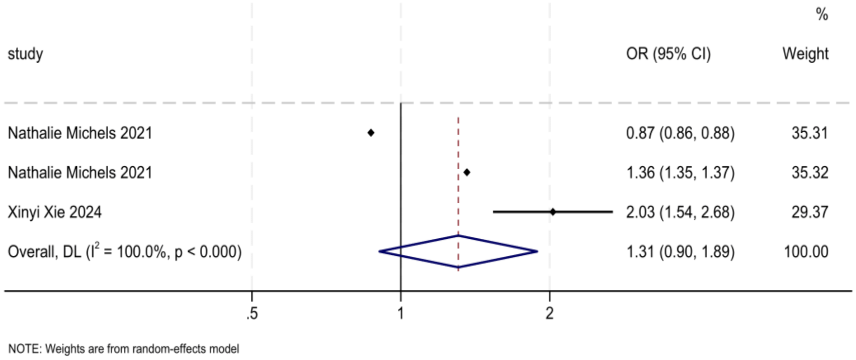

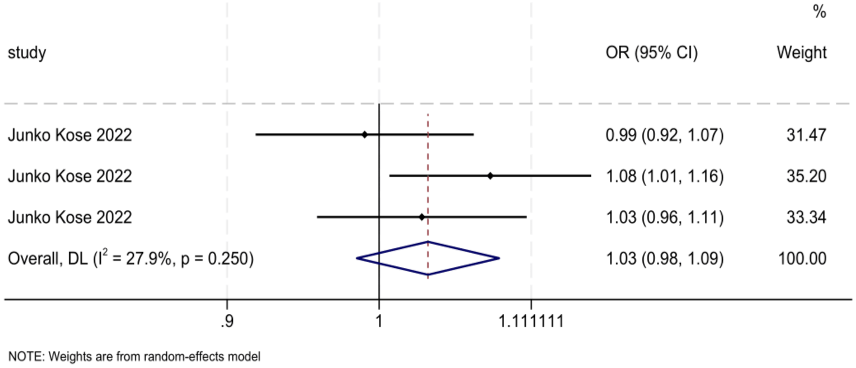

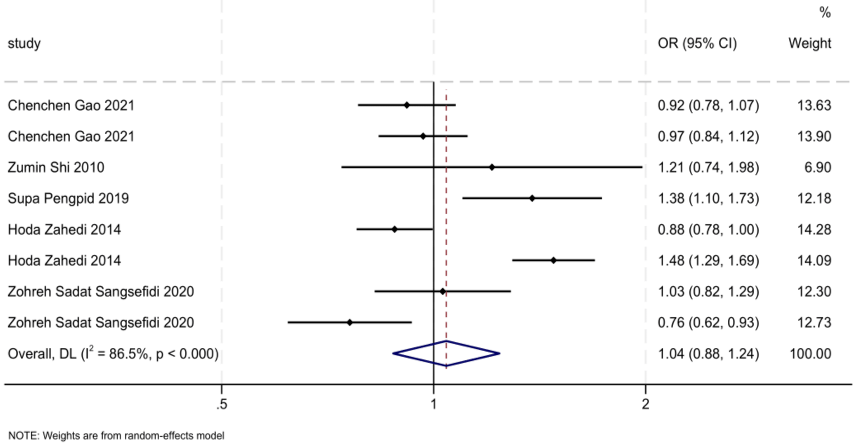

Supplementary Figure 2.1.4 Forest plot of (A) Exposure Measures (FFQ); (B) Exposure Measures (24-h dietary recall); (C) Exposure Measures (others).

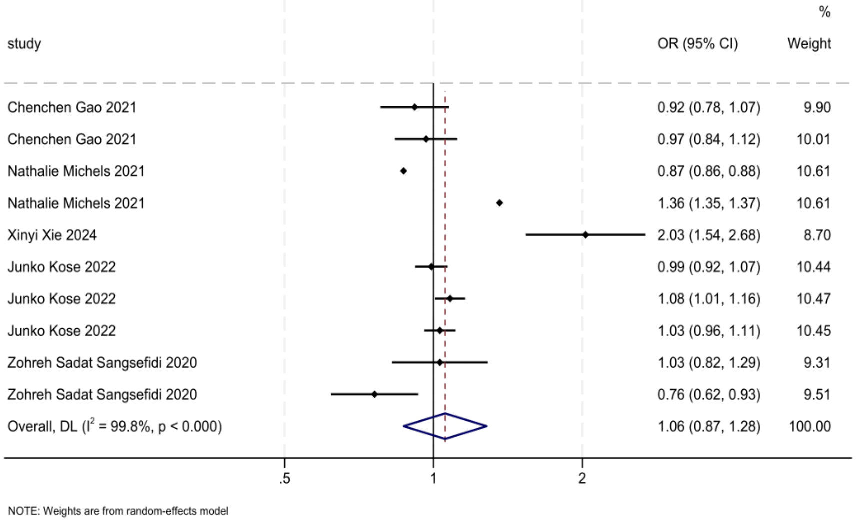

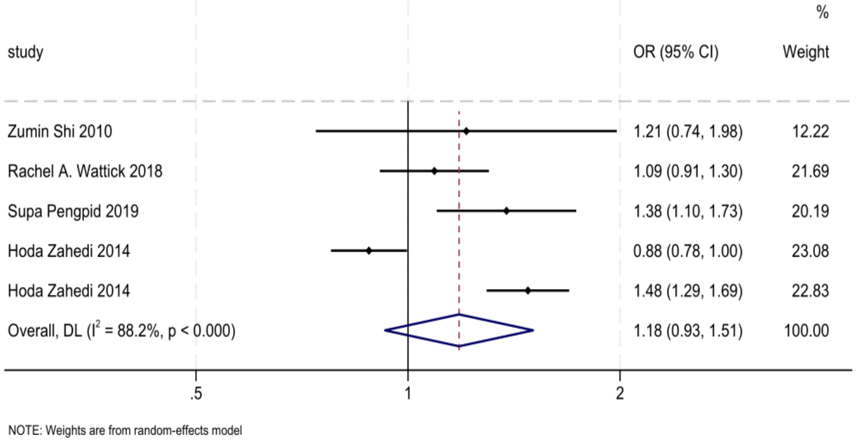

Supplementary Figure 2.1.5 Forest plot of (A) Outcome Assessment (GAD-7, SCL-90, STAI, DASS21); (B) Outcome Assessment (others).

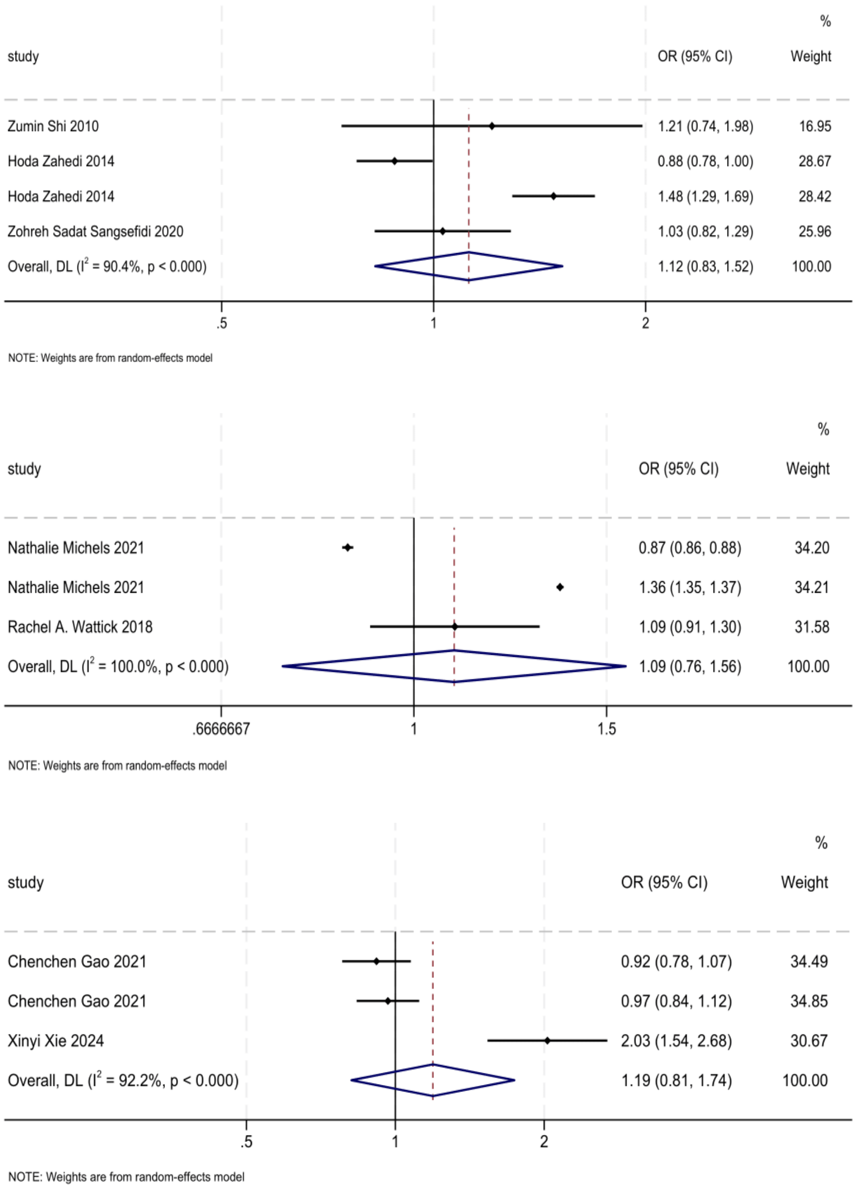

Supplementary Figure 2.1.6 Forest plot of (A) Quality Scores of Cross-Sectional Study (7); (B) Quality Scores of Cross-Sectional Study (8); (C) Quality Scores of Cross-Sectional Study (9).

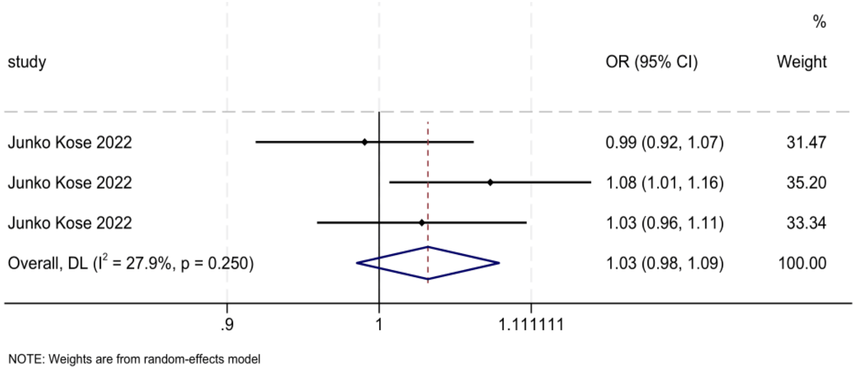

Supplementary Figure 2.1.7 Forest plot of (A) Quality Scores of Cohort Study (8).

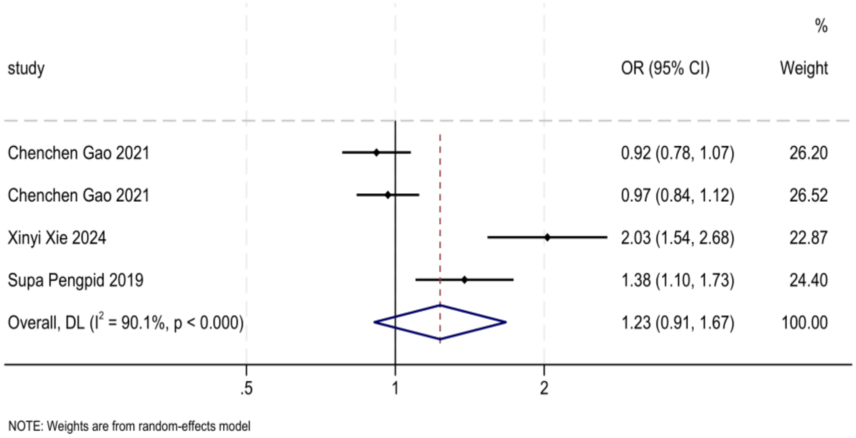

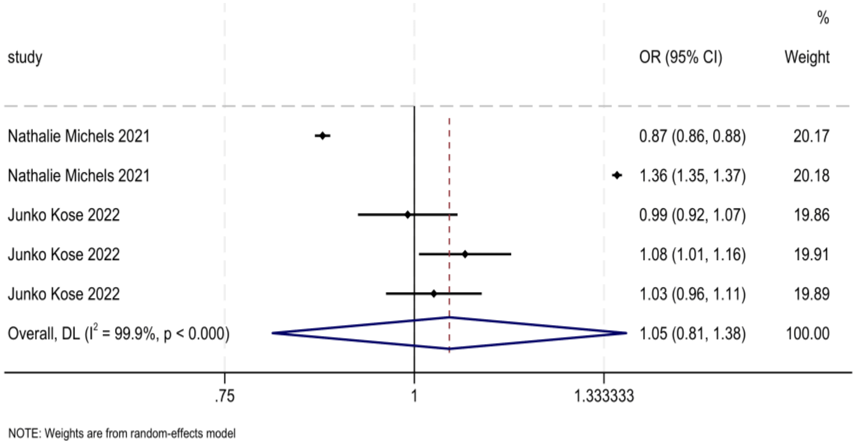

Supplementary Figure 2.1.8 Forest plot of (A) Region (Asia); (B) Region (European).
