# Supplementary material for: Association of sugar consumption with risk of depression and anxiety: a systematic review and meta-analysis
Source: Front Nutr. 2024 Oct 16;11:1472612. doi: 10.3389/fnut.2024.1472612 (PMC11522855; doi:10.3389/fnut.2024.1472612)
Supplement: Supplementary file 2 [file Image_2.pdf]

Supplementary Material

1 Funnel plot of the relationship between sugar intake and depression

1.1 overall analysis

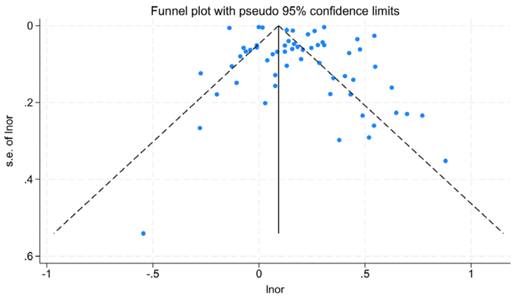

Supplementary Figure 1.1.1 Funnel plot of (A) overall analysis of the relationship between sugar intake and depression

1.2 subgroup analysis

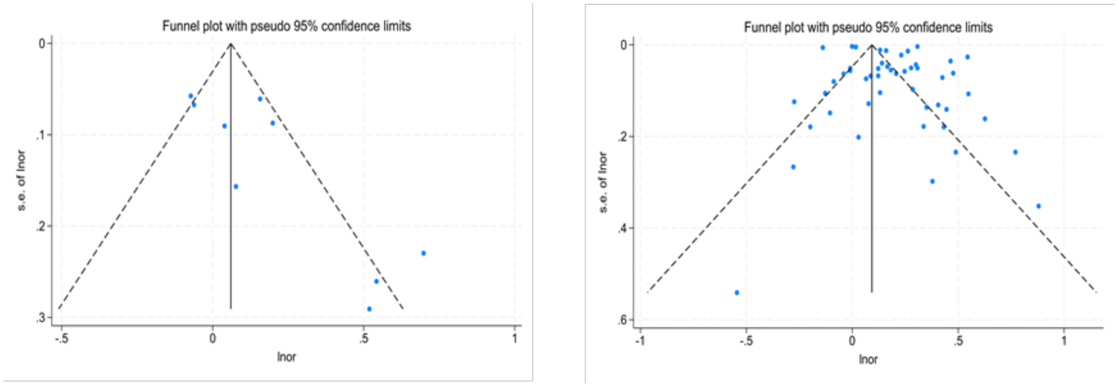

Supplementary Figure 2.2.1 Funnel plot of (A) Study Designing (cohort); (B) Study Designing (cross-sectional).

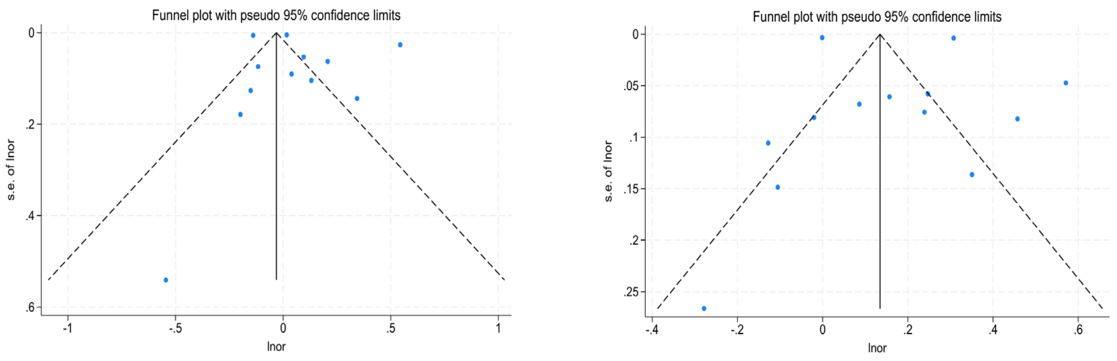

Supplementary Figure 3.2.2 Funnel plot of (A) Sex (male); (B) Sex (female).

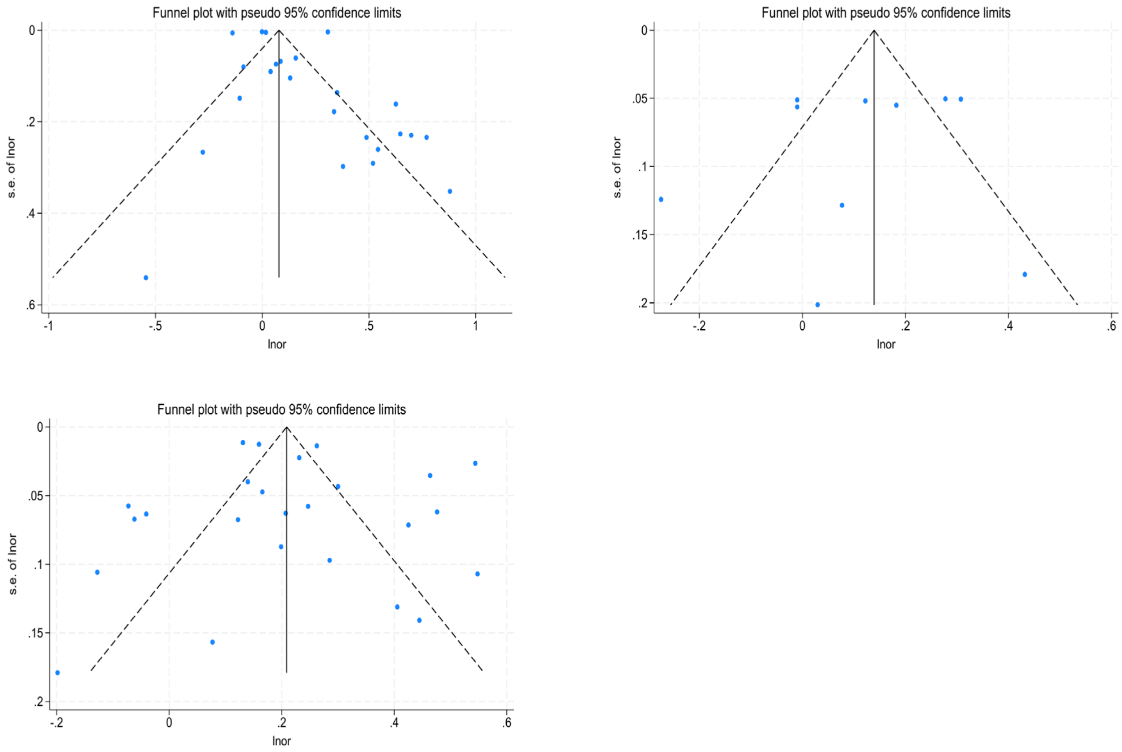

Supplementary Figure 4.2.3 Funnel plot of (A) Sample Size (＜5000); (B) Sample Size (5000-10000); (C) Sample Size (＞10000).

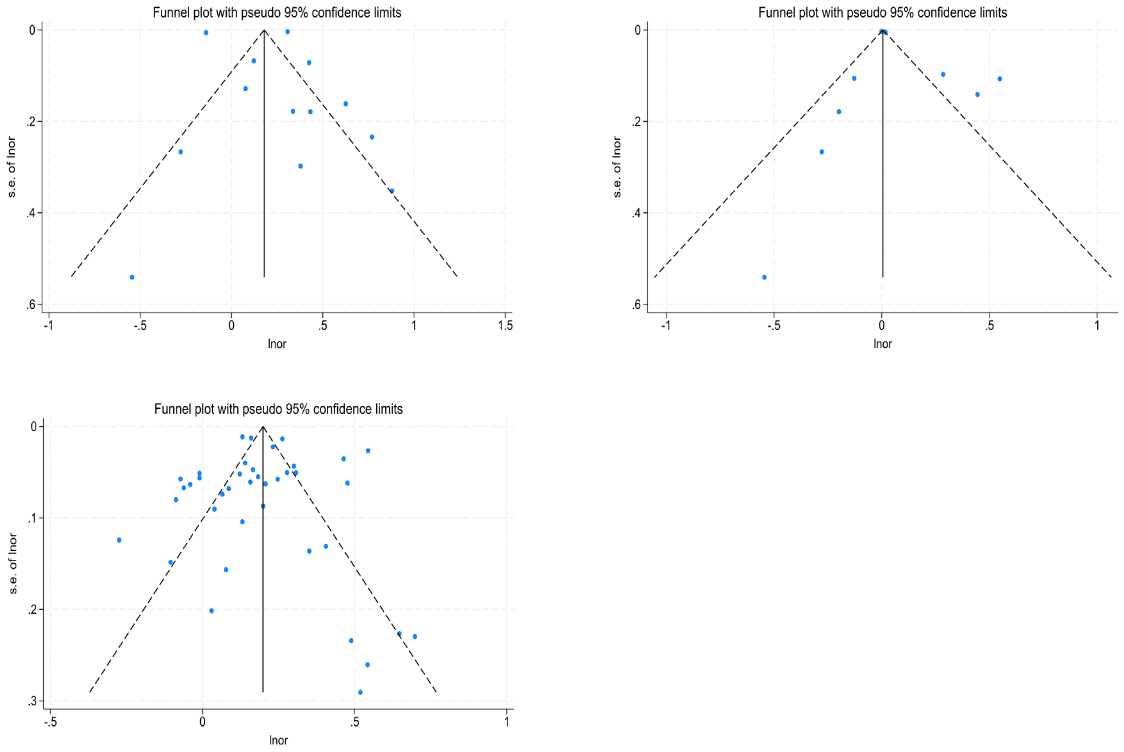

Supplementary Figure 5.2.4 Funnel plot of (A) Exposure Measures (FFQ); (B) Exposure Measures (24-h dietary recall); (B) Exposure Measures (self-made questionnaire).

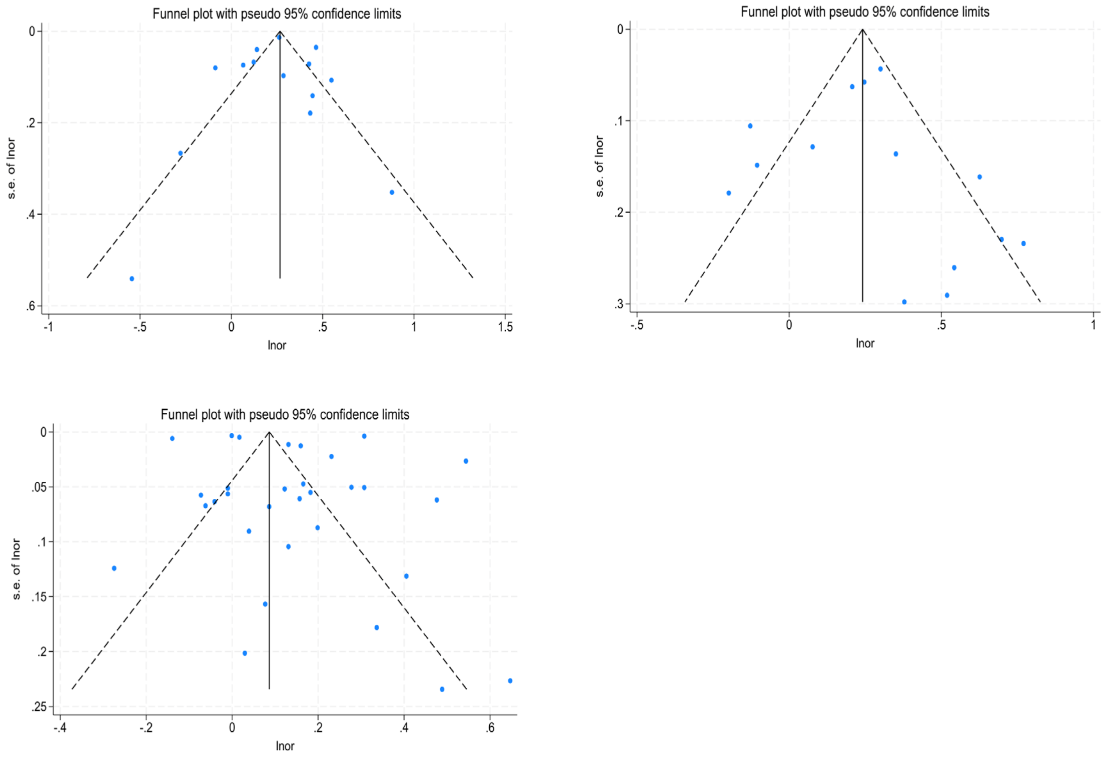

Supplementary Figure 6.2.5 Funnel plot of (A) Outcome Assessment (PHQ-9); (B) Outcome Assessment (CES); (C) Outcome Assessment (others).

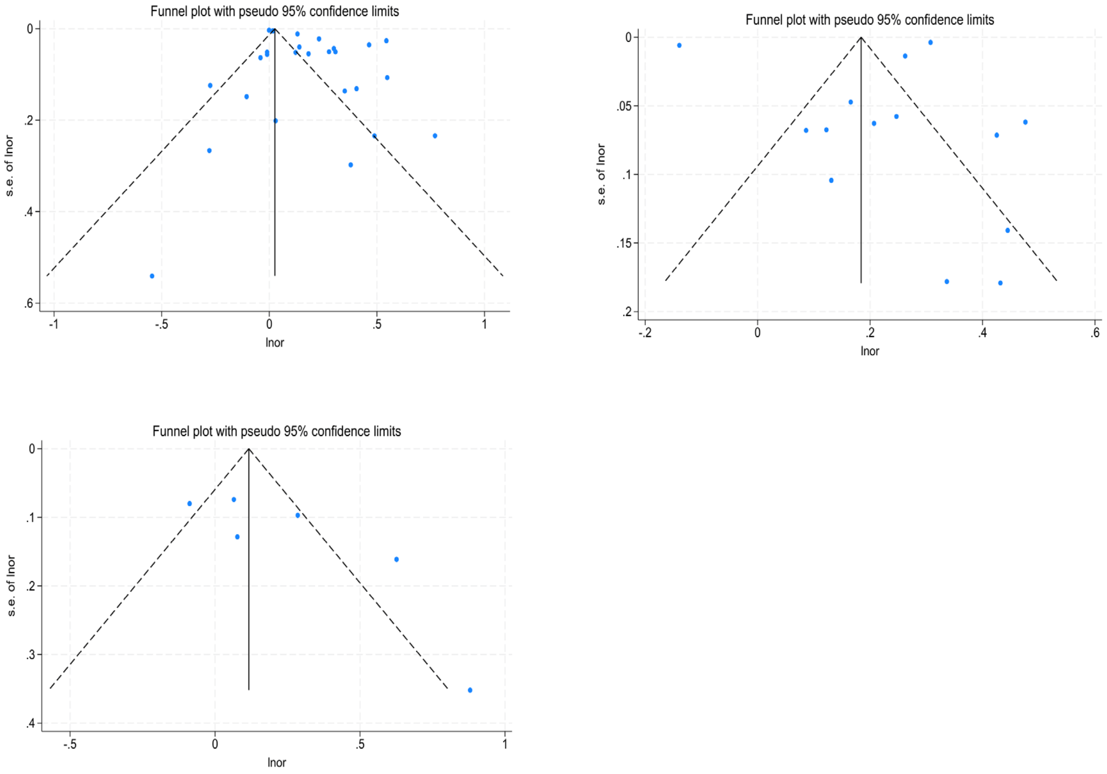

Supplementary Figure 7.2.6 Funnel plot of (A) Quality Scores of Cross-Sectional Study (7); (B) Quality Scores of Cross-Sectional Study (8); (C) Quality Scores of Cross-Sectional Study (9).

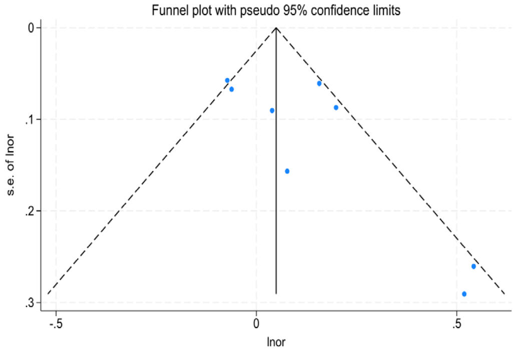

Supplementary Figure 8.2.7 Funnel plot of (A) Quality Scores of Cohort Study (7).

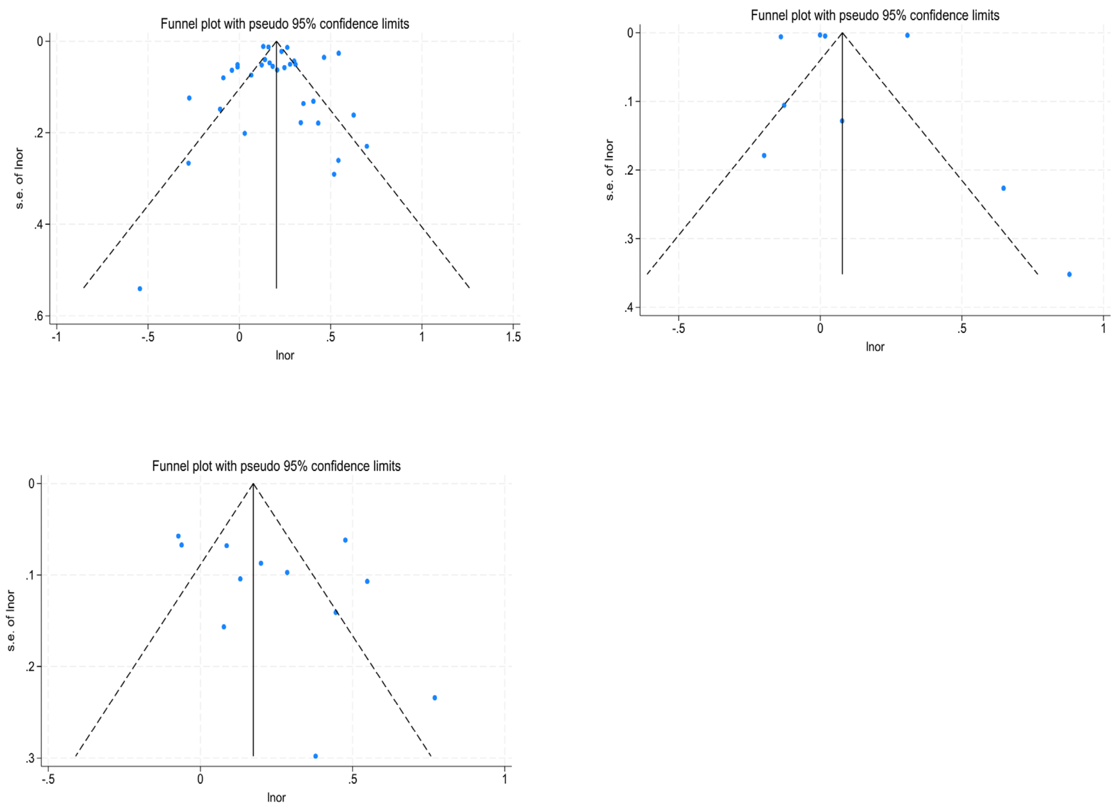

Supplementary Figure 9.2.8 Funnel plot of (A) Region (Asia); (B) Region (European); (C) Region (North America).

2 Funnel plot of the relationship between sugar intake and anxiety

2.1 overall analysis

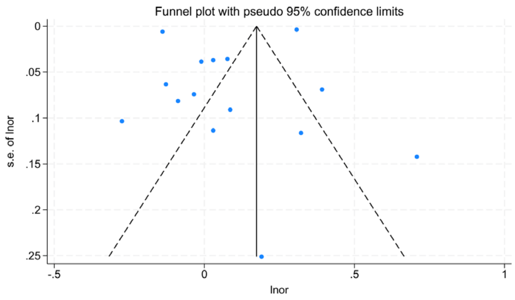

Supplementary Figure 2.1.1 Funnel plot of (A) overall analysis of the relationship between sugar intake and anxiety

2.2 subgroup analysis

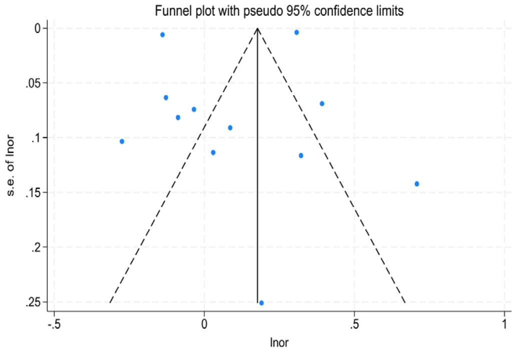

Supplementary Figure 2.2.1 Funnel plot of (A) Study Designing (cross-sectional).

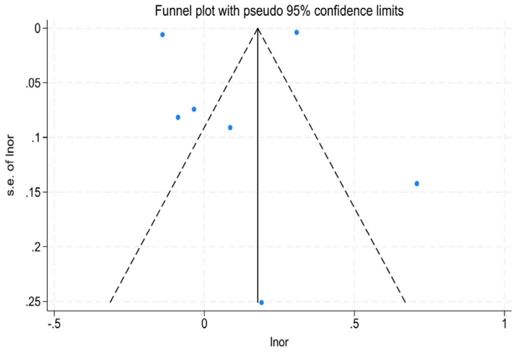

Supplementary Figure 2.2.2 Funnel plot of (A) Sample Size (＜5000).

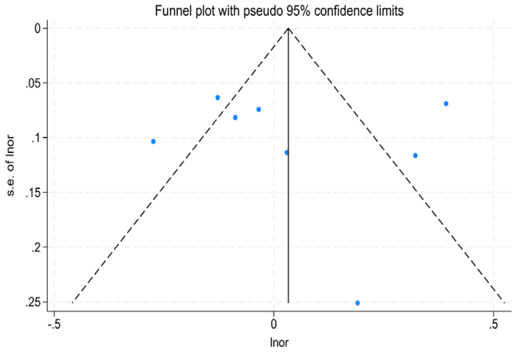

Supplementary Figure 2.2.3 Funnel plot of (A) Exposure Measures (others).

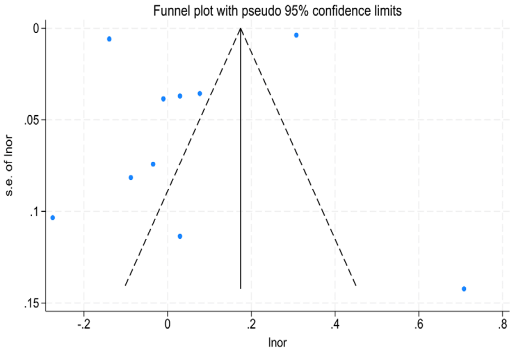

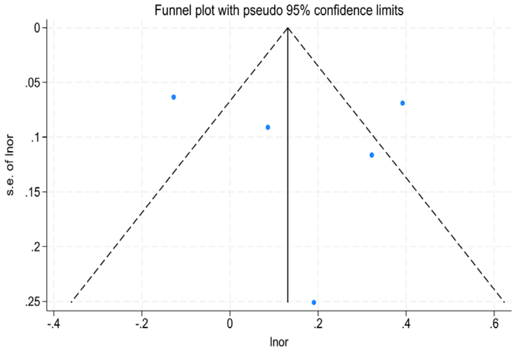

Supplementary Figure 2.2.4 Funnel plot of (A) Outcome Assessment (GAD-7, SCL-90, STAI, DASS21); (B) Outcome Assessment (others).
