# Supplementary material for: Association of sugar consumption with risk of depression and anxiety: a systematic review and meta-analysis
Source: Front Nutr. 2024 Oct 16;11:1472612. doi: 10.3389/fnut.2024.1472612 (PMC11522855; doi:10.3389/fnut.2024.1472612)
Supplement: Supplementary file 3 [file Image_3.pdf]

Supplementary Material

1 Sensitivity analysis of the relationship between sugar intake and depression

1.1 overall analysis

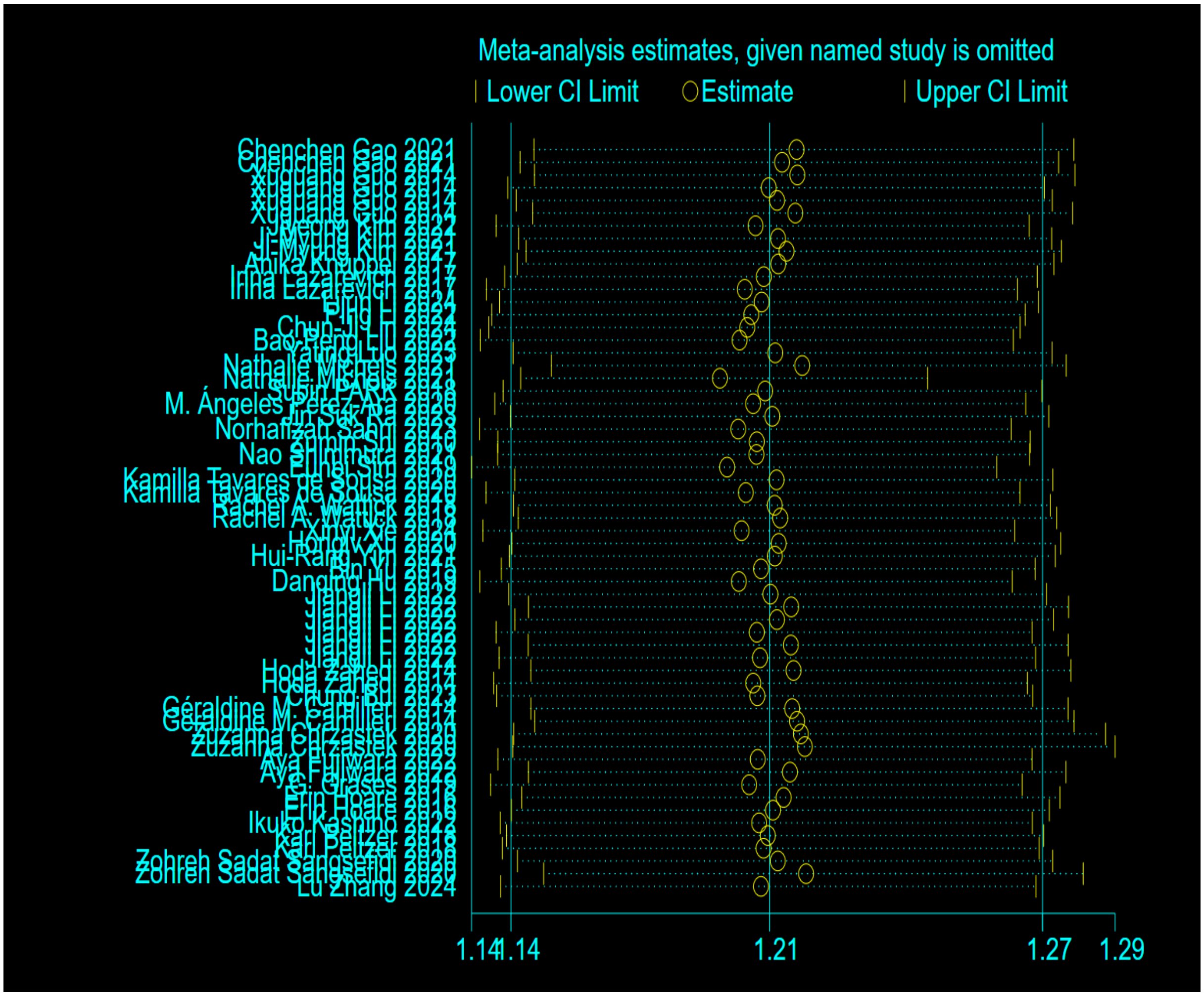

Supplementary Figure 1.1.1 Sensitivity analysis of (A) overall analysis of the relationship between sugar intake and depression

1.2 subgroup analysis

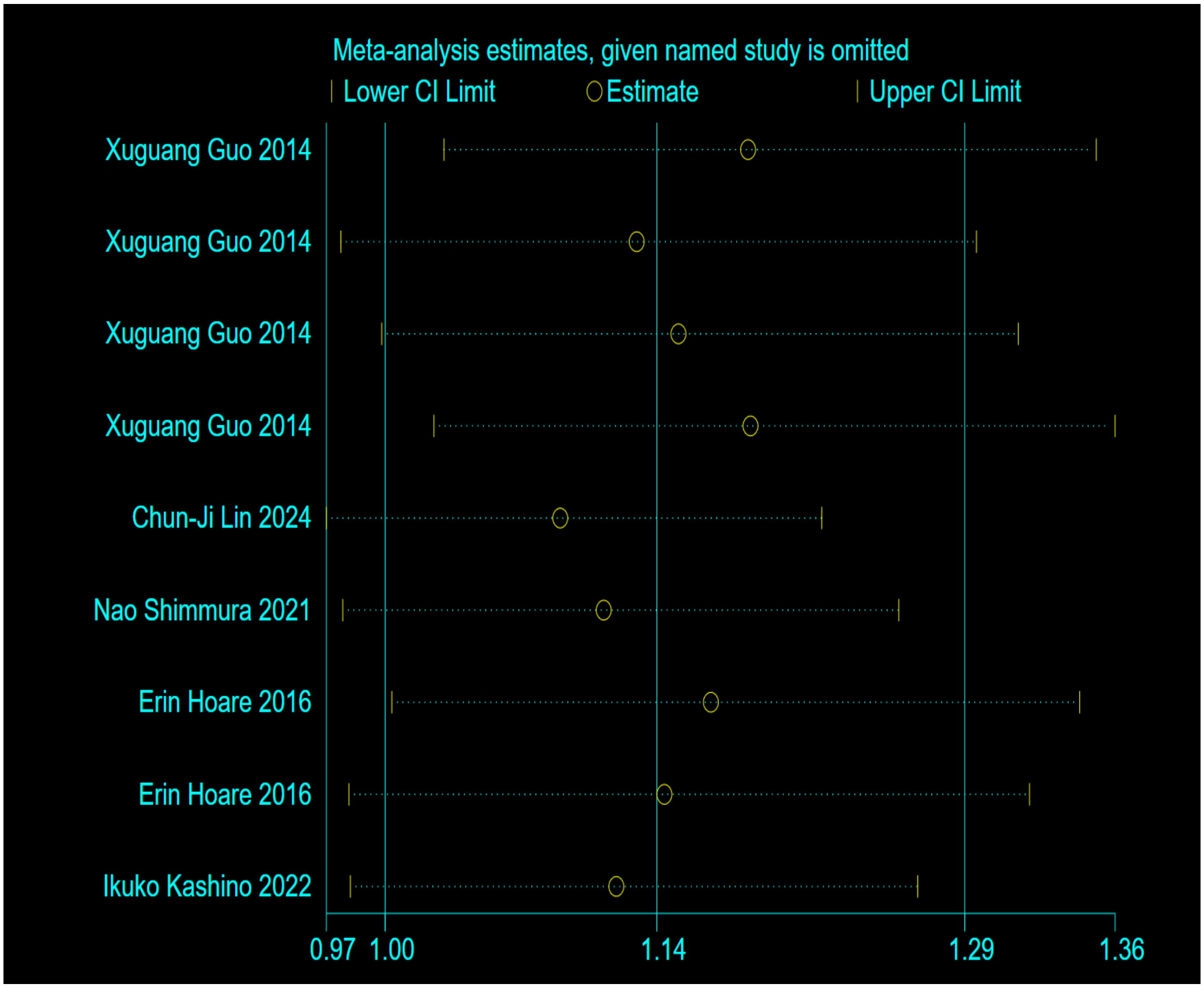
Meta-analysis estimates, given named study is omitted
Lower CI Limit
Estimate
Upper CI Limit
Xuguang Guo 2014
Xuguang Guo 2014
Xuguang Guo 2014
Xuguang Guo 2014
Chun-Ji Lin 2024
Nao Shimmura 2021
Erin Hoare 2016
Erin Hoare 2016
Ikuko Kashino 2022
0.97
1.00
1.14
1.29
1.36

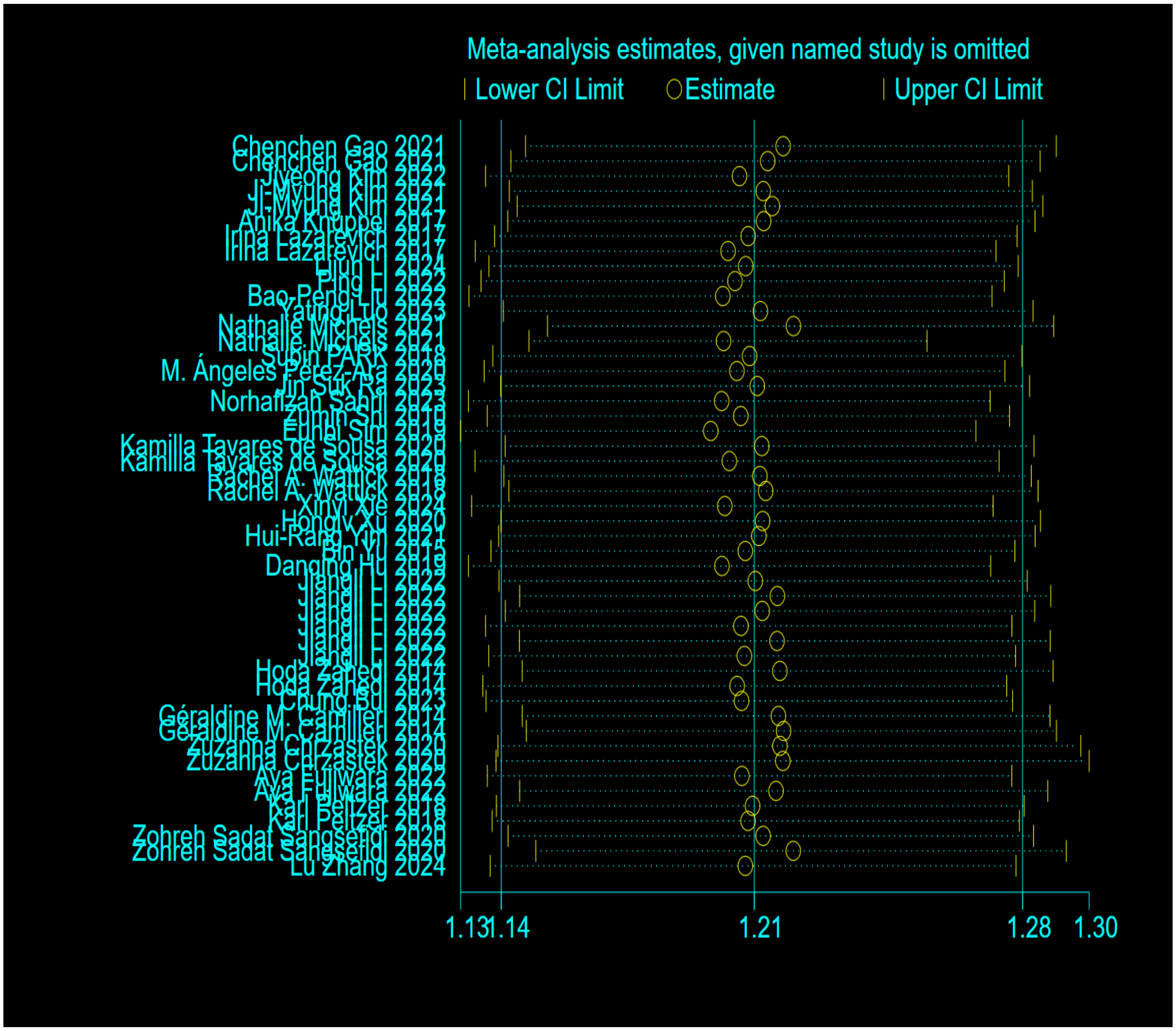

Supplementary Figure 2.2.1 Sensitivity analysis of (A) Study Designing (cohort); (B) Study Designing (cross-sectional).

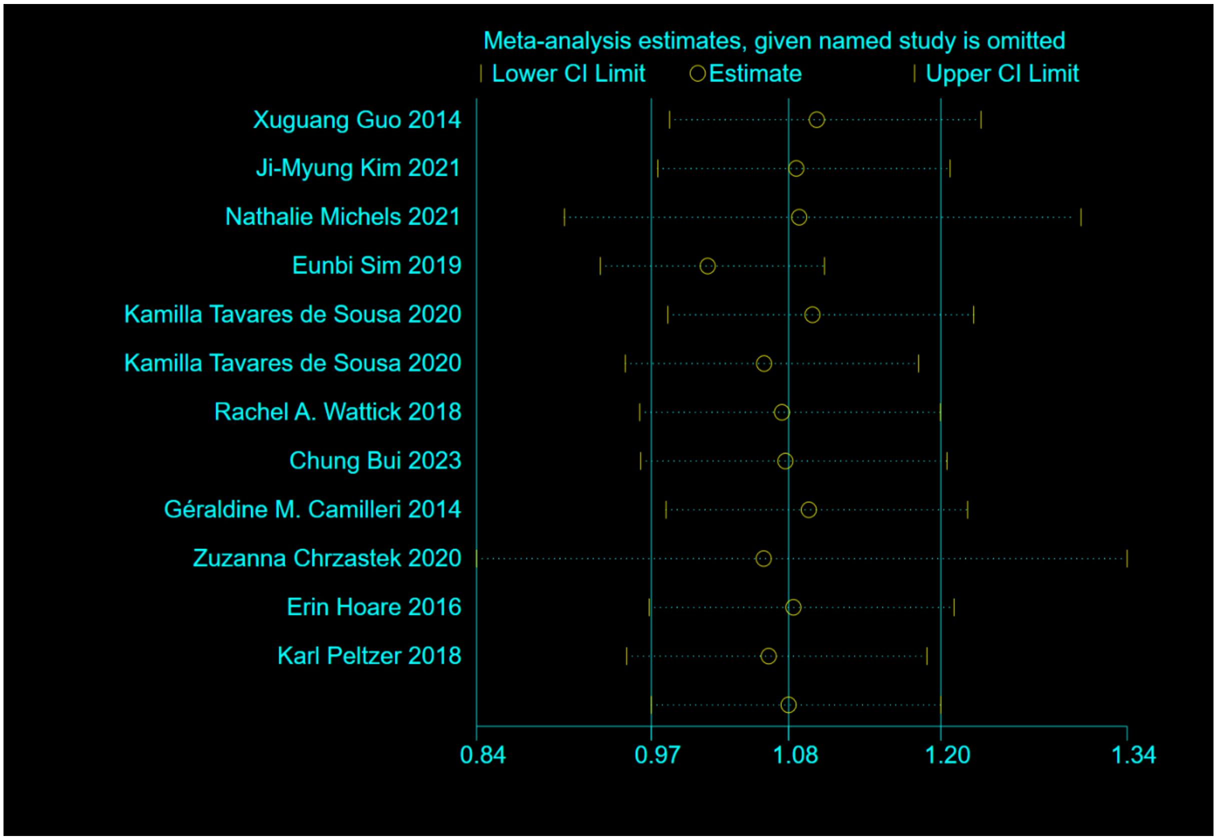
Meta-analysis estimates, given named study is omitted
Lower CI Limit
Estimate
Upper CI Limit
Xuguang Guo 2014
Ji-Myung Kim 2021
Nathalie Michels 2021
Eunbi Sim 2019
Kamilla Tavares de Sousa 2020
Kamilla Tavares de Sousa 2020
Rachel A. Wattick 2018
Chung Bui 2023
Géraldine M. Camilleri 2014
Zuzanna Chrzastek 2020
Erin Hoare 2016
Karl Peltzer 2018
0.84
0.97
1.08
1.20
1.34

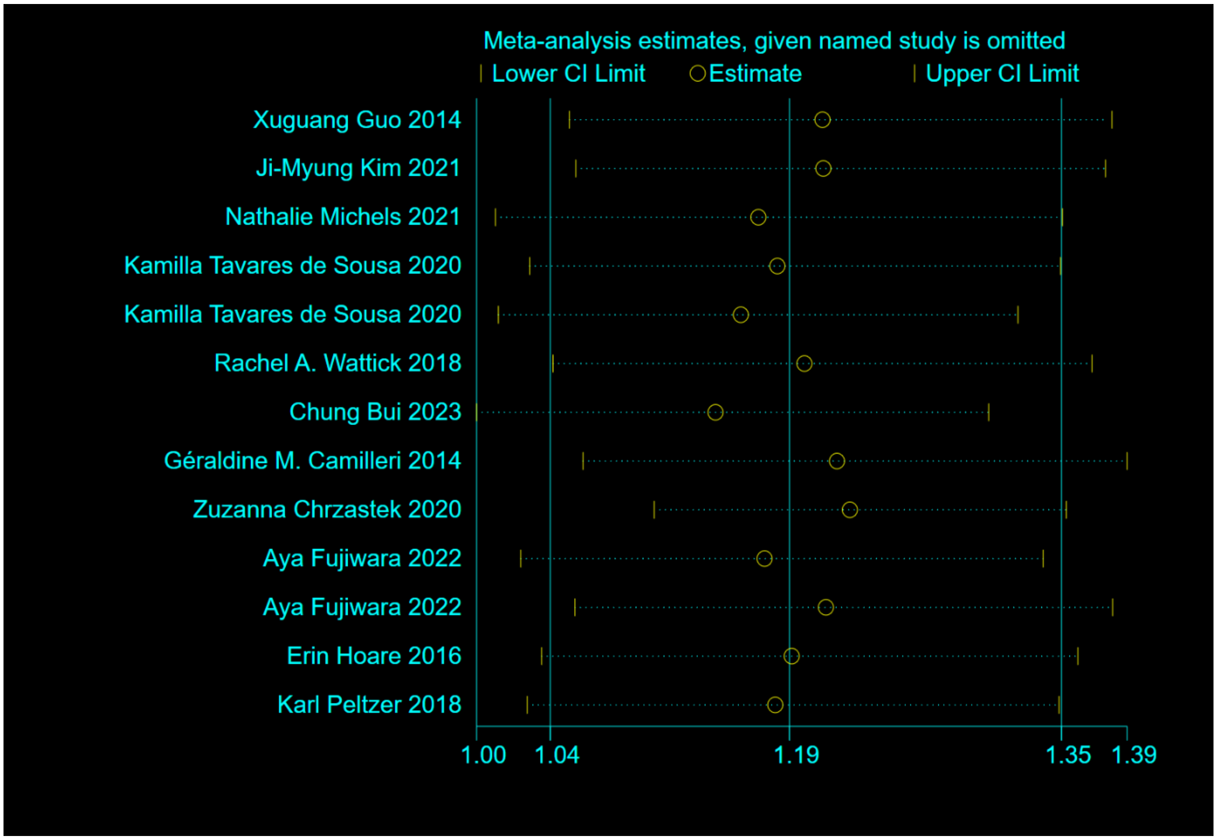
Meta-analysis estimates, given named study is omitted
Lower CI Limit
Estimate
Upper CI Limit
Xuguang Guo 2014
Ji-Myung Kim 2021
Nathalie Michels 2021
Kamilla Tavares de Sousa 2020
Kamilla Tavares de Sousa 2020
Rachel A. Wattick 2018
Chung Bui 2023
Géraldine M. Camilleri 2014
Zuzanna Chrzastek 2020
Aya Fujiwara 2022
Aya Fujiwara 2022
Erin Hoare 2016
Karl Peltzer 2018
1.00
1.04
1.19
1.35
1.39

Supplementary Figure 3.2.2 Sensitivity analysis of (A) Sex (male); (B) Sex (female).

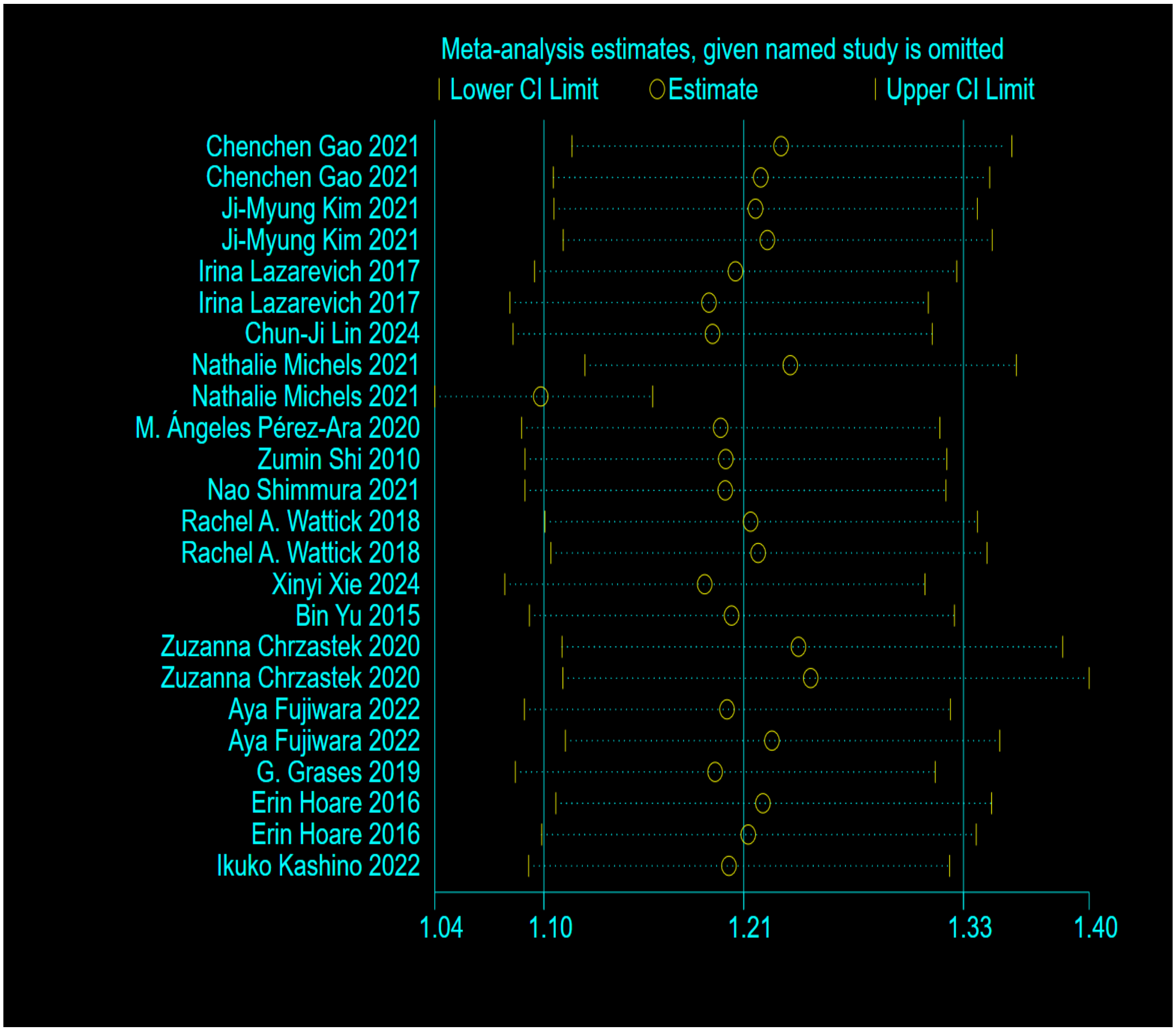

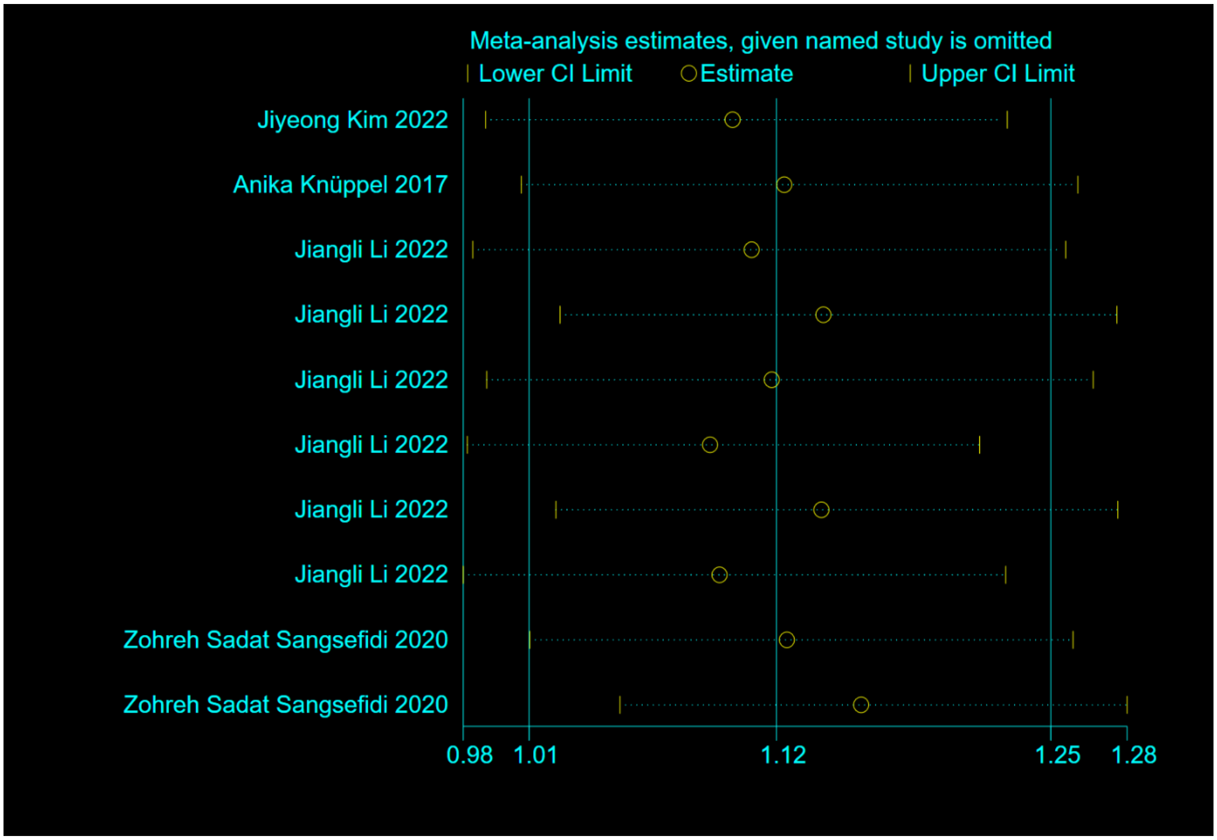
Meta-analysis estimates, given named study is omitted
Lower CI Limit
Estimate
Upper CI Limit
Jiyeong Kim 2022
Anika Knüppel 2017
Jiangli Li 2022
Jiangli Li 2022
Jiangli Li 2022
Jiangli Li 2022
Jiangli Li 2022
Jiangli Li 2022
Zohreh Sadat Sangsefidi 2020
Zohreh Sadat Sangsefidi 2020
0.98
1.01
1.12
1.25
1.28

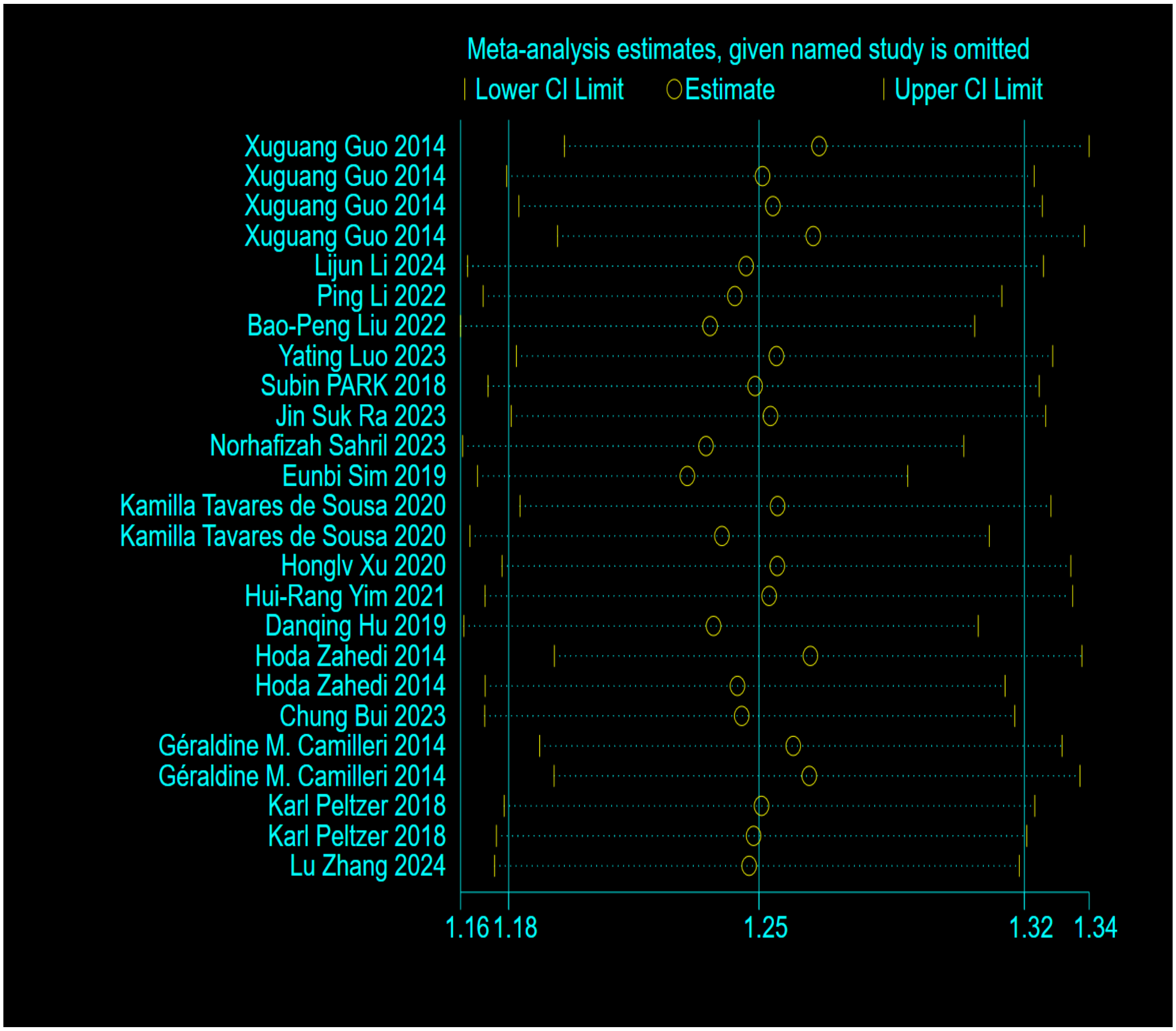

Supplementary Figure 4.2.3 Sensitivity analysis of (A) Sample Size (＜5000); (B) Sample Size (5000-10000); (C) Sample Size (＞10000).

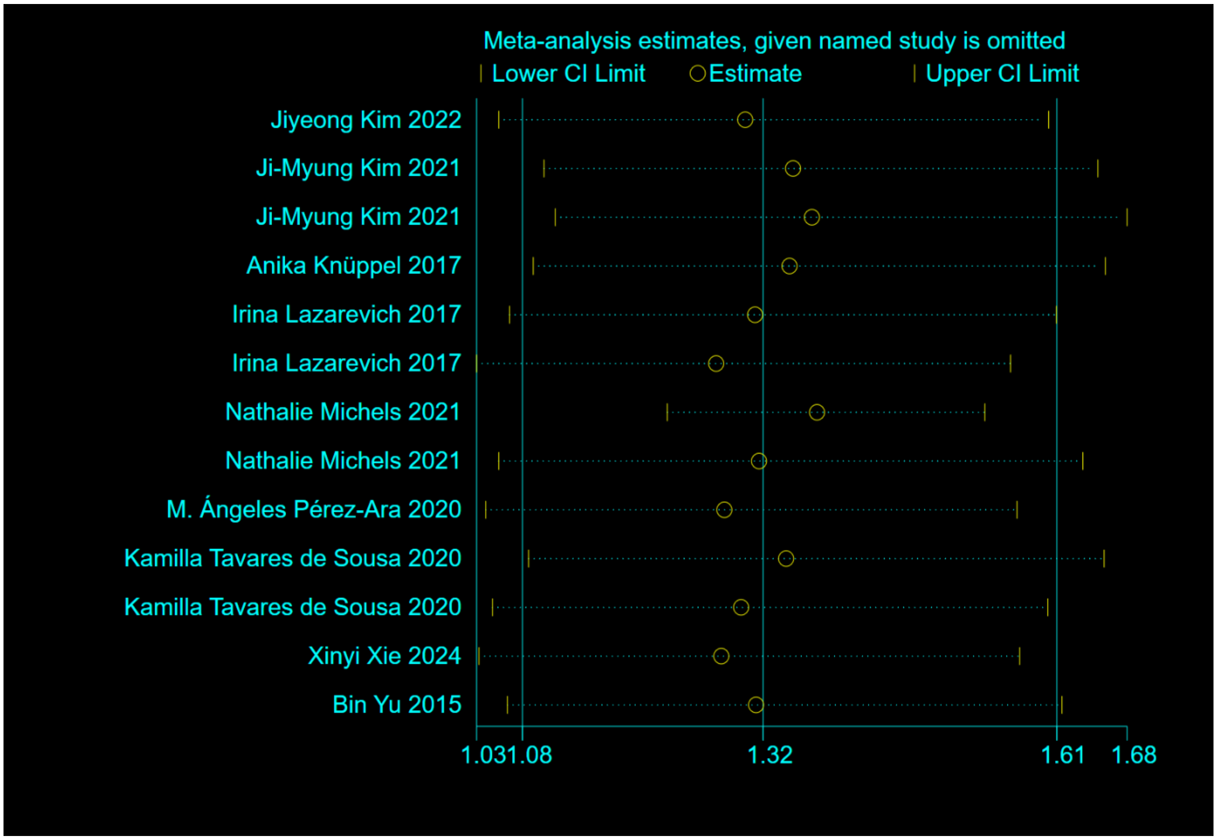
Meta-analysis estimates, given named study is omitted
Lower CI Limit
Estimate
Upper CI Limit
Jiyeong Kim 2022
Ji-Myung Kim 2021
Ji-Myung Kim 2021
Anika Knüppel 2017
Irina Lazarevich 2017
Irina Lazarevich 2017
Nathalie Michels 2021
Nathalie Michels 2021
M. Ángeles Pérez-Ara 2020
Kamilla Tavares de Sousa 2020
Kamilla Tavares de Sousa 2020
Xinyi Xie 2024
Bin Yu 2015
1.03
1.08
1.32
1.61
1.68

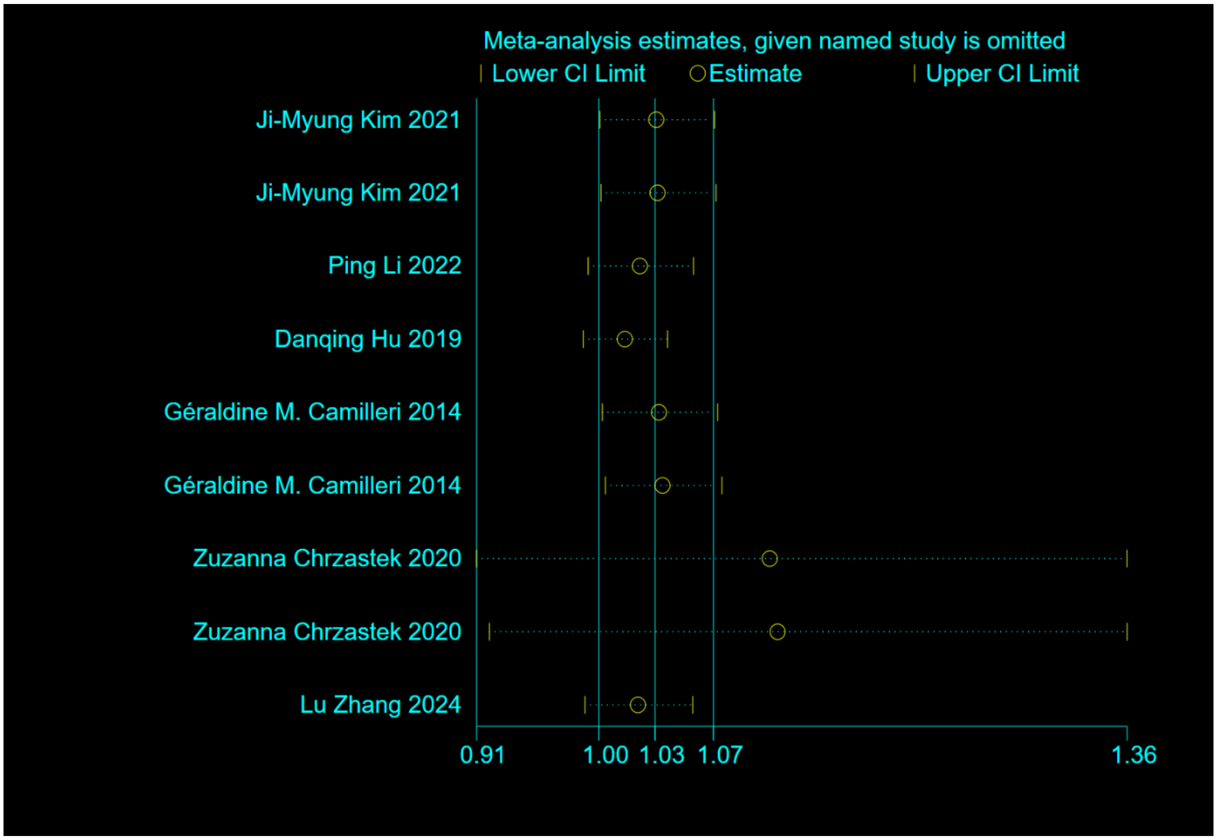
Meta-analysis estimates, given named study is omitted
Lower CI Limit
Estimate
Upper CI Limit
Ji-Myung Kim 2021
Ji-Myung Kim 2021
Ping Li 2022
Danqing Hu 2019
Géraldine M. Camilleri 2014
Géraldine M. Camilleri 2014
Zuzanna Chrzastek 2020
Zuzanna Chrzastek 2020
Lu Zhang 2024
0.91
1.00
1.03
1.07
1.36

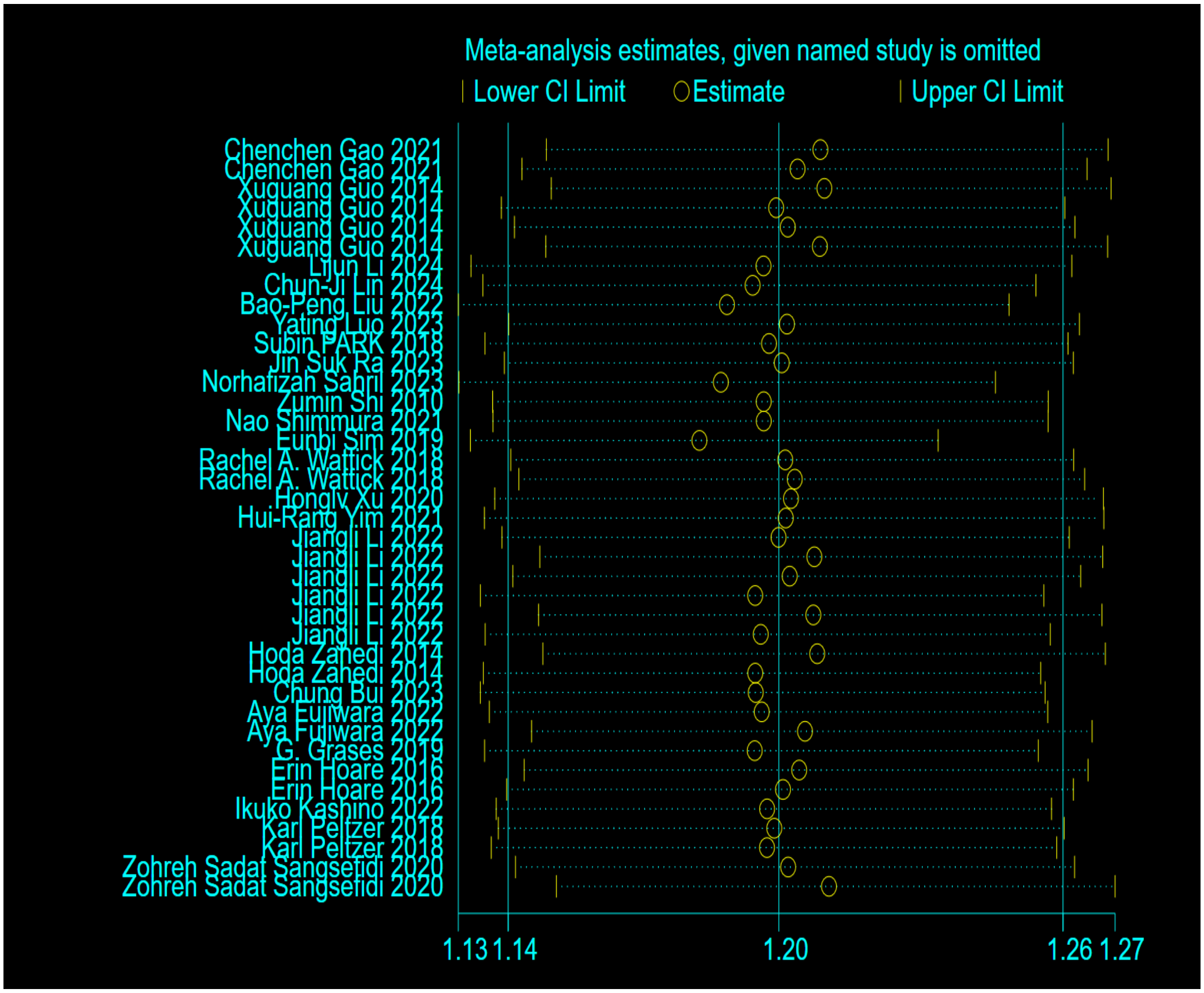

Supplementary Figure 5.2.4 Sensitivity analysis of (A) Exposure Measures (FFQ); (B) Exposure Measures (24-h dietary recall); (B) Exposure Measures (self-made questionnaire).

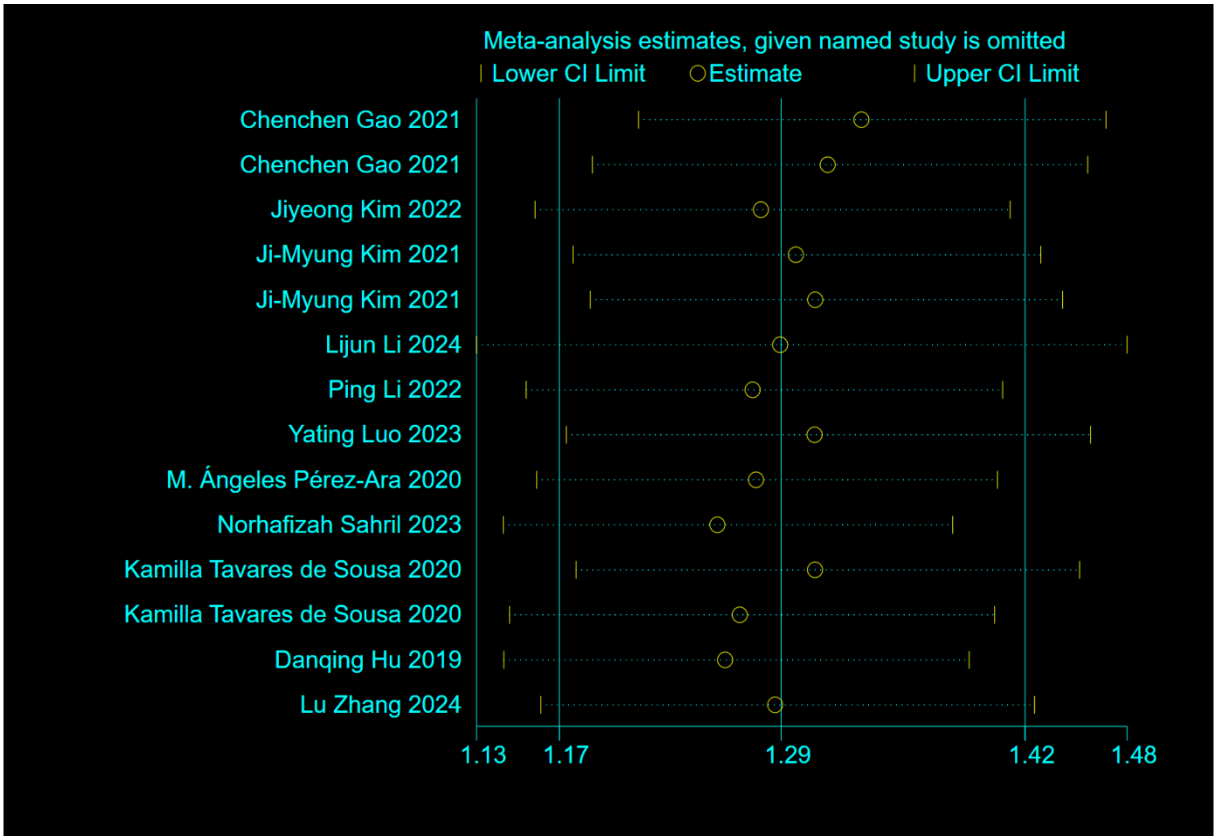
Meta-analysis estimates, given named study is omitted
Lower CI Limit
Estimate
Upper CI Limit
Chenchen Gao 2021
Chenchen Gao 2021
Jiyeong Kim 2022
Ji-Myung Kim 2021
Ji-Myung Kim 2021
Lijun Li 2024
Ping Li 2022
Yating Luo 2023
M. Ángeles Pérez-Ara 2020
Norhafizah Sahril 2023
Kamilla Tavares de Sousa 2020
Kamilla Tavares de Sousa 2020
Danqing Hu 2019
Lu Zhang 2024
1.13
1.17
1.29
1.42
1.48

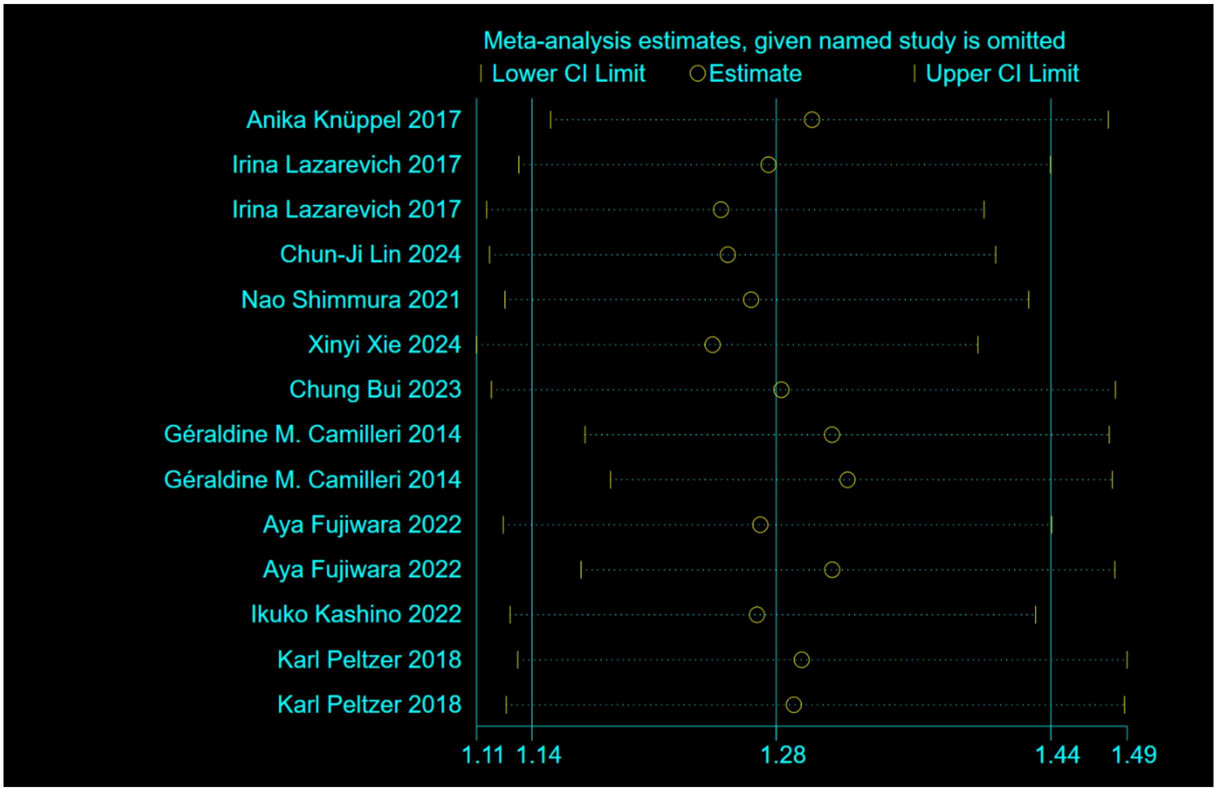
Meta-analysis estimates, given named study is omitted
Lower CI Limit
Estimate
Upper CI Limit
Anika Knüppel 2017
Irina Lazarevich 2017
Irina Lazarevich 2017
Chun-Ji Lin 2024
Nao Shimmura 2021
Xinyi Xie 2024
Chung Bui 2023
Géraldine M. Camilleri 2014
Géraldine M. Camilleri 2014
Aya Fujiwara 2022
Aya Fujiwara 2022
Ikuko Kashino 2022
Karl Peltzer 2018
Karl Peltzer 2018
1.11
1.14
1.28
1.44
1.49

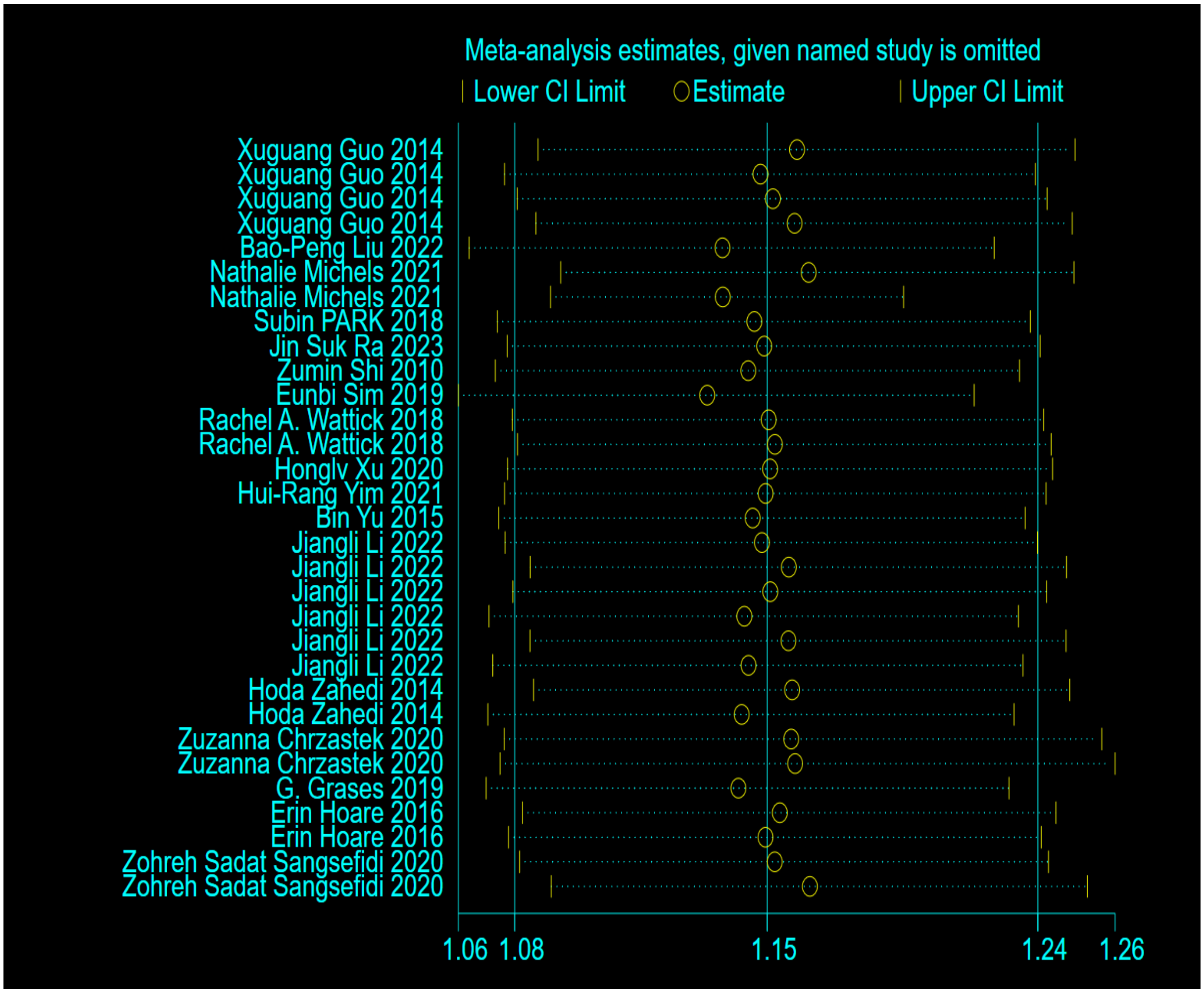

Supplementary Figure 6.2.5 Sensitivity analysis of (A) Outcome Assessment (PHQ-9); (B) Outcome Assessment (CES); (C) Outcome Assessment (others).

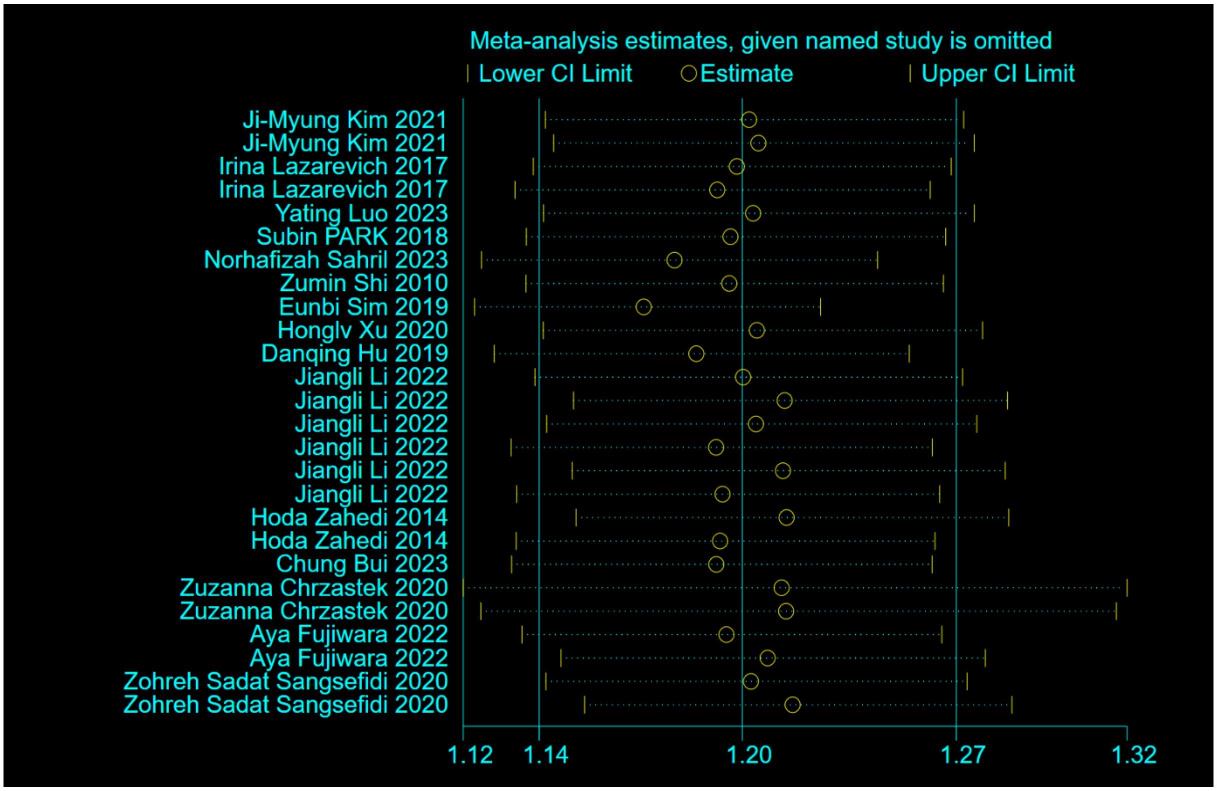
Meta-analysis estimates, given named study is omitted
Lower CI Limit
Estimate
Upper CI Limit
Ji-Myung Kim 2021
Ji-Myung Kim 2021
Irina Lazarevich 2017
Irina Lazarevich 2017
Yating Luo 2023
Subin PARK 2018
Norhafizah Sahril 2023
Zumin Shi 2010
Eunbi Sim 2019
Honglv Xu 2020
Danqing Hu 2019
Jiangli Li 2022
Jiangli Li 2022
Jiangli Li 2022
Jiangli Li 2022
Jiangli Li 2022
Jiangli Li 2022
Hoda Zahedi 2014
Hoda Zahedi 2014
Chung Bui 2023
Zuzanna Chrzastek 2020
Zuzanna Chrzastek 2020
Aya Fujiwara 2022
Aya Fujiwara 2022
Zohreh Sadat Sangsefidi 2020
Zohreh Sadat Sangsefidi 2020
1.12
1.14
1.20
1.27
1.32

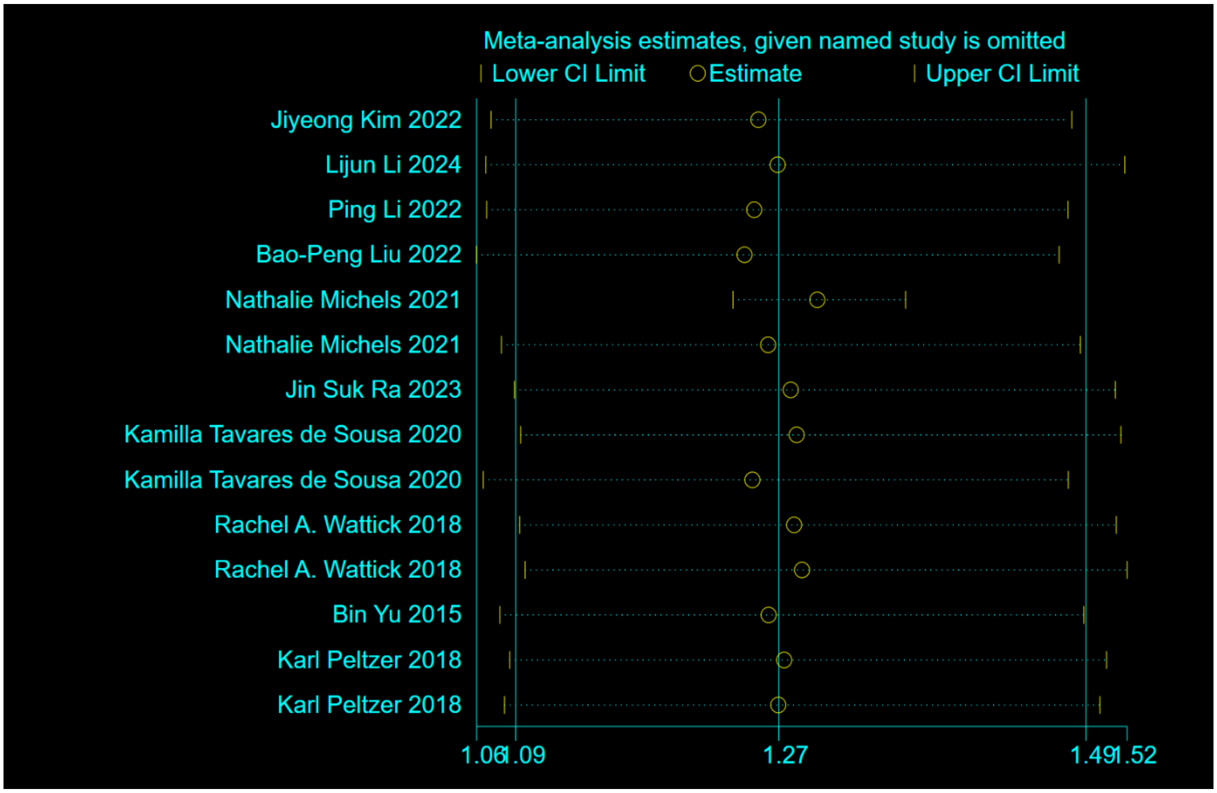
Meta-analysis estimates, given named study is omitted
Lower CI Limit
Estimate
Upper CI Limit
Jiyeong Kim 2022
Lijun Li 2024
Ping Li 2022
Bao-Peng Liu 2022
Nathalie Michels 2021
Nathalie Michels 2021
Jin Suk Ra 2023
Kamilla Tavares de Sousa 2020
Kamilla Tavares de Sousa 2020
Rachel A. Wattick 2018
Rachel A. Wattick 2018
Bin Yu 2015
Karl Peltzer 2018
Karl Peltzer 2018
1.06
1.09
1.27
1.49
1.52

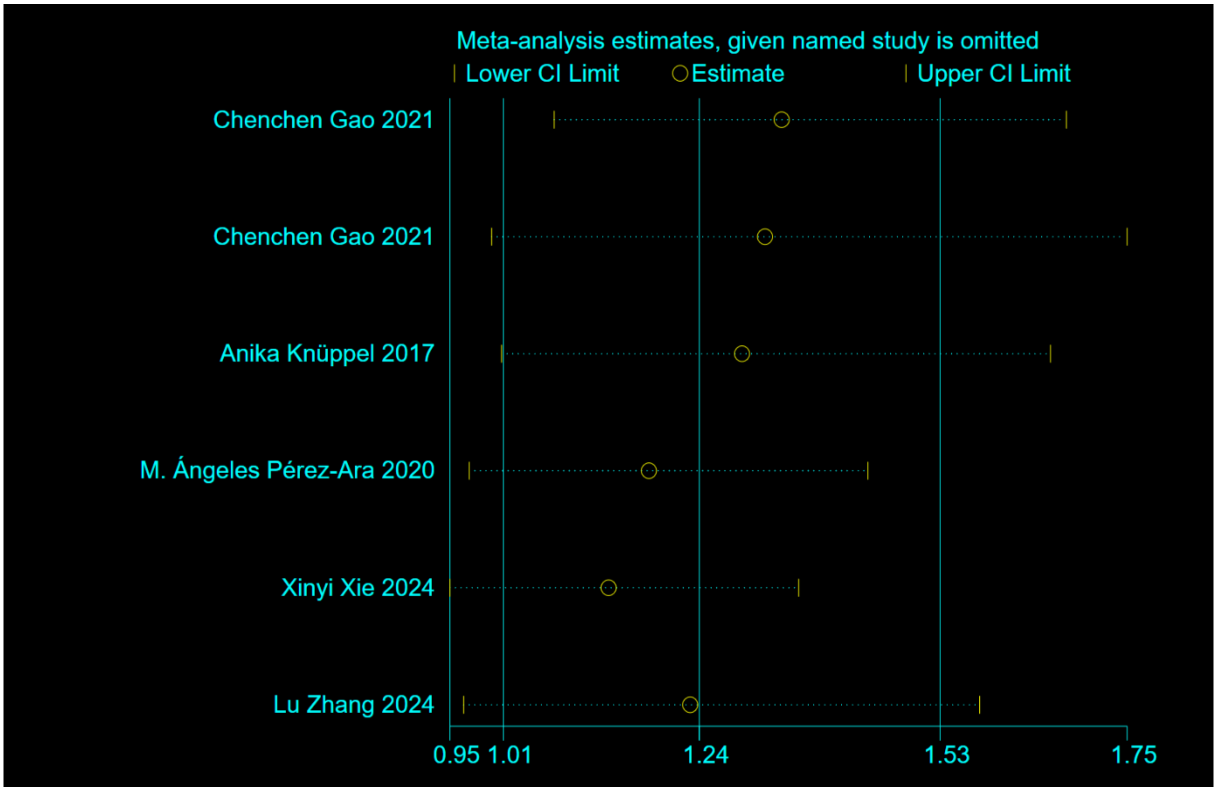

Supplementary Figure 7.2.6 Sensitivity analysis of (A) Quality Scores of Cross-Sectional Study (7); (B) Quality Scores of Cross-Sectional Study (8); (C) Quality Scores of Cross-Sectional Study (9).

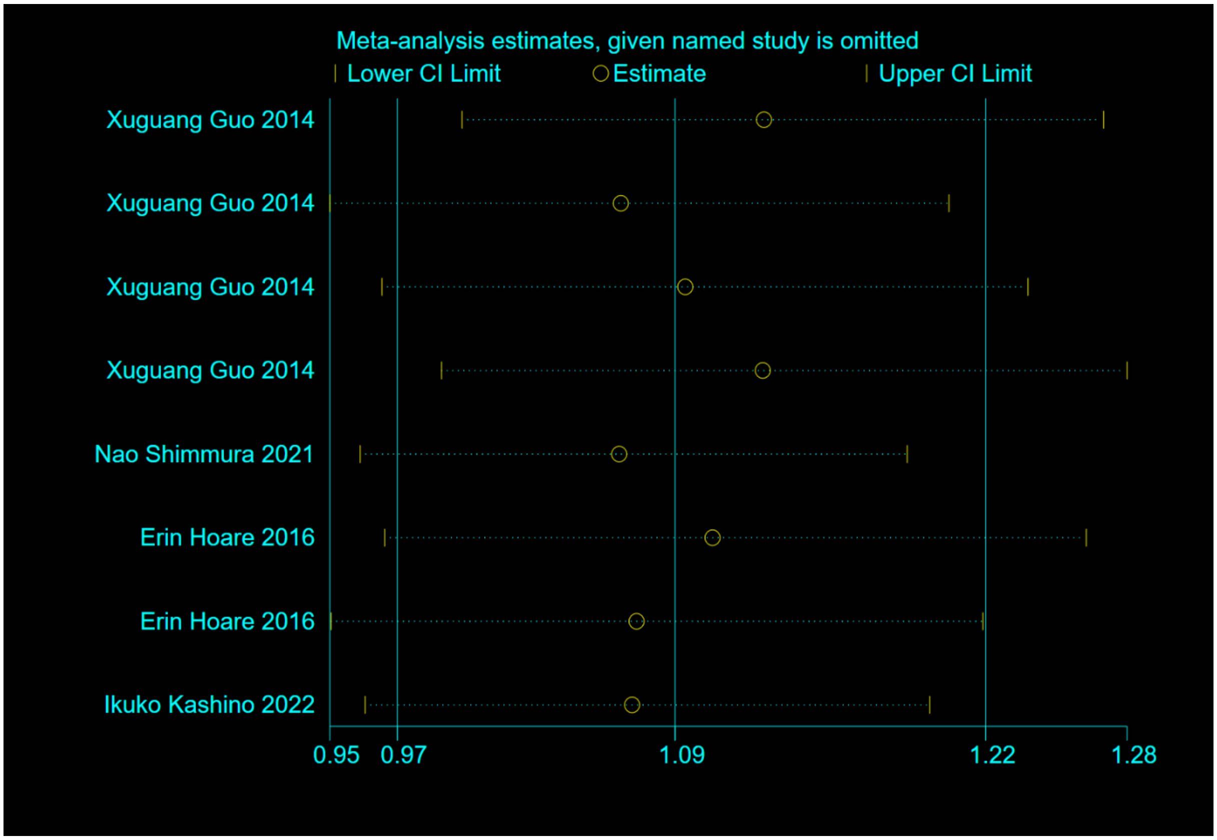

Supplementary Figure 8.2.7 Sensitivity analysis of (A) Quality Scores of Cohort Study (7).

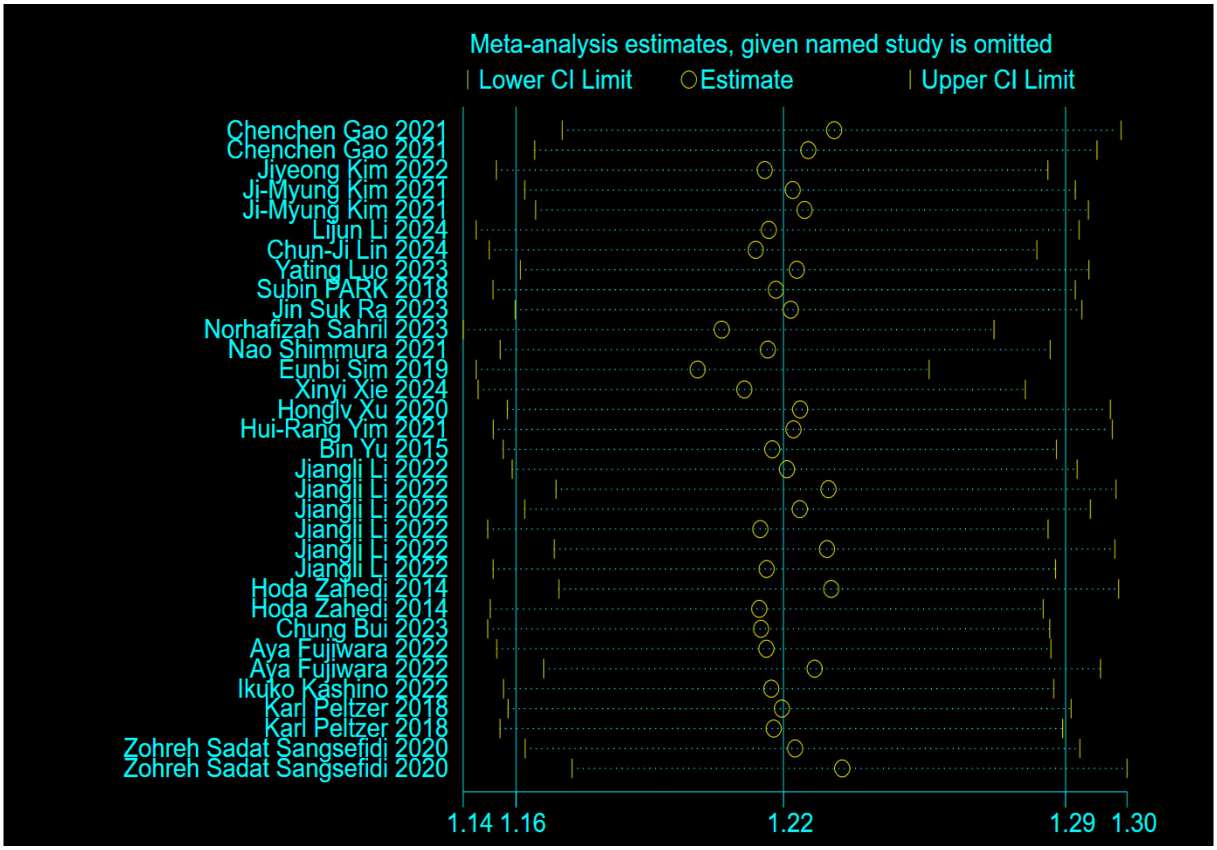
Meta-analysis estimates, given named study is omitted
Lower CI Limit
Estimate
Upper CI Limit
Chenchen Gao 2021
Chenchen Gao 2021
Jiyeong Kim 2022
Ji-Myung Kim 2021
Ji-Myung Kim 2021
Lijun Li 2024
Chun-Ji Lin 2024
Yating Luo 2023
Subin PARK 2018
Jin Suk Ra 2023
Norhafizah Sahril 2023
Nao Shimmura 2021
Eunbi Sim 2019
Xinyi Xie 2024
Honglv Xu 2020
Hui-Rang Yim 2021
Bin Yu 2015
Jiangli Li 2022
Jiangli Li 2022
Jiangli Li 2022
Jiangli Li 2022
Jiangli Li 2022
Jiangli Li 2022
Hoda Zahedi 2014
Hoda Zahedi 2014
Chung Bui 2023
Aya Fujiwara 2022
Aya Fujiwara 2022
Ikuko Kashino 2022
Karl Peltzer 2018
Karl Peltzer 2018
Zohreh Sadat Sangsefidi 2020
Zohreh Sadat Sangsefidi 2020
1.14
1.16
1.22
1.29
1.30

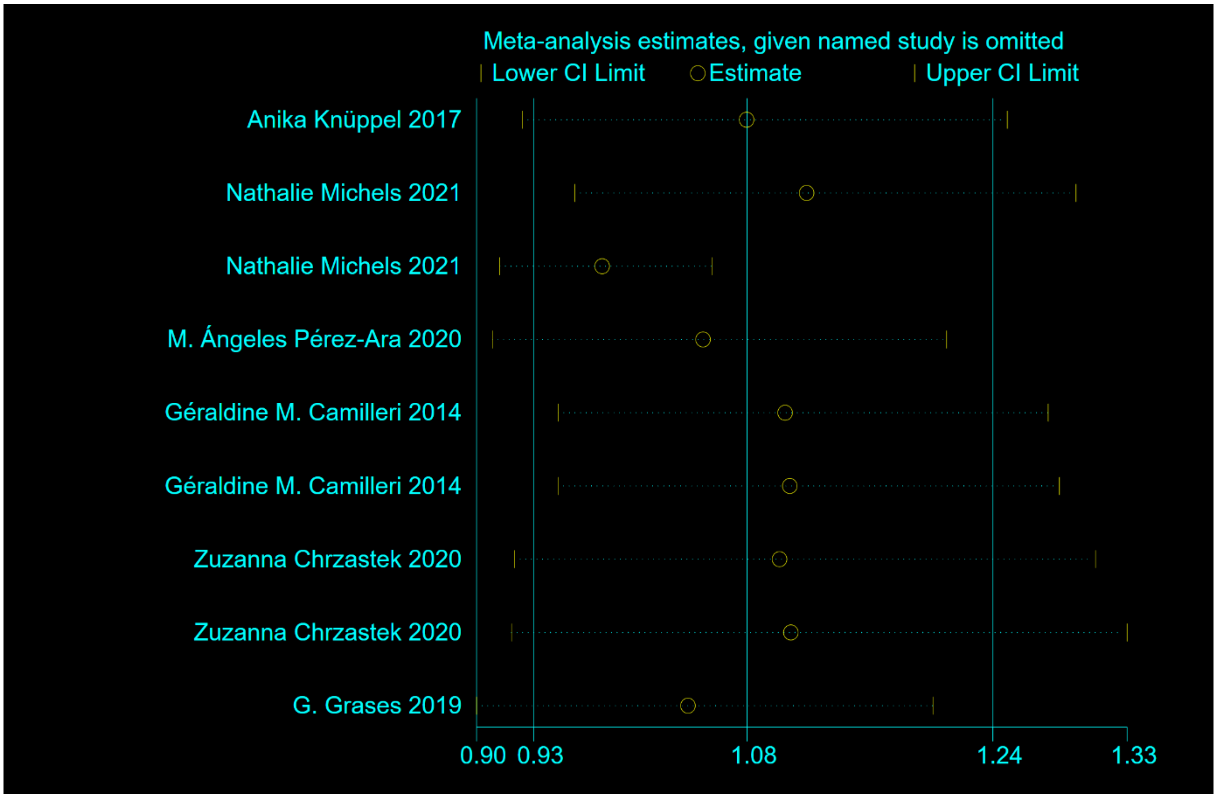
Meta-analysis estimates, given named study is omitted
Lower CI Limit
Estimate
Upper CI Limit
Anika Knüppel 2017
Nathalie Michels 2021
Nathalie Michels 2021
M. Ángeles Pérez-Ara 2020
Géraldine M. Camilleri 2014
Géraldine M. Camilleri 2014
Zuzanna Chrzastek 2020
Zuzanna Chrzastek 2020
G. Grases 2019
0.90
0.93
1.08
1.24
1.33

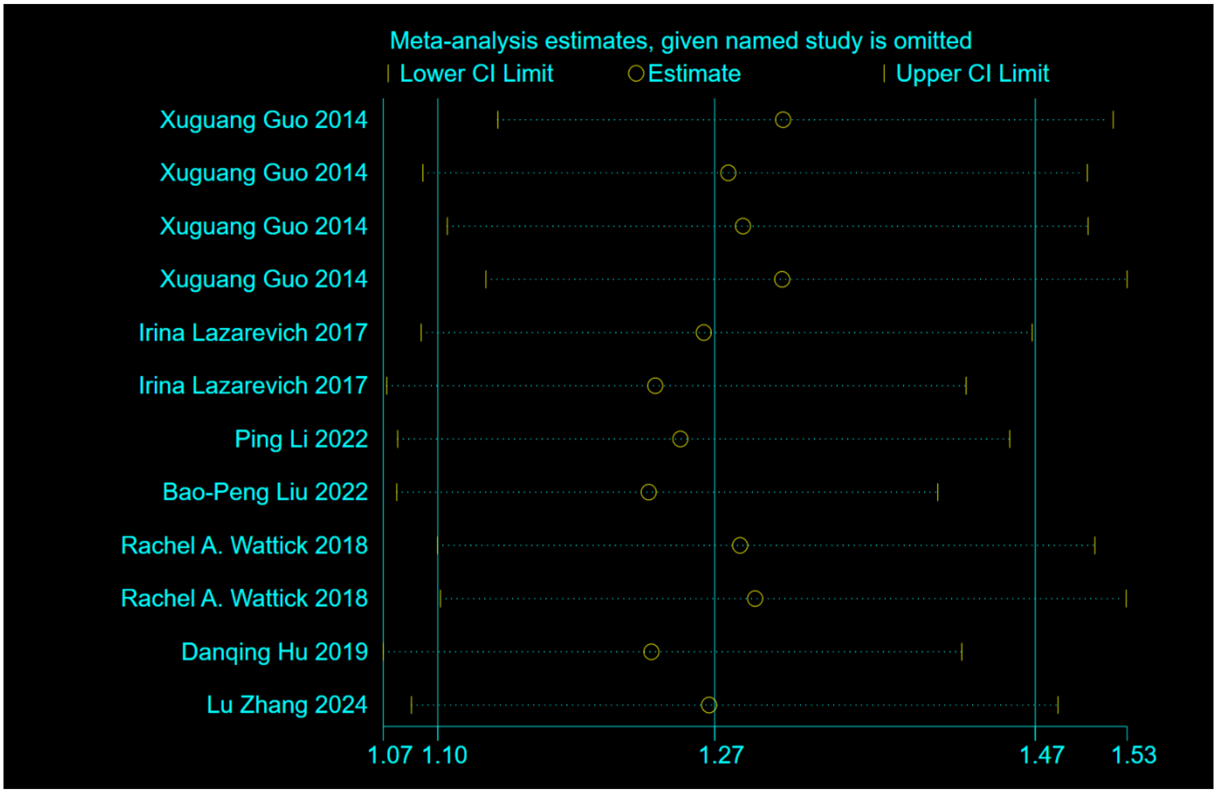

Supplementary Figure 9.2.8 Sensitivity analysis of (A) Region (Asia); (B) Region (European); (C) Region (North America).

2 Sensitivity analysis of the relationship between sugar intake and anxiety

2.1 overall analysis

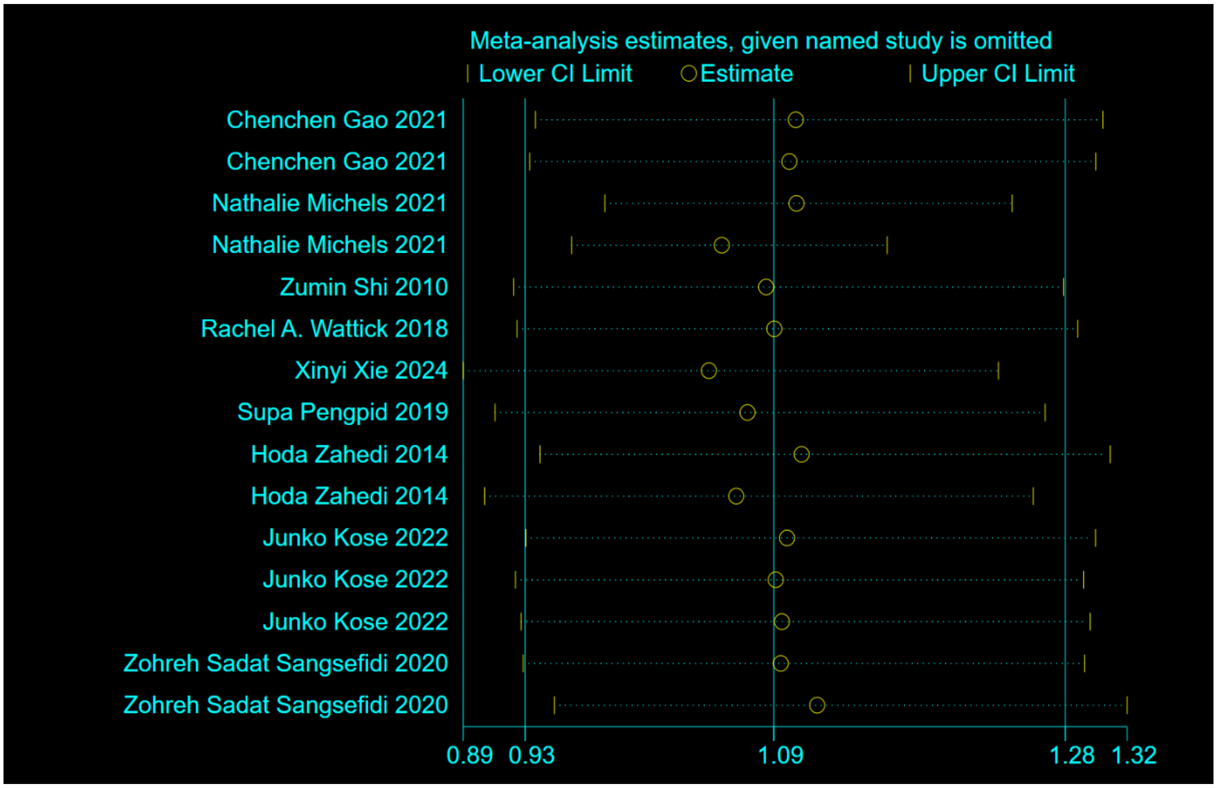

Supplementary Figure 2.1.1 Sensitivity analysis of (A) overall analysis of the relationship between sugar intake and anxiety

2.2 subgroup analysis

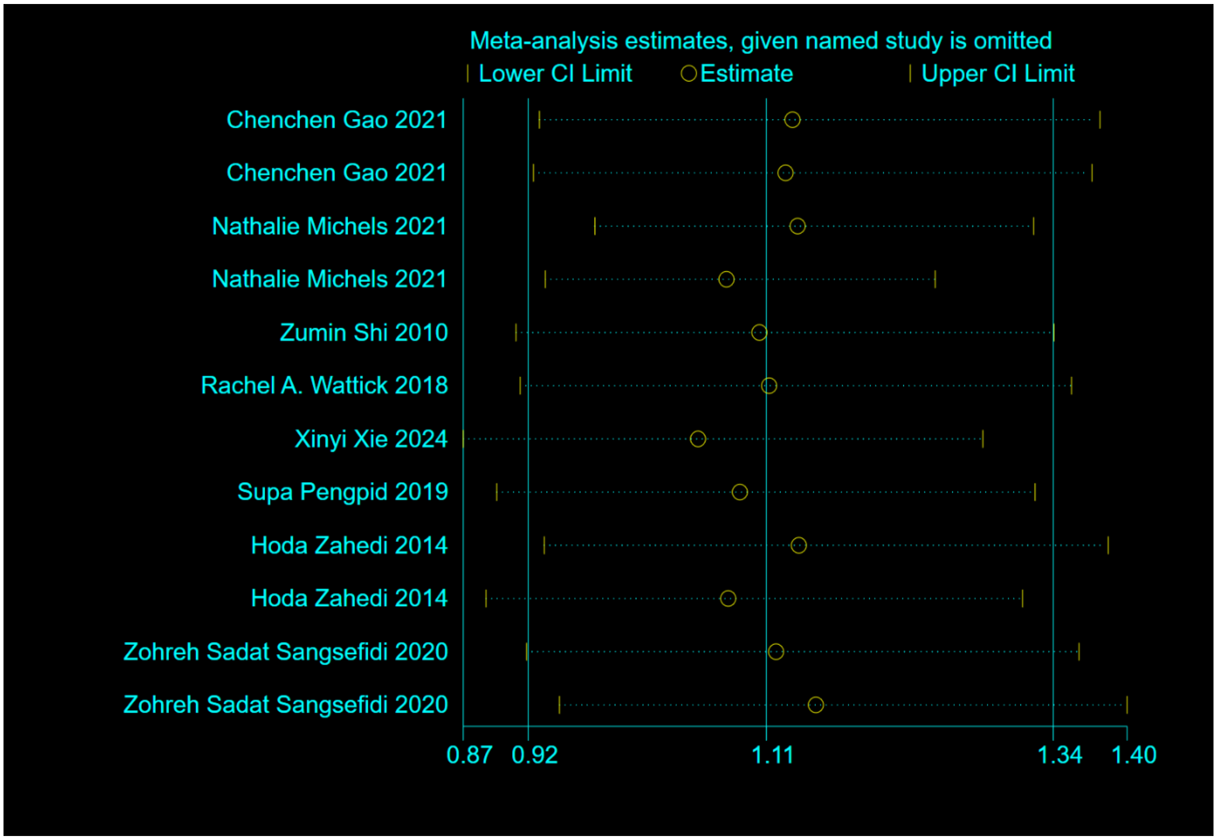

Supplementary Figure 2.2.1 Sensitivity analysis of (A) Study Designing (cross-sectional).

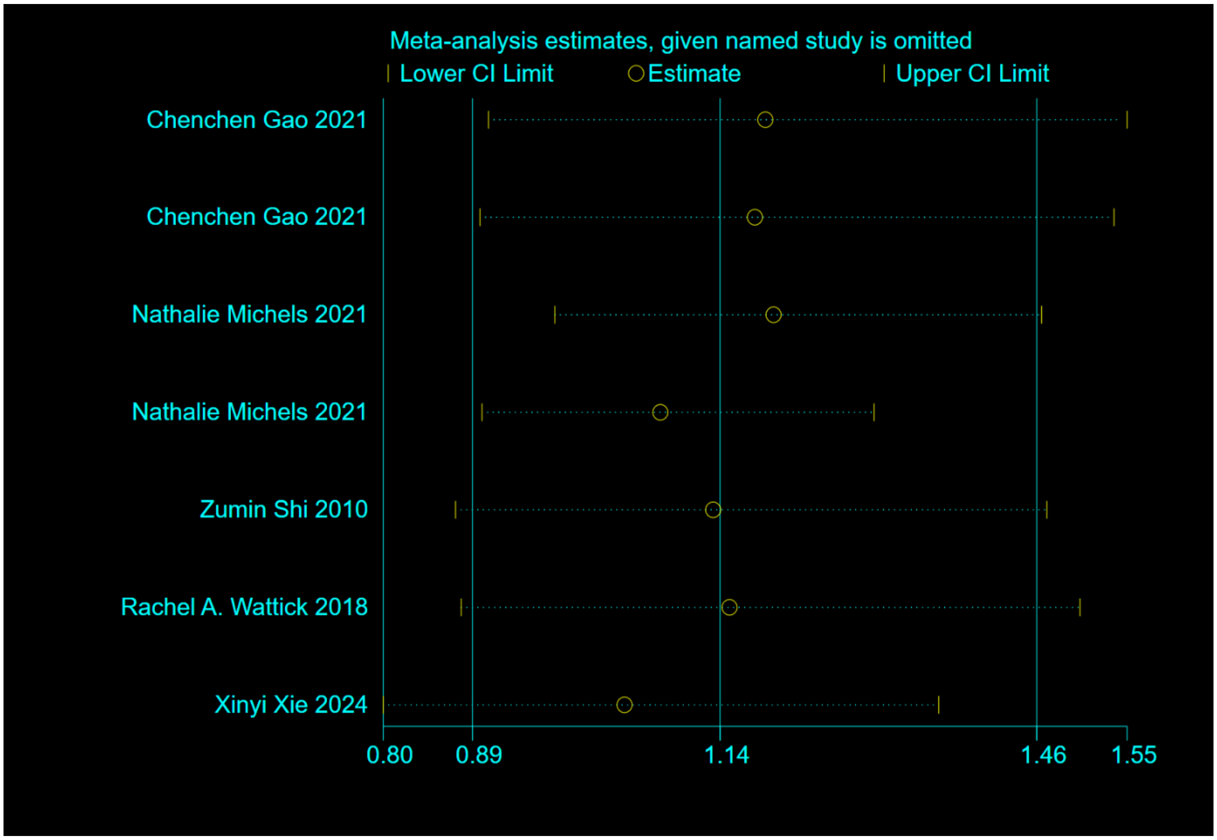

Supplementary Figure 2.2.2 Sensitivity analysis of (A) Sample Size (＜5000).

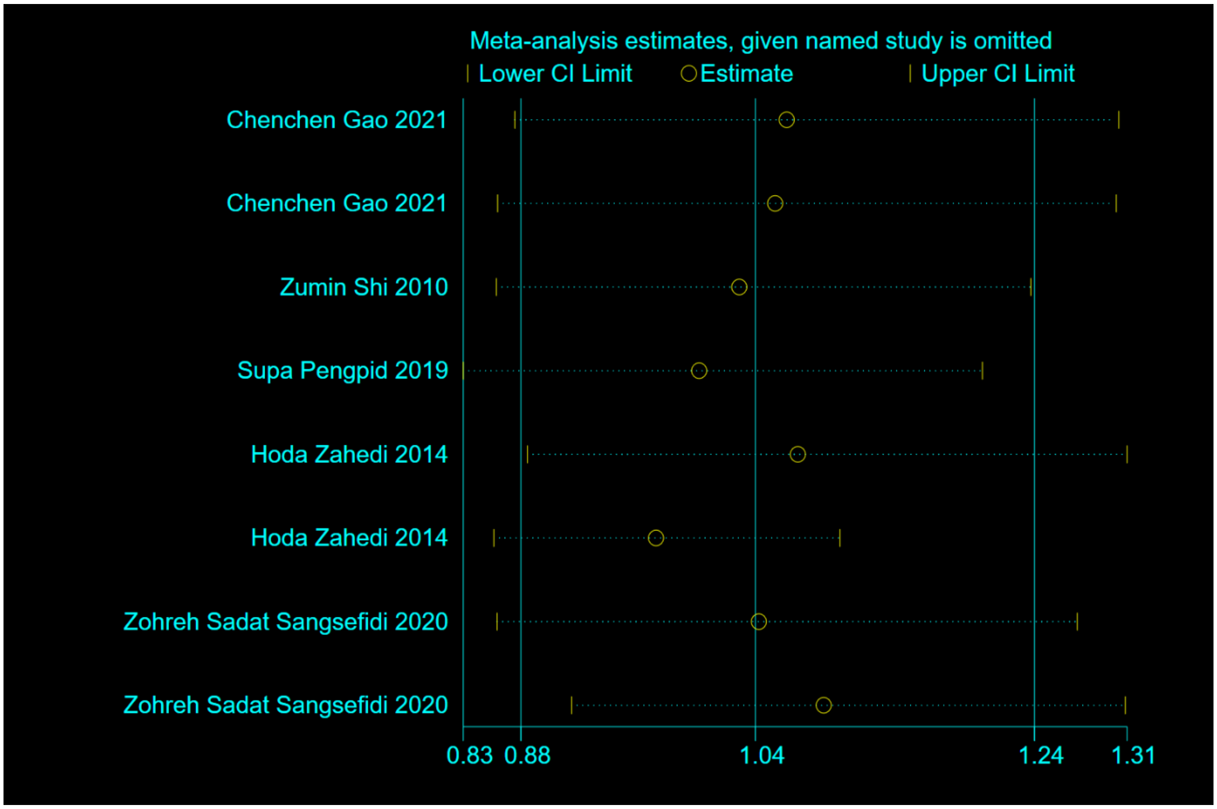

Supplementary Figure 2.2.3 Sensitivity analysis of (A) Exposure Measures (others).

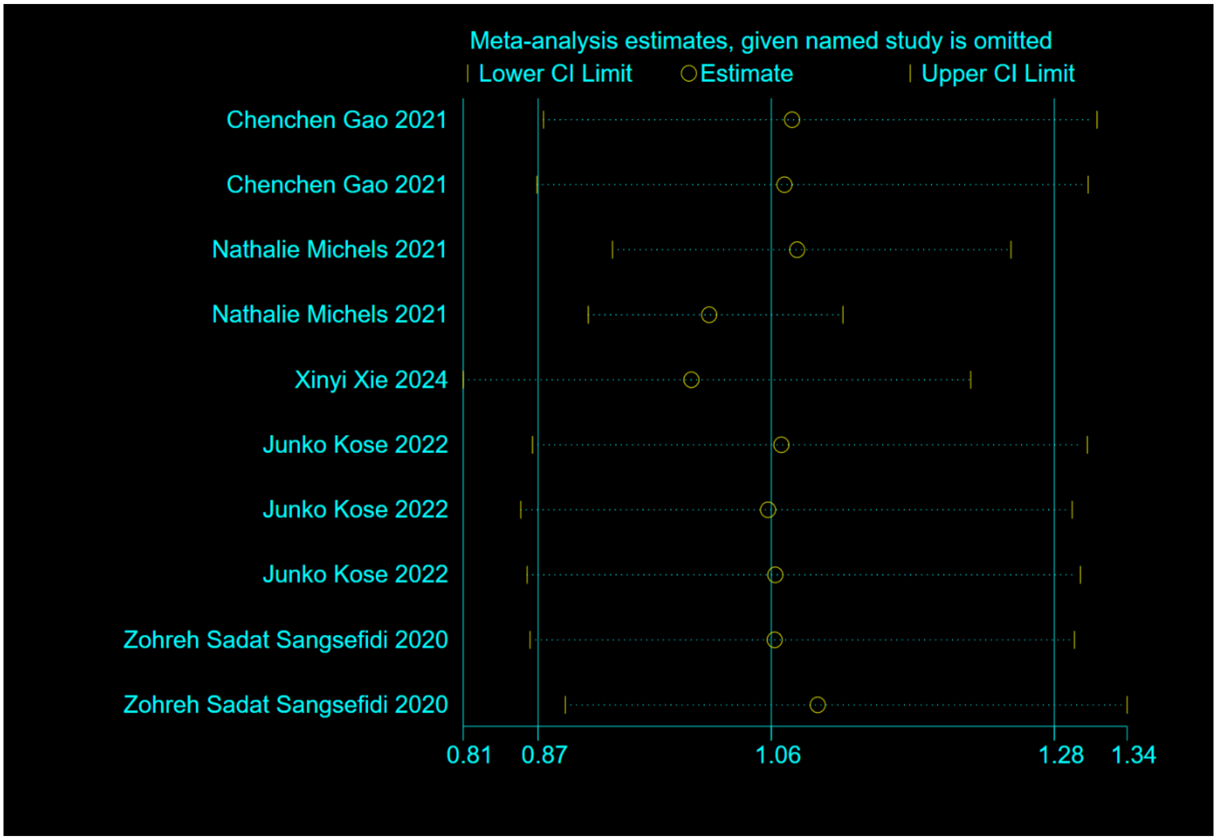

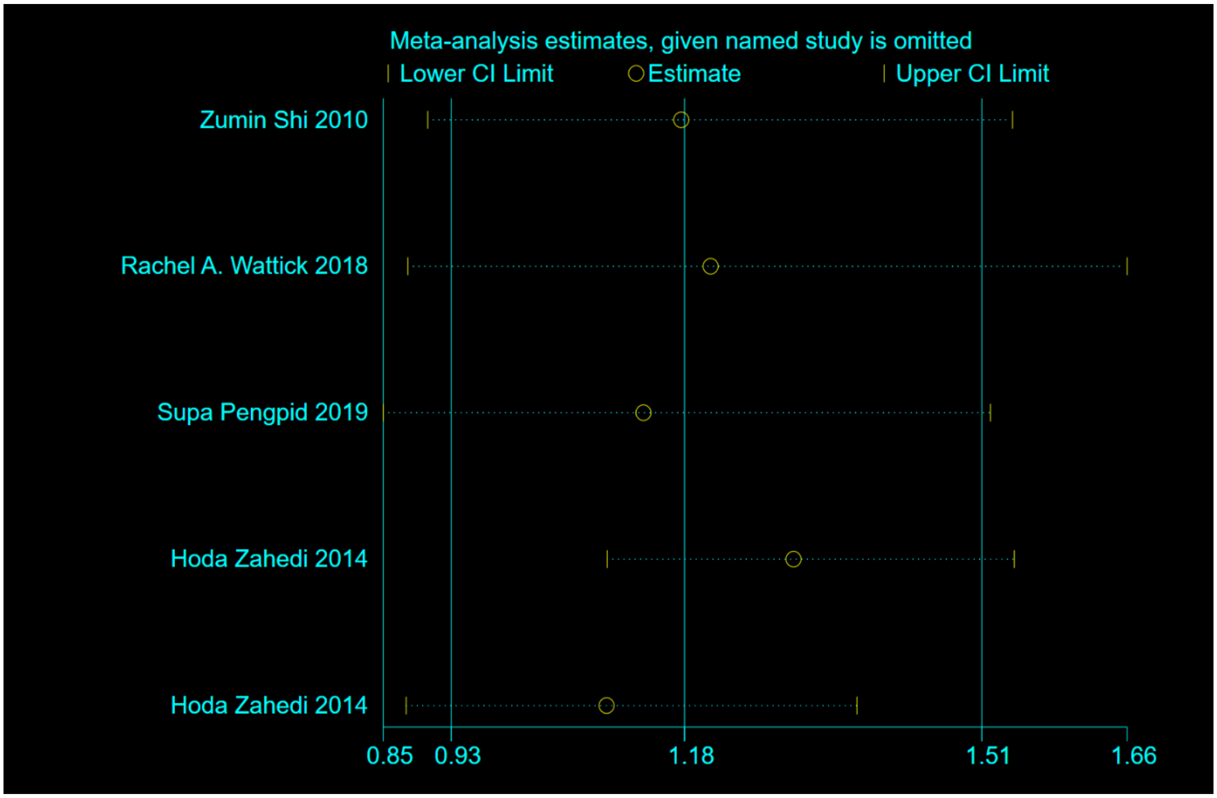

Supplementary Figure 2.2.4 Sensitivity analysis of (A) Outcome Assessment (GAD-7, SCL-90, STAI, DASS21); (B) Outcome Assessment (others).
