# Supplementary material for: Association of sugar consumption with risk of depression and anxiety: a systematic review and meta-analysis
Source: Front Nutr. 2024 Oct 16;11:1472612. doi: 10.3389/fnut.2024.1472612 (PMC11522855; doi:10.3389/fnut.2024.1472612)
Supplement: Supplementary file 4 [file Table_1.docx]

Supplementary Material

**Supplementary Table 1** Retrieval strategy sample of PubMed

| #1 | Sugars [MeSH terms] |
| --- | --- |
| #2 | Sugar* [Title/Abstract] |
| #3 | Free sugars [Title/Abstract] |
| #4 | Added sugars [Title/Abstract] |
| #5 | Sweet* [Title/Abstract] |
| #6 | Sweetening Agents [Title/Abstract] |
| #7 | Corn sweeteners [Title/Abstract] |
| #8 | Caloric sweeteners [Title/Abstract] |
| #9 | Natural sweeteners [Title/Abstract] |
| #10 | Nutritive Sweeteners [Title/Abstract] |
| #11 | High fructose corn syrup [MeSH Terms] |
| #12 | Syrup* [Title/Abstract] |
| #13 | Edible syrups [Title/Abstract] |
| #14 | Monosaccharides [MeSH Terms] |
| #15 | Monosaccharide [Title/Abstract] |
| #16 | Disaccharides [MeSH Terms] |
| #17 | Disaccharide [Title/Abstract] |
| #18 | Fructose [MeSH Terms] |
| #19 | Levulose [Title/Abstract] |
| #20 | Sucrose [MeSH Terms] |
| #21 | Saccharose [Title/Abstract] |
| #22 | Lactose [MeSH Terms] |
| #23 | Galactose [MeSH Terms] |
| #24 | D-Galactose [Title/Abstract] |
| #25 | Glucose [MeSH Terms] |
| #26 | Dextrose [Title/Abstract] |
| #27 | D-Glucose [Title/Abstract] |
| #28 | Maltose [MeSH Terms] |
| #29 | Honey [MeSH Terms] |
| #30 | Honeys [Title/Abstract] |
| #31 | Molasses [MeSH Terms] |
| #32 | Molasses [Title/Abstract] |
| #33 | Sugar-sweetened beverages [MeSH Terms] |
| #34 | Sugar-sweetened [Title/Abstract] |
| #35 | Sugar-Added beverages [Title/Abstract] |
| #36 | Soft drink [Title/Abstract] |
| #37 | Candy [MeSH Terms] |
| #38 | Candies [Title/Abstract] |
| #39 | Confection [Title/Abstract] |
| #40 | Fruit juices [Title/Abstract] |
| #41 | Or/#1-40 |
| #42 | Depression [MeSH Terms] |
| #43 | Depressive Symptom* [Title/Abstract] |
| #44 | Emotional Depression [Title/Abstract] |
| #45 | Depression disorder [Title/Abstract] |
| #46 | Anxiety [MeSH Terms] |
| #47 | Angst [Title/Abstract] |
| #48 | Social anxiet* [Title/Abstract] |
| #49 | Hypervigilance [Title/Abstract] |
| #50 | Nervousness [Title/Abstract] |
| #51 | Anxiousness [Title/Abstract] |
| #52 | Or/#42-51 |
| #53 | Case Reports [Publication Type] |
| #54 | Comment [Publication Type] |
| #55 | Congress [Publication Type] |
| #56 | Consensus Development Conference [Publication Type] |
| #57 | Duplicate Publication [Publication Type] |
| #58 | Editorial [Publication Type] |
| #59 | English Abstract [Publication Type] |
| #60 | Observational Study, Veterinary [Publication Type] |
| #61 | Retracted Publication [Publication Type] |
| #62 | Retraction of Publication [Publication Type] |
| #63 | Meta-Analysis [Publication Type] |
| #64 | Review [Publication Type] |
| #65 | Systematic Review [Publication Type] |
| #66 | Or/#53-65 |
| #67 | Humans [Species] |
| #68 | #41 and #52 not #66 and #67 |
| ((((((((((((((((((((((((((((((((((((((((sugars[MeSH Terms]) OR (sugar*[Title/Abstract])) OR (free sugars[Title/Abstract])) OR (Added sugars[Title/Abstract])) OR (sweet*[Title/Abstract])) OR (sweetening Agents[Title/Abstract])) OR (corn sweeteners[Title/Abstract])) OR (caloric sweeteners[Title/Abstract])) OR (natural sweeteners[Title/Abstract])) OR (Nutritive Sweeteners[Title/Abstract])) OR (high fructose corn syrup[MeSH Terms])) OR (syrup*[Title/Abstract])) OR (edible syrups[Title/Abstract])) OR (monosaccharides[MeSH Terms])) OR (monosaccharide[Title/Abstract])) OR (disaccharides[MeSH Terms])) OR (disaccharide[Title/Abstract])) OR (fructose[MeSH Terms])) OR (levulose[Title/Abstract])) OR (sucrose[MeSH Terms])) OR (saccharose[Title/Abstract])) OR (lactose[MeSH Terms])) OR (galactose[MeSH Terms])) OR (D-Galactose[Title/Abstract])) OR (glucose[MeSH Terms])) OR (dextrose[Title/Abstract])) OR (D-Glucose[Title/Abstract])) OR (maltose[MeSH Terms])) OR (honey[MeSH Terms])) OR (honeys[Title/Abstract])) OR (molasses[MeSH Terms])) OR (molasse[Title/Abstract])) OR (sugar-sweetened beverages[MeSH Terms])) OR (sugar-sweetened[Title/Abstract])) OR (sugar-Added beverages[Title/Abstract])) OR (soft drink[Title/Abstract])) OR (candy[MeSH Terms])) OR (candies[Title/Abstract])) OR (confection[Title/Abstract])) OR (fruit juices[Title/Abstract])) AND ((((((((((depression[MeSH Terms]) OR (Depressive Symptom*[Title/Abstract])) OR (Emotional Depression[Title/Abstract])) OR (depression disorder[Title/Abstract])) OR (anxiety[MeSH Terms])) OR (Angst[Title/Abstract])) OR (Social anxiet*[Title/Abstract])) OR (Hypervigilance[Title/Abstract])) OR (Nervousness[Title/Abstract])) OR (Anxiousness[Title/Abstract]))NOT ("case reports"[Publication Type] OR "Comment"[Publication Type] OR "Congress"[Publication Type] OR "consensus development conference"[Publication Type] OR "duplicate publication"[Publication Type] OR "Editorial"[Publication Type] OR "english abstract"[Publication Type] OR "observational study, veterinary"[Publication Type] OR "retracted publication"[Publication Type] OR "retraction of publication"[Publication Type] OR "Meta-Analysis"[Publication Type] OR "Review"[Publication Type] OR "systematic review"[Publication Type])) AND (humans[Filter]) | |
